# Recent Applications of the Multicomponent Synthesis for Bioactive Pyrazole Derivatives

**DOI:** 10.3390/molecules27154723

**Published:** 2022-07-23

**Authors:** Diana Becerra, Rodrigo Abonia, Juan-Carlos Castillo

**Affiliations:** 1 Escuela de Ciencias Química, Facultad de Ciencias, Universidad Pedagógica y Tecnológica de Colombia, Avenida Central del Norte, Tunja 150003, Colombia; diana.becerra08@uptc.edu.co; 2 Research Group of Heterocyclic Compounds, Department of Chemistry, Universidad del Valle, A.A. 25360, Cali 76001, Colombia; rodrigo.abonia@correounivalle.edu.co

**Keywords:** multicomponent reactions (MCRs), pyrazole derivatives, biological activity, medicinal chemistry, drug discovery

## Abstract

Pyrazole and its derivatives are considered a privileged *N*-heterocycle with immense therapeutic potential. Over the last few decades, the pot, atom, and step economy (PASE) synthesis of pyrazole derivatives by multicomponent reactions (MCRs) has gained increasing popularity in pharmaceutical and medicinal chemistry. The present review summarizes the recent developments of multicomponent reactions for the synthesis of biologically active molecules containing the pyrazole moiety. Particularly, it covers the articles published from 2015 to date related to antibacterial, anticancer, antifungal, antioxidant, α-glucosidase and α-amylase inhibitory, anti-inflammatory, antimycobacterial, antimalarial, and miscellaneous activities of pyrazole derivatives obtained exclusively via an MCR. The reported analytical and activity data, plausible synthetic mechanisms, and molecular docking simulations are organized in concise tables, schemes, and figures to facilitate comparison and underscore the key points of this review. We hope that this review will be helpful in the quest for developing more biologically active molecules and marketed drugs containing the pyrazole moiety.

## 1. Introduction

Multicomponent reactions (MCRs) are one-pot reactions employing three or more components to form a product, where most of the atoms of all starting materials are substantially incorporated in the final product [1]. To date, MCRs have innumerable advantages over sequential multistep synthesis such as operational simplicity, saving time and energy (step efficiency), proceeding with high convergence (process efficiency), exhibiting a very high bond-forming index (BFI), and are highly compatible with a range of unprotected orthogonal functional groups [1,2]. Thereby, MCRs are considered a powerful alternative to synthesize complex organic molecules in a high chemo-, regio-, and stereoselective manner with a plethora of applications in agrochemistry [3], polymer chemistry [4], combinational chemistry [5], medicinal chemistry [6], and especially drug discovery programs [7]. Importantly, MCRs allow the incorporation of diverse scaffold shapes by using MCR variants such as Strecker (1850) [8], Hantzsch (1881) [9], Biginelli (1891) [10], Mannich (1912) [11], Passerini (1921) [12], Kabachnik–Fields (1952) [13], Asinger (1956) [14], Ugi (1959) [15], Gewald (1966) [16], Van Leusen (1977) [17], and Groebke–Blackburn–Bienaymé (1998) [18], among others. Although most of the MCRs were discovered in the second half of the twentieth century, their use has grown enormously over the last four decades due to the demand for organic molecules in medicinal chemistry and drug discovery programs. It is estimated that approximately 5% of the commercially available drugs can be synthesized by an MCR strategy [19]. For instance, Nifedipine (Hantzsch), Telaprevir (Passerini), and Crixivan (Ugi) are valuable examples of marketed drugs obtained through this synthetic strategy [19]. 

**Figure 1:**
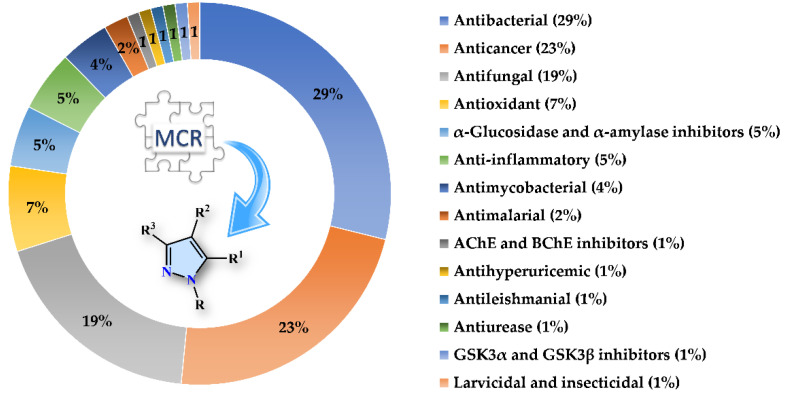
Bibliometric graphic depicting the percentage of articles associated with each biological activity screened from 2015 to date [data were collected searching in Scopus for the keywords: “pyrazole derivatives”, “biological activity”, and “multicomponent reactions”].

On the other hand, pyrazole is a “biologically privileged” five-membered *N*-heteroaromatic compound containing two nitrogen atoms in adjacent positions. Nowadays, pyrazole and its derivatives have attracted considerable attention due to their broad spectrum of pharmaceutical and biological properties [20,21,22], proving to be significant structural components of active pharmaceutical ingredients (APIs) and diverse pyrazole-based compounds. It is exemplified by the number of FDA-approved drugs containing the pyrazole scaffold, such as Celecoxib, Lonazolac, Mepirizole, Rimonabant, Difenamizole, Betazole, Fezolamine, Tepoxalin, Pyrazofurin, and Deracoxib, among other marketed drugs [20,21]. The innumerable applications in medicinal chemistry, biomedical science, and drug discovery have stimulated the academia and pharmaceutical industry for developing new, efficient, and simple synthetic protocols to prepare structurally diverse pyrazole derivatives [23,24,25]. Quite impressively, 1241 publications and 148 reviews have been reported in the Scopus database from 2015 to date searching for the keywords “pyrazole derivatives” and “biological activity”. In particular, 64 of such publications have been dedicated to the use of MCRs for the synthesis of biologically active pyrazole derivatives or at least tested for any biological properties. This review tries to provide more insight into MCR-based synthetic routes of pyrazole derivatives, as well as the analysis of some plausible synthetic mechanisms, a comprehensive picture of its diverse biological activity data, and a detailed discussion of molecular docking studies showing how pyrazole-based compounds interact with therapeutically relevant targets. Hence, this review covers articles published from 2015 to date related to antibacterial, anticancer, antifungal, antioxidant, *α*-glucosidase and *α*-amylase inhibitory, anti-inflammatory, antimycobacterial, antimalarial, and miscellaneous activities of pyrazole derivatives obtained exclusively via an MCR (Figure 1). It is worth noting that 71% of the articles found suitable for this review corresponded mainly to pyrazole derivatives displaying antibacterial (29%), anticancer (23%), and antifungal (19%) activities, respectively. For better comprehension, the content of this review has been organized and discussed through different biological activities displayed or evaluated for the target pyrazole derivatives, as shown in Figure 1.

## 2. Multicomponent Synthesis of Biologically Active Pyrazole Derivatives

### 2.1. Antibacterial Activity

**Scheme 1:**
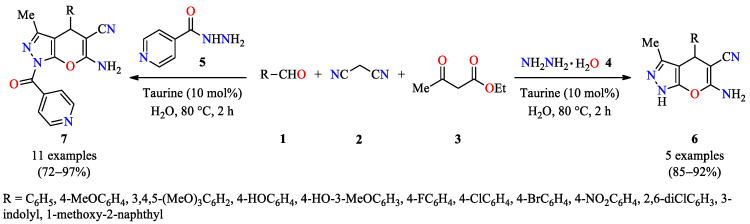
Taurine-catalyzed four-component synthesis and in silico-based analysis of 1,4-dihydropyrano[2,3-*c*]pyrazoles **6** and **7**.

**Figure 2:**
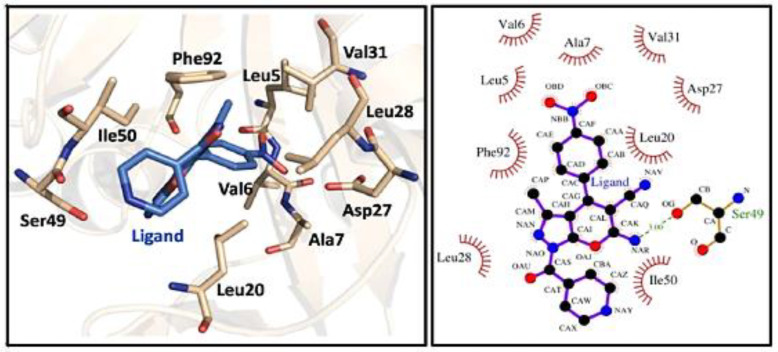
Molecular docking of compound **7** (R = 4-NO_2_C_6_H_4_) with *Staphylococcus aureus* wild-type DHFR (PDB ID: 2w9g). The left panel shows the zoomed-in view of the ligand interactions with the DHFR active site amino acid residues in the 3D space. The right panel shows the 2D representation of the array of ligand−protein interactions. Hydrogen bond formation is indicated by the green dotted line, whereas hydrophobic interactions are indicated by the spiked arcs. Image adapted from Mali et al. [26].

It is well known that over the past few decades antibiotic deposits have become less effective worldwide due to their overuse and the emerging antimicrobial drug resistance. Particularly, drug resistance has been developed by common bacterial pathogens against frequently prescribed drugs (amphotericin B, fluconazole, penicillin, and chloramphenicol, among others). Hence, it is an emerging field of study as it relates to a global health challenge, thereby numerous strategies will be required to develop new therapeutic compounds as antibacterial agents. In this regard, the broad-scale pharmaceutical applications of pyrano[2,3-*c*]pyrazole, including its appearance in numerous biologically important scaffolds, manifest its significant demand worldwide [26]. For example, the taurine-catalyzed four-component reaction of diverse (hetaryl)aldehydes **1**, malononitrile **2**, ethyl acetoacetate **3**, and hydrazine hydrate **4** in water at 80 °C for 2 h afforded 1,4-dihydropyrano[2,3-*c*]pyrazoles **6** in 85−92% yields (Scheme 1) [26]. Under the same optimized conditions, the four-component reaction was extended to isoniazid **5** leading to densely substituted products **7** in 72−97% yields. After completion of the reaction by TLC, the formed solid was filtered, washed with hot water, and recrystallized in ethanol to afford the desired product. The recyclability of the taurine catalyst was studied under optimized conditions. Accordingly, the catalyst was reused for up to three recycles without an appreciable loss of catalytic activity. This protocol is distinguished by its broad substrate scope, short reaction times, low catalyst loading, and could be applied in various organic transformations to achieve the complexity of the targeted architecture. Later, all synthesized 1,4-dihydropyrano[2,3-*c*]pyrazoles **7** were evaluated for their plausible antibacterial potential through in silico molecular docking analysis against the Staphylococcal drug target enzyme DHFR (PDB ID: 2w9g) and its trimethoprim-resistant variant S1DHFR (PDB ID: 2w9s). Remarkably, compound **7** (R = 4-NO_2_C_6_H_4_) exhibited an excellent mode of binding against DHFR and S1DHFR with a binding energy of −8.8 Kcal/mol and −8.7 Kcal/mol, respectively. As shown in Figure 2, the docked **7** (R = 4-NO_2_C_6_H_4_) at the wild-type DHFR active site forms one hydrogen bond between the amino group and Ser49 residue.

The time-efficient synthesis of pyrano[2,3-*c*]pyrazole derivatives **9** and **10** in 85−93% yields could be easily accomplished by the Knoevenagel condensation/Michael addition/imine–enamine tautomerism/*O*-cyclization sequence through a four-component reaction of (hetero)aromatic aldehydes **1**, hydrazine hydrate **4**, *β*-ketoesters **8** as ethyl acetoacetate **8a** or diethyl malonate **8b**, and enolizable active methylene compounds as malononitrile **2** or diethyl malonate **8b**, respectively, catalyzed by piperidine (5 mol%) with vigorous stirring in an aqueous medium for 20 min at room temperature (Table 1) [27]. The synthesized compounds were screened against Gram-positive (*Bacillus subtilis*, *Clostridium tetani*, and *Streptococcus pneumoniae*) and Gram-negative (*Salmonella typhi*, *Pseudomonas aeruginosa*, and *Vibrio cholerae*) bacterial strains using the broth microdilution method in the presence of ciprofloxacin as the standard drug. Remarkably, the compound **9k** displayed better activity against Gram-positive and Gram-negative bacterial strains with MIC values of 0.10−1.00 μg/mL and 0.50−2.50 μg/mL, respectively, when compared to ciprofloxacin as a standard drug (MIC = 3.12−6.25 μg/mL and 3.12 μg/mL, respectively), (Table 1). Later, the compound **9k** was evaluated for the binding mode determination and the antimicrobial in silico study against penicillin-binding protein PBPb (PDB ID: 3UDI). Molecular docking results showed that the compound **9k** has a tremendous binding affinity towards PBPb with binding of energy of −7.3 Kcal/mol. The compound **9k** was capable of forming four hydrogen bonds and five hydrophobic interactions with the key amino acid residues in the PBPb binding pocket.

In 2019, Reddy et al. developed the solvent-free synthesis of highly substituted pyrano[2,3-*c*]pyrazoles **12** in 81–91% yields through a five-component reaction of 5-methyl-1,3,4-thiadiazole-2-thiol **11**, diverse aldehydes **1**, malononitrile **2**, ethyl 4-chloro-3-oxobutanoate **8** (R = CH_2_Cl), and hydrazine hydrate **4** catalyzed by montmorillonite K10 at 65–70 °C for 5 h (Table 2) [28]. The synthesized compounds were screened against Gram-positive (*Staphylococcus aureus* and *Bacillus subtilis*) and Gram-negative (*Proteus vulgaris* and *Escherichia coli*) bacterial strains using ciprofloxacin as a standard drug. It was found that at a 50 μg/well concentration, the pyrano[2,3-*c*]pyrazoles **12** showed a diameter of growth of the inhibition zone ranging from 0 to 21 mm and 0 to 31 mm against Gram-positive and Gram-negative bacterial strains, respectively, in comparison to ciprofloxacin (21 to 23 mm and 31 to 32 mm, respectively) [28]. Interestingly, the compounds **12d** and **12f** showed better antibacterial efficacy at a 50 μg/well concentration against *Staphylococcus aureus*, *Bacillus subtilis*, *Proteus vulgaris*, and *Escherichia coli* with a zone of inhibition in the range of 15–27 mm and 16–31 mm, respectively, when compared to ciprofloxacin (21–32 mm). In addition, the minimum inhibitory concentration (MIC) and minimum bactericidal concentration (MBC) results of most active compounds **12d** and **12f** were determined against *Staphylococcus aureus*, *Bacillus subtilis*, *Proteus vulgaris*, and *Escherichia coli*. Particularly, compound **12d** showed better MIC and MBC values in the range of 12.5–50 μg/mL and 25–100 μg/mL, in comparison to ciprofloxacin (MIC = 6.25–12.5 μg/mL). It is noteworthy that compound **12d** and ciprofloxacin displayed the same MIC value (6.25 μg/mL) against *Bacillus subtilis*.

**Scheme 2:**
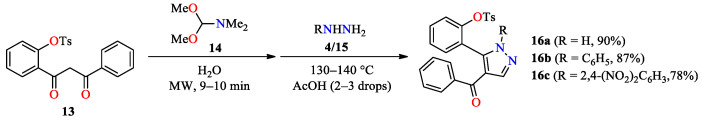
Three-component synthesis of pyrazoles **16a**–**c** with antimicrobial activity.

In 2019, Kendre’s group published the microwave-assisted synthesis of pyrazoles **16a**–**c** in 78–90% yields by the three-component reaction of 1-phenyl-3-(2-(tosyloxy)phenyl)propane-1,3-dione **13**, *N*,*N*-dimethylformamide dimethyl acetal **14**, and different amines such as hydrazine **4** (R = H) and derivatives **15b,c** catalyzed by acetic acid (2–5 drops) in water, maintaining the temperature in the range of 115–140 °C for 9–10 min (Scheme 2) [29]. The pyrazole derivatives **16** were screened against Gram-positive (*Bacillus subtilis* and *Staphylococcus aureus*) and Gram-negative (*Escherichia coli* and *Pseudomonas aeruginosa*) bacterial strains using ampicillin as the standard drug. It was found that pyrazoles **16a–c** showed an inhibition zone in the range of 10–22, in comparison to ampicillin (21–24 mm). Interestingly, compound **16c** showed better antibacterial efficacy against *Escherichia coli*, *Bacillus Subtilis*, and *Pseudomonas aeruginosa* with a zone of inhibition of 17, 20, and 22 mm, respectively, when compared to ampicillin (21, 24, and 22 mm, respectively).

Foroughifar’s group reported in 2018 the ultrasound-assisted synthesis of pyrano[2,3-*c*]pyrazoles **18** in 84−94% yields via a four-component reaction of aromatic aldehydes **1**, malononitrile **2**, ethyl 3-oxo-3-phenylpropanoate **17**, and hydrazine hydrate **4** catalyzed by graphene oxide (10 mol%) with vigorous stirring in an aqueous medium for 2–6 min at room temperature (Table 3) [30]. The recyclability of the heterogeneous catalyst was studied under optimized conditions. Accordingly, the carbocatalyst was reused in up to five recycles without an appreciable loss of catalytic activity. Later, the synthesized compounds were screened against Gram-negative (*Escherichia coli* and *Pseudomonas aeruginosa*) and Gram-positive (*Staphylococcus saprophyticus* and *Staphylococcus aureus*) bacterial strains using the broth microdilution method in the presence of cefazolin as a standard drug. The synthesized compounds showed remarkable antibacterial activity against Gram-negative and Gram-positive bacterial strains with MIC values in the range of 20−45 μg/mL, in comparison to cefazolin (MIC > 35 μg/mL). Interestingly, the compounds **18a** and **18h** displayed the highest activity against Gram-negative bacterial strains with MIC values ranging from 20 to 25 μg/mL, while the compounds **18a**, **18f**, and **18g** displayed the highest activity against Gram-positive bacterial strains with MIC values ranging from 20 to 25 μg/mL, in comparison to cefazolin (MIC > 35 μg/mL).

**Figure 3:**
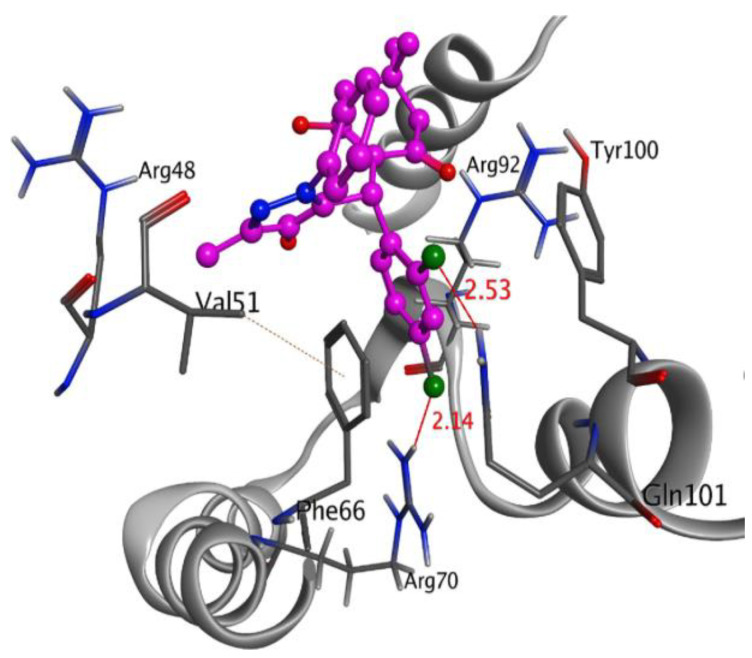
Docking molecular of the compound **21a** (magenta) with crucial residues of thymidylate kinase target protein (PDB ID: 4QGG) from *Staphylococcus aureus*. Image adapted from Barakat et al. [31].

In 2018, Bakarat et al. reported the synthesis of pyrazole-dimedone derivatives **21** in 40–78% yields via a four-component reaction of 3-methyl-1-phenyl-1*H*-pyrazol-4(5*H*)-one **19**, diverse aldehydes **1**, and dimedone **20** mediated by Et_2_NH in water at an ambient temperature for 1–12 h (Table 4) [31]. The synthesized pyrazolic salts **21** were evaluated against three Gram-positive bacterial strains including *Staphylococcus aureus*, *Enterococcus faecalis*, and *Bacillus subtilis* using ciprofloxacin as the standard drug. The pyrazolic salts **21a–o** showed a diameter of growth of the inhibition zone in the range of 10–24 mm and MIC values in the range of 8–64 μg/L against all Gram-positive bacterial strains, in comparison to ciprofloxacin (24–27 mm and ≤0.25 μg/L, respectively). Overall, the pyrazolic salts **21e** and **21l** were the most active compounds against *Staphylococcus aureus* with a MIC value of 16 μg/L. Moreover, compound **21b** was the most active against *Enterococcus faecalis* with a MIC value of 16 μg/L. Ultimately, compound **21k** displayed better antibacterial efficacy against *Bacillus subtilis* with a zone of inhibition of 20 mm and a MIC value of 8 μg/L, when compared to ciprofloxacin (25 mm and ≤0.25 μg/L, respectively). Later, a docking simulation was performed to predict the mode of inhibition against the thymidylate kinase (TMK) (PDB ID: 4QGG) from *Staphylococcus aureus*. Although compound **21a** was moderately active against the *Staphylococcus aureus* bacterial strain, it showed more interactions with the TMK protein from *Staphylococcus aureus*. The docking molecular of **21a** displayed the highest negative score of −6.86 kcal/mol, which is comparable to ciprofloxacin (−6.90 kcal/mol). As shown in Figure 3, the chlorine atoms at the 2 and 4 positions were engaged in the formation of two halogen bonds with the amino groups of Arg70 and Gln101, respectively. Moreover, the dichloro-substituted benzene ring along with the pyrazole ring displayed various π–π and π–cation interactions with the crucial residues Phe66 and Arg92. Apart from this, the carbon atom located at the R position and methyl of the pyrazole ring formed hydrophobic interactions with Arg48 and Phe66. Unfortunately, the authors did not show the docking molecular for compounds **21e** and **21l**, which were the most active against *Staphylococcus aureus* with a MIC value of 16 μg/L (Table 4).

On the other hand, an efficient one-pot multicomponent reaction of 3-(2-bromoacetyl)coumarins **22**, thiosemicarbazide **23**, and substituted acetophenones **24** in *N*,*N*-dimethylformamide, followed by Vilsmeier–Haack formylation reaction conditions, afforded the coumarin-containing thiazolyl-3-aryl-pyrazole-4-carbaldehydes **25** with high yields and short reaction times [32]. During this approach, thiazole and pyrazole rings are formed along with a functional group (-CHO) on the pyrazole ring in a regioselective manner. Subsequently, products **25** were screened against Gram-negative (*Escherichia coli*, *Klebsiella pneumoniae*, and *Proteus vulgaris*) and Gram-positive (*Methicillin-resistant Staphylococcus aureus*, *Bacillus Subtilis*, and *Bacillus cereus*) bacterial strains by using the agar well diffusion method in the presence of gentamycin sulfate and ampicillin as standard drugs [32]. As shown in Table 5, the synthesized compounds **25** showed low to moderate antibacterial activity with MIC values ranging from 72.8 to 150 μg/mL, in comparison to gentamycin sulfate (MIC = 2–45 μg/mL) and ampicillin (MIC = 4–10 μg/mL) as standard drugs. In particular, the compound **25n** showed moderate MIC values of 86.5, 79.1, and 72.8 μg/mL against *Escherichia coli*, *Klebsiella pneumonia*, and *Bacillus cereus*, respectively. Similarly, the compound **25m** also exhibited a moderate MIC value of 98.2 μg/mL against *Bacillus subtilis*.

Interestingly, a series of highly functionalized spiropyrrolidine-oxindoles **29** in 80–93% yields have been synthesized through a 1,3-dipolar cycloaddition reaction between dipolarophile (*E*)-3-(1,3-diphenyl-1*H*-pyrazol-4-yl)-2-(1*H*-indole-3-carbonyl)acrylonitrile **26** and an azomethine ylide formed in situ from isatin derivatives **27** and amino acids such as *L*-proline **28a** and *L*-thioproline **28b** in refluxing methanol for 2 h (Table 6) [33]. The reaction mixture was allowed to cool at room temperature, filtered, and the resulting crude was recrystallized in ethanol to afford spiropyrrolidine-oxindoles **29** in a diastereoselective manner. The spiropyrrolidine-oxindoles **29** were screened against Gram-negative (*Salmonella typhimurium*, *Klebsiella pneumoniae*, *Proteus vulgaris*, *Shigella flexneri*, and *Enterobacter aerogenes*) and Gram-positive (*Micrococcus luteus*, *Staphylococcus epidermidis*, *Staphylococcus aureus*, and *Methicillin-resistant Staphylococcus aureus*) bacterial strains using the disc diffusion method in the presence of streptomycin as a standard drug [33]. The synthesized compounds showed low to moderate antibacterial activity against Gram-negative and Gram-positive bacterial strains with MIC values in the range of 31.2–500 μg/mL, in comparison to streptomycin (MIC = 6.25–30 μg/mL). Interestingly, compound **29a** displayed the highest activity against Gram-negative bacterial strains with MIC values in the range of 31.2–125 μg/mL, except for *Salmonella typhimurium* (MIC = 250 μg/mL). However, compounds **29c** and **29e** showed the highest activity against *Salmonella typhimurium* with a MIC value of 125 μg/mL. Furthermore, compound **29a** showed better activity against Gram-positive bacterial strains with a MIC value of 31.2 μg/mL, except for *Staphylococcus epidermidis* (MIC = 62.5 μg/mL).

**Scheme 3:**
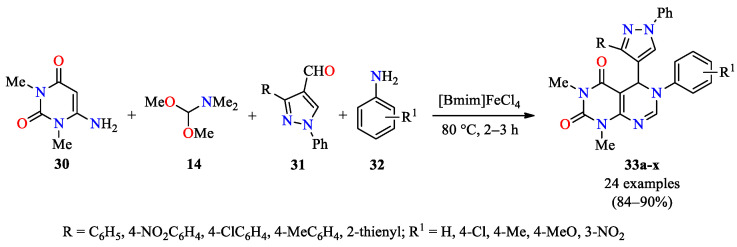
Four-component synthesis of pyrazole-based pyrimido[4,5-*d*]pyrimidines **33** mediated by [Bmim]FeCl_4_.

In 2017, Suresh et al. described an efficient synthesis of pyrazole-based pyrimido[4,5-*d*]pyrimidines **33** in 84–90% yields through a four-component reaction from 6-amino-1,3-dimethyluracil **30**, *N*,*N*-dimethylformamide dimethyl acetal **14**, 1-phenyl-3-(4-substituted-phenyl)-4-formyl-1*H*-pyrazoles **31**, and primary aromatic amines **32** using the ionic liquid [Bmim]FeCl_4_ as both catalyst and solvent at 80 °C for 2–3 h (Scheme 3) [34]. Although the reaction was optimized with various solvents such as water, ethanol, acetic acid, DMF, nitrobenzene, and toluene under reflux or heating conditions, the yields were disappointing. However, the use of ionic liquids was conducted to improve the yields. An interesting trend was observed with the types of substituents on 1-phenyl-3-(4-phenyl)-4-formyl-1*H*-substituted pyrazoles **31** and primary aromatic amines **32**. Overall, the presence of electron-withdrawing substituents (85–90%) generated higher yields than electron-donating substituents (78–84%). Finally, the ionic liquid catalyst was reused in up to four cycles without an appreciable loss in catalytic activity.

**Scheme 4:**
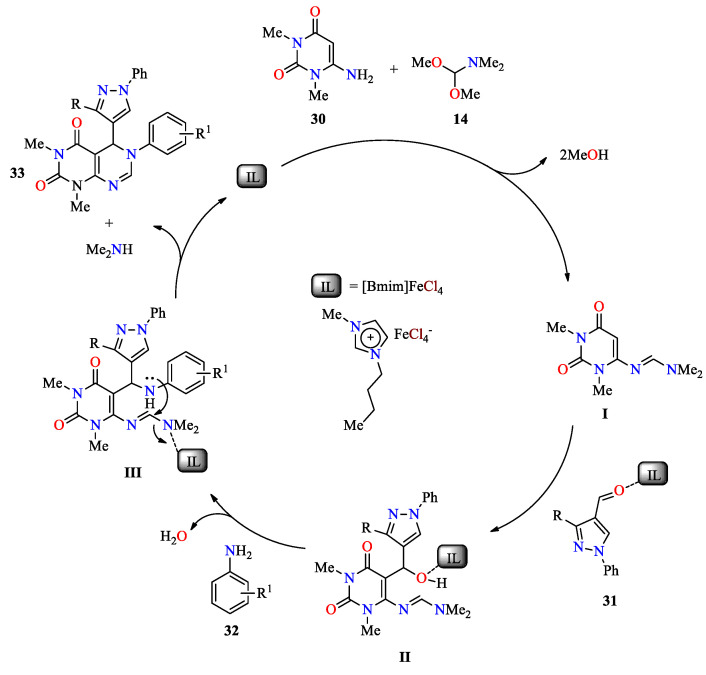
Proposed mechanism for the synthesis of pyrazole-based pyrimido[4,5-*d*]pyrimidines **33** mediated by [Bmim]FeCl_4_ (IL).

The plausible mechanism for the synthesis of pyrazole-based pyrimido[4,5-*d*]pyrimidines **33** is illustrated in Scheme 4. Initially, the [Bmim]FeCl_4_-catalyzed condensation reaction between 6-amino-1,3-dimethyluracil **30** and *N*,*N*-dimethylformamide dimethyl acetal **14** generated the amidine intermediate **I**, which reacted with 1-phenyl-3-(4-substituted-phenyl)-4-formyl-1*H*-pyrazole **31** to furnish intermediate **II**. Then, the [Bmim]FeCl_4_-catalyzed nucleophilic substitution of the hydroxyl group of the intermediate **II** by the amine group of the aromatic amine **32** gave the intermediate **III**, which participated in the intramolecular nucleophilic addition/elimination of the Me_2_NH sequence to form the pyrimido[4,5-*d*]pyrimidine system **33**.

The synthesized compounds **33** were screened for their antibacterial activity against Gram-positive (*Bacillus subtilis*, *Staphylococcus aureus*, *Staphylococcus aureus MLS-16*, and *Micrococcus luteus*) and Gram-negative (*Klebsiella planticola*, *Escherichia coli*, and *Pseudomonas aeruginosa*) bacterial strains using ciprofloxacin as a positive control. Overall, the compounds **33c** (R = 4-NO_2_C_6_H_4_, R^1^ = 4-Me), **33l** (R = 4-MeC_6_H_4_, R^1^ = 4-Cl), and **33m** (R = 4-MeC_6_H_4_, R^1^ = 4-Me) were the most active against *Bacillus subtilis*, *Staphylococcus aureus*, *Staphylococcus aureus MLS16*, and *Micrococcus luteus* with MIC values in the range of 3.9–15.6 µg/mL, in comparison to ciprofloxacin (MIC = 0.9 µg/mL). Notably, the compound **33l** showed better activity against *Bacillus subtilis* and *Staphylococcus aureus* with an MBC value of 7.8 µg/mL, when compared to ciprofloxacin (MBC = 0.9 and 1.9 µg/mL, respectively).

Very recently, a series of 4-[(3-aryl-1-phenyl-1*H*-pyrazol-4-yl)methylidene]-2,4-dihydro-3*H-*pyrazol-3-ones **34** were synthesized through a one-pot three-component reaction of 3-aryl-1-phenyl-1*H*-pyrazole-4-carbaldehydes **31**, ethyl acetoacetate **3**, and hydrazine hydrate **4** in the presence of sodium acetate as a base under refluxing ethanol for 1 h (Table 7) [35]. After completion of the reaction (TLC), the mixture was cooled to room temperature, and the precipitate was filtered, washed with water, dried, and purified by column chromatography to afford compounds **34** in 82–92% yields. All synthesized compounds **34** were screened against Gram-positive (*Staphylococcus aureus* and *Bacillus subtilis*) and Gram-negative (*Pseudomonas aeruginosa* and *Escherichia coli*) bacterial strains using norfloxacin as a positive control. These compounds showed acceptable antibacterial activity against Gram-positive and Gram-negative bacterial strains with a zone of inhibition in the range of 7.9–17.2 mm, when compared to norfloxacin (19.2–25.6 mm). Interestingly, compound **34b** exerted the better antibacterial potency against *Staphylococcus aureus* with a zone of inhibition of 17.2 mm, while the compound **34a** displayed the highest activity against *Bacillus subtilis*, *Pseudomonas aeruginosa*, and *Escherichia coli* with a zone of inhibition of 12.0, 13.5, and 14.3 mm, respectively.

**Figure 4:**
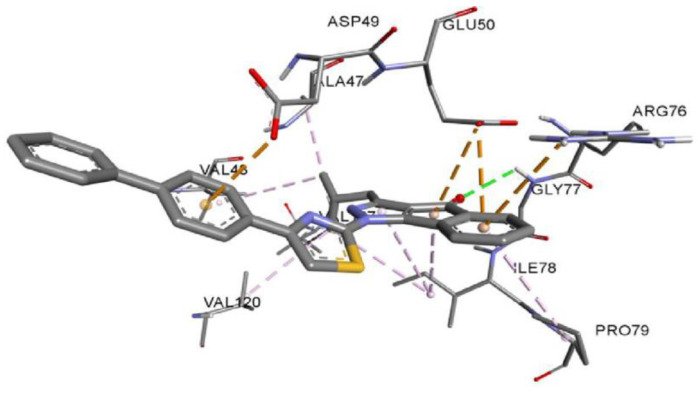
Docking molecular of indenopyrazole **37d** with crucial residues of DNA gyrase of *Escherichia coli* (PDB ID: 1KZN). Image adapted from Mor et al. [36].

A series of thiazole-tethered indenopyrazoles **37** were efficiently synthesized through a one-pot three-component reaction (Table 8) [36]. Initially, a mixture of 2-acyl-(1*H*)-indene-1,3-(2*H*)-diones **35** and thiosemicarbazide **23** in dry methanol was refluxed for 10–15 min. Thereafter, sodium acetate, *α*-bromoketones **36**, and methanol/glacial acetic acid (2:1, *v/v*) were slowly added. The resulting reaction mixture was refluxed for 5–8 h. On completion of the reaction, the formed solid was filtered and purified by column chromatography to afford the corresponding indenopyrazoles **37** in 53–80% yields (Table 8). All synthesized compounds **37** were screened for their antimicrobial activity against Gram-positive (*Staphylococcus aureus* and *Bacillus subtilis*) and Gram-negative (*Pseudomonas aeruginosa* and *Escherichia coli*) bacterial strains showing MIC values in the range of 0.0270–0.0652 μmol/mL and 0.0270–0.0629 μmol/mL, respectively, when compared to ciprofloxacin (MIC = 0.0094 μmol/mL) as a positive control. Interestingly, the indenopyrazole **37d** displayed the highest potency against all the tested microbial strains with MIC values ranging from 0.0270 to 0.0541 μmol/mL, in comparison to the standard drug ciprofloxacin (MIC = 0.0094 μmol/mL). To determine the binding conformation, molecular docking of indenopyrazole **37d** was performed in the binding site of DNA gyrase of *Escherichia coli* (PDB ID: 1KZN). As shown in Figure 4, the carbonyl group of indene moiety forms one hydrogen bond with Gly77. The pyrazole ring interacts with Ile78 via π–alkyl interactions, while the phenyl group present on the thiazole ring interacts with Asp49 via π–anion interactions. Finally, the π–electrons of the indene moiety interact with Glu50 and Arg76 via π–anion and π–cation interactions, respectively.

The Ugi multicomponent reaction (MCR) is one of the most predominant isocyanide-based MCRs, attracting a wide diversity of fascinated chemists owing to its four-component transformation and remarkable functional tolerance [37,38]. Viewing the significance of the *N*-containing heterocycles (particularly pyrazole and isoquinolone derivatives) in countless areas, mainly in medicinal chemistry, a Ugi-mediated research work focused on the synthesis of the hybrid molecules of these two structural pharmacophores was undertaken [39]. Thus, the microwave-assisted ligand-free palladium-catalyzed post-Ugi reaction for the synthesis of isoquinolone and pyrazole-mixed pharmacophore derivatives **41** was achieved from pyrazole-substituted amides **40** (synthesized using the Ugi reaction). In this approach, intermediate amides **40** were obtained in 77−96% yield from an Ugi four-component type reaction of anilines **32**, *t*-butyl isocyanide **38**, benzaldehydes **1**, and a variety of pyrazole carboxylic acids **39** in MeOH at 40 °C for approximately 18 h, as shown in Table 9. Subsequently, after optimization of the reaction conditions, the isocyanide **38** insertion and successive intramolecular cyclization process of the intermediates **40** was achieved in the presence of PdCl_2_ and Cs_2_CO_3_ as a base in DMF as the solvent at 150 °C under microwave irradiation, affording the target pyrazole derivatives **41**. It was found that irrespective of the substituents on the pyrazole scaffold in **40**, all the representative reactions could produce moderate to good yields of **41**. Moreover, among the various isocyanides **38** being tested with **40** for this transformation, only *t*-butyl isocyanide **38** was found to be constructed efficiently in the hybrid structures **41** and hence it was the only isocyanide employed in this investigation. Then, the synthesized target compounds **41** were subjected to in vitro antibacterial activity against five clinical bacterial strains (i.e., *Staphylococcus aureus*, *Escherichia coli*, *Enterococcus faecalis*, *Streptococcus pyogens*, and *Vibrio cholera*), according to Clinical and Laboratory Standard Institute (CLSI) protocols [40]. The activities of **41** in terms of their MICs ranged from 250 μM to 20.85 μM, as shown in Table 9. The results obtained were further described with the help of DFT and molecular orbital calculations, showing that pyrazole derivatives **41e** and **41g** revealed good antibacterial activity compared to the standard drug kanamycin.

On the basis that 4*H*-pyrans and 4*H*-pyran-annulated heterocyclic frameworks represent an excellent structural motif that is often found in naturally occurring compounds with a broad spectrum of remarkable biological activities, [41,42] a library of spiropyrans **45** were synthesized via a one-pot four-component reaction of various-type cyclic CH-acids **44**, malononitrile **2**, cyanoacetohydrazide **42**, and ninhydrin **43** in EtOH at reflux under catalyst-free conditions, as shown in Table 10 [43]. The obtained products **45** were subsequently tested in vitro for antibacterial effects on the Gram-negative strain *Escherichia coli* and the Gram-positive strain *Staphylococcus aureus* by using the disk diffusion method and using tetracycline and DMSO as positive and negative controls, respectively. Results showed an inhibition zone of 4–15 mm for compounds **45** against *Staphylococcus aureus* (except **45d**, **45h**, and **45j**), while *Escherichia coli* was resistant against all compounds **45** tested (Table 10). Moreover, it was found that compounds **45a**, **45b**, **45f**, **45g**, and **45i** displayed the best MICs against *Staphylococcus aureus*.

As mentioned before, pyran-annulated heterocyclic compounds have interested synthetic organic chemists and biochemists because of their biological [44] and pharmacological activities [45]. Additionally, many chemical reactions have used ionic liquids such as green alternative media for volatile organic solvents because of their low vapor pressures, chemical and thermal stability, nonflammability, high ionic conductivity, and wide electrochemical potential window [46]. Based on the above findings, a simple and highly efficient synthesis of a series of pyrano[2,3-*c*]pyrazole-5-carbonitrile derivatives **46** by a one-pot, four-component reaction with ethyl benzoylacetate **17**, malononitrile **2**, aryl aldehydes **1**, and hydrazine hydrate **4** was achieved [47]. Reactions were performed under several catalytic conditions such as choline chloride:thiourea (DES) and choline chloride:urea as ionic liquid catalysts under reflux and ultrasonic irradiation conditions in short reaction times with high yields (Table 11). It was also observed that the best results according to the reaction conditions in the presence of catalyst choline were with chloride:thiourea, because of the acidic strength of thiourea compared with urea. Overall, the method provided several advantages such as a shorter reaction time with high yields, mild reaction conditions, and environmental friendliness. Furthermore, all compounds **46** were subsequently evaluated for their in vitro antibacterial activity against two Gram-positive bacteria (*Staphylococcus saprophyticus* and *Staphylococcus aureus*) and two Gram-negative bacteria (*Escherichia coli* and *Pseudomonas aeruginosa*), compared with cefazolin regarding the minimum inhibitory concentration (MIC). Interestingly, several of the obtained compounds **46** were more active than cefazolin (MIC values < 35), as shown in Table 11.

Due to functionalized coumarins playing a prominent role in medicinal chemistry and being intensively used as scaffolds for drug development [48,49], an efficient synthesis of a series of pyrazolylbiscoumarin **47** and pyrazolylxanthenedione **48** hybrid derivatives was established [50]. The synthesis of the title compounds **47** was achieved using a simple acid-catalyzed pseudo three-component condensation reaction of the corresponding 1*H*-pyrazole-4-carbaldehydes **31** and two equivalents of type 4-hydroxycoumarin **44** in refluxing ethanol in the presence of concentrated HCl, as shown in Table 12. Similarly, treatment of 1*H*-pyrazole-4-carbaldehydes **31** and two equivalents of dimedone **20** under the same reaction conditions afforded the pyrazolylxanthenedione derivatives **48**, as shown in Table 12. All the synthesized compounds **47** and **48** were screened for their antibacterial activity against the Gram-positive bacteria *Staphylococcus aureus* and the Gram-negative bacteria *Klebsiella pneumoniae*, using the commercial antibiotic streptomycin as the standard drug. The tested compounds exhibited a variable degree of antibacterial activity, showing the inhibition zone size ranging from 6 to 21 mm as shown in Table 12. Particularly, compounds **47a** and **48a** displayed higher biological activity; however, none of the synthesized compounds **47**/**48** were superior to the standard drug streptomycin.

Due to fluorine playing a crucial role in improving pharmacodynamic and pharmacokinetic properties of drugs molecules [51], and considering the fact that fluoro-substituted pyrazoles are a class of heterocycles occupying a remarkable position in medicinal chemistry because of their variety of pharmacological activities [52,53], a multicomponent cyclocondensation reaction was implemented for the synthesis of a series of fluorinated 5-(phenylthio)pyrazole-based polyhydroquinoline derivatives **51** [54]. This approach proceeded by incorporating various fluorinated enaminones **49**, different active methylene compounds **50**, and phenylthio-1-phenyl-1*H*-pyrazole-4-carbaldehydes **31** in a one-pot process in the presence of piperidine as a basic catalyst under refluxing EtOH, affording the targeted compounds **51** in good to excellent yield (71−84%), as shown in Table 13. All the synthesized compounds **51** were evaluated in vitro for their antibacterial activity using the broth microdilution method according to National Committee for Clinical Laboratory Standards [55]. Three Gram-positive (*Bacillus subtilis*, *Clostridium tetani*, and *Streptococcus pneumoniae*) and three Gram-negative (*Salmonella typhi*, *Escherichia coli*, and *Vibrio cholerae*) bacteria were chosen for antibacterial screening using ampicillin, ciprofloxacin, norfloxacin, and chloramphenicol as the standard antibacterial agents. The results indicated that compound **51** showed moderate to very good antibacterial activity in comparison with the activity displayed by the standard drugs, as shown in Table 13.

It is known that pyrazoles, imidazoles, dihydropyrimidinones (DHPMs), and 1,4-dihydropyridines (DHPs) are considered to be important chemical synthons of various physiological significance and pharmaceutical utility [56,57]. Based on these precedents, Viveka, et al. considered constructing hybrid molecular architectures by combining pyrazole with biologically active DHPMs, DHPs, and imidazole pharmacophores through a multicomponent reaction sequence [58]. In this sense, a series of pyrazole-containing pyrimidine **52**, 1,4-dihydropyridine **53**, and imidazole **54** derivatives were synthesized in acceptable to good yields using substituted type 4-formylpyrazole **31** as a key intermediate by following the Biginelli reaction, the classical Hantzsch condensation, and the Debus reaction, respectively, as described in Table 14. The synthesized products were screened for their in vitro antibacterial properties by the disc diffusion method against *Staphylococcus aureus*, *Escherichia coli*, *Pseudomonas aeruginosa*, and *Klebsiella pneumoniae* using streptomycin as the standard drug. The activities and the minimum inhibitory concentration (MIC) of the pyrazole derivatives **52**, **53**, and **54** are presented in Table 14. In general, the results showed that the various compounds **52**, **53**, and **54** displayed variable inhibitory effects on the growth of the bacteria depending on the nature of the substituents with the pyrazole; particularly, compounds **52c** and **52f** displayed the highest activity. Additionally, from these studies, it is concluded that pyrimidinone-incorporated halo-substituted 1,3-diarylpyrazole was shown to be a better molecule from a biological activity point of view, and these molecules could be designed as potential drugs after further structural modifications.

Research on the design of synthetic protocols with an efficient atom economy and catalyst-free under ultrasound irradiation conditions has received specific attention [59]. In this sense, an efficient multicomponent method for the synthesis of biologically active 1,3,4-thiadiazole-1*H*-pyrazol-4-yl thiazolidin-4-one hybrids **57** was reported [60], from the reaction of 5-(substituted phenyl)-1,3,4-thiadiazol-2-amines **55**, type pyrazole-4-carbaldehyde **31**, and 2-mercaptoacetic acid **56** in ethanol under ultrasound irradiation. It was found that reactions gave the desired products **57** in 91–97% yield, in short times (55–65 min) at 50 °C as shown in Table 15. All the obtained pyrazol-4-yl-thiazolidin-4-ones **57** were screened for their antibacterial activity. Among the screened hybrids **57**, derivatives with 4-nitro and 3-nitro groups substituted on the phenyl ring showed fourfold (MBC = 156.3 μg/cm^3^) and twofold (MBC = 312.5 μg/cm^3^), respectively, stronger potency against the *Pseudomonas aeruginosa* strain as compared to the standard ciprofloxacin. Moreover, SAR studies revealed the importance of the functional groups on the phenyl ring of the 1,3,4-thiadiazol-2-amine moiety for varying bacterial activity. Thus, the electron-withdrawing (NO_2_) group at *para*- and *meta*-positions played a significant role in enhancing the antibacterial activity, as shown in Table 15.

Among all the various organocatalysts, the pyrrolidine-acetic acid catalyst is one of the most versatile for many important organic reactions and asymmetric transformations [61]. Based on this, an efficient method for the synthesis of fused pyran-pyrazole derivatives **59** was developed through a one-pot, three-component, solvent-free reaction of type 1*H*-pyrazole-4-carbaldehyde **31**, various active methylenes **58**, and malononitrile **2** under microwave irradiation in the presence of pyrrolidine-acetic acid (10 mol%) as a bifunctional catalyst, affording products **59** in good yields, as shown in Table 16 [62]. Some highlights of this protocol were associated with the solvent-free reaction conditions, shorter reaction time, greater selectivity, and straightforward workup procedure. The synthesized compounds **59** were investigated against a representative panel of six pathogenic strains using the broth microdilution MIC method for their in vitro antimicrobial activity. The investigation of the antimicrobial activity data revealed that some compounds **59** showed good to excellent antibacterial activity against the representative species when compared with the standard drugs such as ampicillin, ciprofloxacin, and norfloxacin. Particularly, compounds **59c**, **59e**, **59h**, **59m**, **59n**, **59o**, **59q**, **59r**, and **59t** were found to be the most efficient antimicrobials in the series, as shown in Table 16.

Due to the fact pyrazole and pyrimidine-2,4,6-trione heterocycles are interesting pharmacophores for pharmaceutical targets in synthetic and natural products [63], as well as being used as a precursor for the construction of condensed heterocyclic systems [64], a series of pyrazole-thiopyrimidine–trione derivatives **61** was synthesized via a one-pot multi-component reaction in aqueous media with the purpose of identifying new drugs as antibacterial agents [65]. Thus, a cascade Aldol–Michael additions of *N*,*N*-diethyl thiobarbituric acid **60**, 3-methyl-1-phenyl-1*H*-pyrazol-5(4*H*)-one **19**, and aldehydes **1** mediated by aqueous Et_2_NH as a catalyst afforded the pyrazole-thiobarbituric acid derivatives **61** in (63−88%) yield with a broad substrate scope under mild reaction conditions, as shown in Table 17.

**Figure 5:**
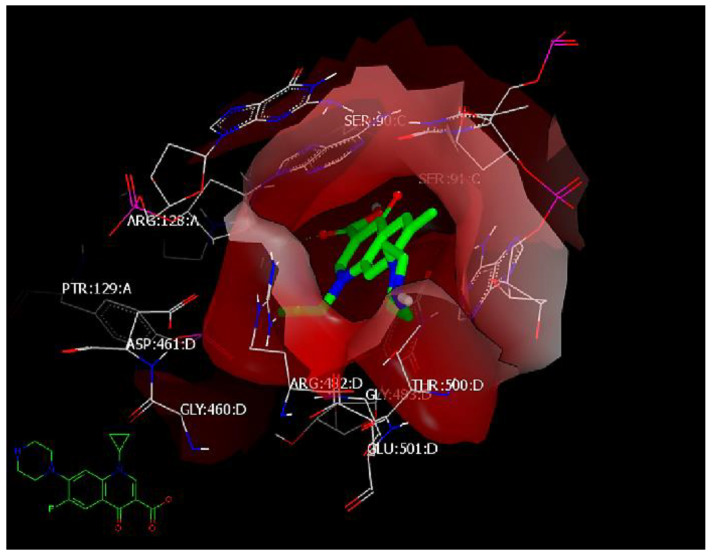
Visual representation of ciprofloxacin docked with 5BTC, showing hydrophobic–hydrophobic interaction and hydrogen bonding with ARG 128:A, as shown by Vida. Image adapted from Elshaier et al. [65].

The new compounds **61** were evaluated for their antibacterial activity against three Gram-positive bacterial strains. Compound **61c** exhibited the best activity against *Staphylococcus aureus* and *Staphylococcus faecalis* with MIC = 16 μg/L. However, compounds **61l** and **61o** were the most active against *bacillus subtilis* with MIC = 16 μg/L. Molecular docking studies for the final compounds **61** and the standard drug ciprofloxacin were performed using the OpenEye program, with different target proteins to explore their mode of action. Molecular modeling gave the comparative consensus scores of synthesized compounds **61** with two targets: DNA topoisomerase II (PDB ID 5BTC) [66] and gyrase B (PDB ID 4URM) proteins [67]. Thus, the docking with DNA Topoisomerase II (5BTC) for the standard drug ciprofloxacin had a consensus score of 1 through the hydrophobic–hydrophobic interaction and formed HB with ARG:128:A through the oxygen of its carbonyl (Figure 5).

**Figure 6:**
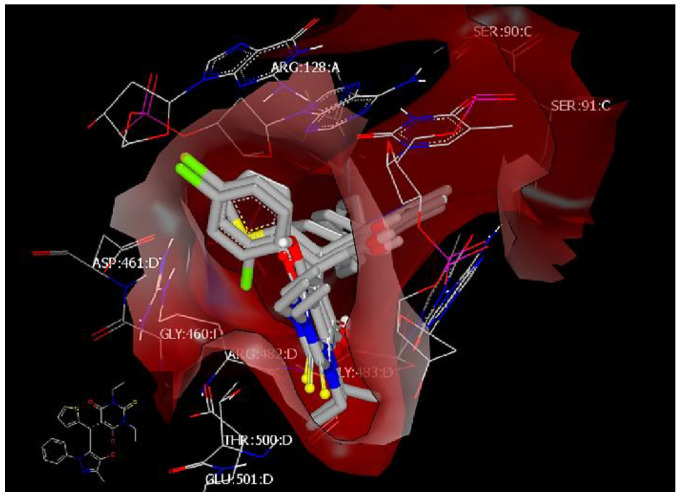
Visual representation of compounds **61c**, **61o**, and **61l** docked with 5BTC, showing no hydrogen bond interaction, as shown by Vida. Image adapted from Elshaier et al. [65].

Meanwhile, the docking mode with DNA Topoisomerase II (5BTC) for the most active compounds showed a hydrophobic–hydrophobic interaction with the receptor. Compound **61c** with a consensus score of 29, compound **61o** with a consensus score of 10, and compound **61l** with a consensus score of 54 exhibited hydrophobic–hydrophobic interactions and overlay each other (Figure 6).

**Figure 7:**
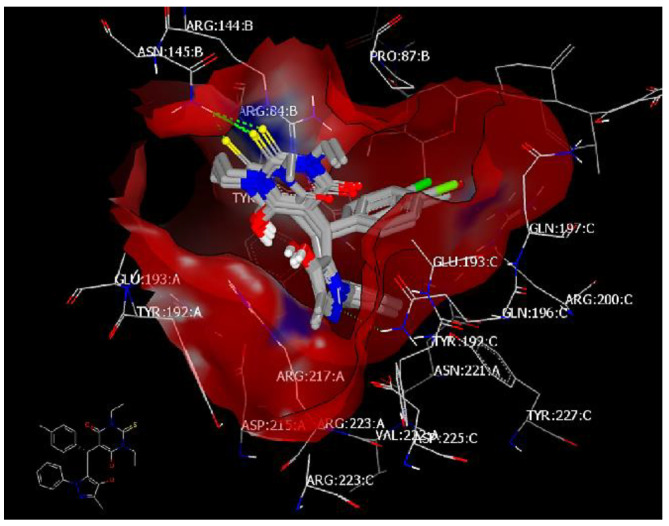
Visual representation of compound **61d** docked with 4URM and overlay with **61c**, **61a**, and **61f**. The compounds showed hydrogen bonding between the sulfur of the pyrimidine ring and ASN 145:A, as shown by Vida. Image adapted from Elshaier et al. [65].

Regarding the molecular docking with Gyrase B (PDB ID 4URM), compound **61c** (consensus score: 24) showed an HB interaction with ASN 145:A through the sulfur atom of the thiobarbiturate ring and overlay with **61a**, **61d**, and **61f** with the same HB. However, **61a** showed extra HB with GLN 196:A through the N of pyrazole (Figure 7).

Diazepine and pyrazole, which represent seven- and five-membered nitrogen-containing rings, respectively, are structures of great importance in the development of newer molecular assemblies, finding a wide range of applications in diverse fields [68,69]. In this regard, pyrazole-appended benzodiazepines were envisaged as interesting molecular templates, anticipated to have new bioprofiles [70]. Thus, several pyrazolyl-dibenzo[*b,e*][1,4]diazepinone scaffolds (**64–70**)**a**/(**71–77**)**b** were synthesized in acceptable to good yields by assembling, via a three-component approach, 5-substituted 3-methyl-1-phenyl-pyrazole-4-carbaldehydes **31** of varied natures with different cyclic diketones **20/63** and aromatic diamines **62** in the presence of indium chloride in acetonitrile at room temperature, as shown in Table 18. It is assumed that the aprotic nature of acetonitrile might have favored this reaction, as it gave poor results in protic solvents such as ethanol and water.

Structures of the obtained compounds (**64–70**)**a**/(**71–77**)**b** were confirmed by spectroscopic techniques, and based on single-crystal X-ray diffraction data of the representative compound **75b**. All obtained heterocycles (**64–70**)**a**/(**71–77**)**b** were screened, in vitro, for their antibacterial activity against three Gram-positive (*Streptococcus pneumoniae*, *Clostridium tetani*, and *Bacillus subtilis*) and three Gram-negative (*Salmonella typhi*, *Vibrio cholera*, and *Escherichia coli*) bacterial strains. Results showed that compounds **73b**, **73′b**, and **75′b** bearing a chlorophenyl-tethered pyrazolyl moiety displayed excellent to moderate inhibitory power against all six bacterial species, compared with the standard drugs gentamycin, ampicillin, chloramphenicol, ciprofloxacin, and norfloxacin, as shown in Table 18.

Due to the rapidly increasing incidence of antimicrobial resistance representing a serious problem worldwide, the development of new and different antimicrobial drugs has been a very important objective for many research programs directed toward the design of new antimicrobial agents [71]. For this purpose, a series of pyrazole-integrated thiazolo[2,3-*b*]dihydropyrimidinone derivatives **80** were synthesized via the MCR approach in a single framework as potential antimicrobial agents [72]. In this protocol, the highly activated intermediates **31** and **78** were reacted with monochloroacetic acid **79** and anhydrous sodium acetate in an acetic acid-acetic anhydride medium, resulting in the formation of the target compounds **80** in acceptable to good yields, as shown in Table 19. The synthesized compounds **80** were evaluated for their in vitro antibacterial activity against a Gram-positive organism (*Staphylococcus aureus*), Gram-negative organisms (*Klebsiella pneumoniae*, *Pseudomonas aeruginosa*, and *Escherichia coli*), and compared with that of the standard drug streptomycin. The zone of inhibition was determined by the agar well diffusion method. Remarkably, as observed in the antibacterial activity described in Table 19, compounds **80a**, **80b**, and **80d** showed good activity for all the tested species that contained electron-withdrawing 3,4-dichloro phenyl groups on the pyrazole ring, indicating that such a substitution is a favorable site for high activity in future developments.

Considering that molecular hybridization using a multicomponent approach is an effective strategy for the synthesis of new bioactive compounds and is being used in modern medicinal chemistry [73,74], a series of heterocyclic compounds containing pyrazolo[3,4-*b*]pyridin-6(7*H*)-one linked 1,2,3-triazoles **84** were synthesized to develop new pharmacophores with promising biological activities [75]. In this approach, the novel molecular hybrids **84** containing pyrazole, pyridinone, and 1,2,3-triazole were obtained from a one-pot four-component reaction of Meldrum’s acid **81**, substituted aryl azides **82**, 4-(prop-2-yn-1-yloxy)aryl aldehydes **1**, and 3-methyl-1-phenyl-1*H*-pyrazol-5-amine **83** using *L*-proline as a basic organocatalyst, aq. solution of CuSO_4_.5H_2_O and aq. solution of sodium ascorbate as catalysts at 100 °C through click chemistry in PEG-400 as a highly efficient and green media. Thus, a diverse library of 4-(4-((1-(aryl)-1*H*-1,2,3-triazol-4-yl)aryl)-3-methyl-1-phenyl-4,5-dihydro-1*H*-pyrazolo[3,4-*b*]pyridin-6(7*H*)-ones **84** was obtained in high yields, as shown in Table 20.

The structure of the synthesized compounds **84** was confirmed by the single-crystal X-ray diffraction analysis for compound **84e**. The in vitro antibacterial activity of all obtained compounds **84** was evaluated against six antibacterial strains. Four Gram-positive bacteria (*Staphylococcus aureus*, *Staphylococcus epidermidis*, *Bacillus subtilis*, and *Bacillus cereus*) and two Gram-negative bacteria (*Escherichia coli* and *Pseudomonas aeruginosa*) were used in this study. The antibacterial activity of all compounds **84** was evaluated by the agar well diffusion method using ciprofloxacin as a standard. The results of the antibacterial activity evaluation revealed that all compounds possessed good activity against Gram-positive bacterial strains and no activity against Gram-negative bacterial strains, that is, *Escherichia coli* and *Pseudomonas aeruginosa*, as shown in Table 20. Particularly, results showed that compound **84k** displayed the best antibacterial activity with a diameter of growth of the inhibition zone of 21.6 mm against *Staphylococcus aureus*, 22.6 mm against *Staphylococcus epidermidis*, 22.3 mm against *Bacillus subtilis*, and 23.6 mm against *Bacillus cereus* bacteria. The structure–activity relationship study of these compounds revealed that compounds **84e, 84k**, having a methyl group on phenyl group attached to the triazole ring in the molecule, showed better activity compared to other compounds.

It is well known that ionic liquids have become excellent alternatives to organic solvents and catalysts for a large array of organic reactions, due to their favorable properties [76]. To expand their previous application of the SO_3_H-functional Brønsted-acidic halogen-free ionic liquid 1,2-dimethyl-*N*-butanesulfonic acid imidazolium hydrogen sulfate ([DMBSI]HSO_4_) in the synthesis of heterocyclic compounds [77], Mamaghani, et al. reported a rapid, straightforward, and highly efficient one-pot synthesis of pyrano[2,3-*c*]pyrazole derivatives **86** and spiro-conjugated pyrano[2,3-*c*]pyrazoles **87** in the presence of [DMBSI]HSO_4_ as a catalyst via a one-pot four-component reaction under solvent-free conditions [78]. In this approach, the reaction of *β*-ketoesters **3/17**, hydrazine hydrate **4**, malononitrile **50** (R^2^ = CN), and aromatic aldehydes **1** in the presence of [DMBSI]HSO_4_ in solvent-free conditions at 60 °C afforded pyrano-pyrazole derivatives **86** in good to excellent yields (Table 21). Additionally, the reaction of equimolar amounts of *β*-ketoesters **3/17**, hydrazine hydrate **4**, alkyl cyanides **50**, and 1,2-diketones **85a,b** under the aforementioned optimized solvent-free conditions afforded the spiro-conjugated pyrano-pyrazoles **87** in high yields (85–96%), as shown in Table 22.

The antibacterial activity of some of the synthesized compounds **86a–o** and **87a–g** was examined against both Gram-negative (*Pseudomonas aeruginosa* and *Escherichia coli*) and Gram-positive (*Micrococcus luteus* and *Bacillus subtilis*) bacteria, using tetracycline and erythromycin as standard drugs. The results revealed that most of the compounds (**86a–o** and **87a–g**) exhibited moderate activity toward *Micrococcus luteus* as revealed by the diameters of their inhibition zones. In addition, the results also showed that most of these compounds were not active against *Escherichia coli* and *Pseudomonas aeruginosa*, as shown in Table 21 and Table 22.

It is well known that eco-friendly reaction paths, environmentally friendly catalysts, solvents, and prepared novel biologically active heterocycles have been becoming imperative key points for several unending investigation programs [79,80]. In this way, the development of a method for the synthesis of thioether-linked pyranopyrazoles **88** was performed via a reusable green catalyst, green solvent, and multicomponent domino approach [81]. Thus, the five-component reaction between the commercially available type 5-phenyl-1,3,4-oxadiazole-2-thiol **11**, aromatic aldehydes **1**, type phenyl hydrazine **15**, ethyl 4-chloro-3-oxobutanoate **8**, and malononitrile **2** in an ethanol–water solvent mixture at 70 °C using the Montmorillonite K-10 clay catalyst afforded products **88** in the range of 81–89% yield, as shown in Table 23. In this way, the K-10 catalyst behaved as a key point to promote this scientific path. Because of its acid catalytic nature to rapidly initiate the reactions, reusability, simple work-up process, time minimization, inexpensive nature, natural solvent compatibility, suppression of the side products leading to its cost reduction and eco-friendliness, the use of the K-10 catalyst highly improved this protocol compared to its catalyst-free analog procedure. Subsequently, the obtained compounds **88** were tested for their antibacterial properties against Gram-positive B*acillus subtilis* and *Staphylococcus aureus* and Gram-negative *Escherichia coli* and *Pseudomonas aeruginosa* human pathogens under the disc diffusion method, using tetracycline as a drug base, as shown in Table 23. The outcomes organized in Table 23 indicate that compound **88e** revealed outstanding antibacterial inhibition compared to all other active compounds against the four bacteria. In addition, it showed noticeable activity towards *Escherichia coli* and *Pseudomonas aeruginosa* pathogens and inhibition zone values more than the reference drug tetracycline. Moreover, compound **88n** also exhibited very good inhibition zone values, indicating that these two active compounds may be used to support further investigation as a way to ascertain new antibacterial agents.

Similarly to that previously described [78], Ambethkar, et al. reported an efficient grinding protocol for the synthesis of dihydropyrano[2,3-*c*]pyrazole derivatives **90**, in excellent yields, from a four-component reaction between acetylene ester **89**, hydrazine hydrate **4**, aryl aldehydes **1**, and malononitrile **2** in the presence of *L*-proline under solvent-free conditions, as shown in Table 24 [82]. The reaction tolerated various electron-withdrawing and electron-donating substituents in the *ortho*, *meta*, and *para* positions on the ring of the corresponding aromatic aldehydes **1**, as well as heteroaromatic aldehydes. The structures of the synthesized compounds **90** were deduced by spectroscopic techniques and confirmed by X-ray crystallography for compound **90h**. Compounds **90** were further evaluated for their in vitro antibacterial activities against four bacterial strains (*Staphylococcus albus*, *Streptococcus pyogenes*, *Klebsiella pneumoniae*, and *Pseudomonas aeruginosa*), using amikacin as the standard drug. This study was carried out by the agar well diffusion method using DMSO as a negative control. The antimicrobial data revealed that the compounds **90a, 90g, 90h, 90i, 90j, 90k**, and **90l** showed activity against the four bacterial strains evaluated, as shown in Table 24.

Propyl sulfonic acid-functionalized SBA-15 as a heterogeneous Brønsted acid, with its hexagonal structure, high surface area, and large pore size, exhibits efficient catalytic activity in a variety of organic reactions [83]. In this regard, the design and optimization of a convenient three-component approach for the synthesis of a series of tricyclic fused pyrazolopyranopyrimidine derivatives **91** using SBA-Pr-SO_3_H as a nanocatalyst was performed [84]. The morphology of the SBA-Pr-SO_3_H catalyst was verified by SEM and TEM images. The results confirmed that the hexagonally ordered mesoporous structure of SBA-15 silica was well retained after the chemical grafting reaction. In this approach, the one-pot three-component reaction of barbituric acids **60**, aromatic aldehydes **1**, and type 3-methyl-5-pyrazolone **19** under reflux conditions in water and the presence of a catalytic amount of SBA-Pr-SO_3_H afforded the target products **91** in 89–96%, as shown in Table 25. The mild reaction conditions, reusability, both electron-rich and electron-deficient aldehydes tolerance, high product yields, short reaction times, and simple work-up procedures were some advantages of this method.

The compounds **91** were screened in vitro using the disc diffusion method (IZ). The microorganisms used were *Pseudomonas aeruginosa* and *Escherichia coli* as Gram-negative bacteria, and *Staphylococcus aureus* and *Bacillus subtilis* as Gram-positive bacteria. The activities of each compound were compared with chloramphenicol and gentamicin as references. The inhibition zones of compounds **91** around the discs are shown in Table 25. All compounds **91** exhibited significant antibacterial activities against *Staphylococcus aureus* when compared with the reference drugs. All compounds **91** were able to inhibit the growth of *Bacillus subtilis* and *Escherichia coli*. No compound showed antibiotic activity against *Pseudomonas aeruginosa*.

Coumarin analogs are a group of privileged bioactive oxygen heterocycles, found substantially in nature with a wide range of structural modifications [85,86]. Knowing the synthetic and biological value of coumarin, but also pyrazole and pyran scaffolds, an investigation directed toward hybridization of these three pharmacophoric motifs in a single molecule was performed. In this sense, the synthesis, characterization, and biological studies of a series of coumarin-based pyrano[2,3-*c*]pyrazoles **93**, pyrazolylpropanoic acids **94**, and dihydropyrano[2,3-*c*]pyrazol-6(1*H*)-ones **95** using conventional methods was established [87]. Products **93** were obtained through a base-catalyzed one-pot four-component approach when a mixture of hydrazine hydrate **4**, ethyl acetoacetate **3**, formylcoumarin **92**, and type nitrile **50** in the presence of DMAP as a base was vigorously stirred at room temperature under an open atmosphere. The corresponding pyrano[2,3-*c*]pyrazole-5-carbonitriles **93** were obtained in a range of 86–94%. Subsequently, the treatment of compounds **93** with formic acid under reflux afforded the pyrazolylpropanoic acids **94** as pure white products in 77–89%. Finally, the intramolecular cyclization of propanoic acids **94** with thionyl chloride in ACN at reflux generated the tail-tail pyranone (pyranone-4-pyranone or C_4_-C_4_ pyranone) derivatives **95** in 81–85%, as shown in Table 26. The structures of the synthesized compounds were deduced by spectroscopic techniques and particularly confirmed by X-ray crystallography for compound **93b**.

The synthesized compounds **93**, **94**, and **95** were also screened for their antibacterial activity using ciprofloxacin as a standard drug. The susceptibility of the test organisms to synthetic compounds was assessed using a broth dilution assay as the minimum inhibitory concentration (MIC) and four microorganisms for the study: Two Gram-positive (*Staphylococcus aureus* and *Staphylococcus faecalis*) and two Gram-negative (*Escherichia coli* and *Pseudomonas aeruginosa*) strains. The study revealed that almost all tested compounds showed excellent antibacterial activity against Gram-positive (*Staphylococcus aureus* and *Staphylococcus faecalis*) bacterial strains. However, in the case of Gram-negative (*Escherichia coli* and *Pseudomonas aeruginosa*) bacterial strains, only some of the synthesized compounds showed selective antibacterial activity, as shown in Table 26. Among all these synthesized scaffolds, compounds **93g** and **94b** were highly active and more potent than the remaining derivatives in both biological, as well as molecular docking simulation, studies performed with *Staphylococcus aureus* dihydropteroate synthetase (DHPS).

### 2.2. Anticancer Activity

Analysis of the database of U.S. FDA-approved drugs reveals that approximately 60% of unique small-molecule drugs contain an aza-heterocycle [88]. Particularly, aza-heterocycles play an important role in the development of clinically viable anticancer drugs [89,90]. As a result, innumerable synthetic approaches for preparing diversely functionalized aza-heterocycles with anticancer properties have been successfully described during the last decade [91,92,93,94,95]. In this way, the coumarin-containing thiazolyl-3-aryl-pyrazole-4-carbaldehydes **25a–o**, obtained via the three-component synthetic approach discussed previously in Section 2.1. Antibacterial activity (Table 5) [32], were also evaluated for their in vitro cytotoxic activity against three human cancer cell lines (DU-145, MCF-7, and HeLa) at three different concentrations (2.5, 5.0, and 100 μM) by adopting the MTT assay method in the presence of Doxorubicin as a standard reference drug. As shown in Table 27, the synthesized compounds showed moderate to good anticancer activity with IC_50_ values ranging from 5.75 μM to 100 μM. In most cases, the compounds exhibited greater cytotoxic activity on the HeLa cell line. In particular, the compounds **25m** and **25n** showed excellent cytotoxic activity against the HeLa cell line with IC_50_ values of 5.75 μM and 6.25 μM, respectively, when compared to Doxorubicin (3.92 μM). Afterward, molecular docking studies were performed to validate in vitro results and elucidate the importance of different types of interactions to inhibit the function of the probable target human microsomal cytochrome P450 2A6 (1z11.pdb) enzyme. Overall, the compounds **25m** and **25n** showed the lowest binding energy with −11.72 kcal/mol and −11.95 kcal/mol, respectively. Among the most key interacting residues, Ile366 and Cys439 actively participate in the formation of hydrogen bonding with the compounds **25m** and **25n**.

In addition, Sharma et al. developed a time-efficient and simple synthesis of pyranopyrazole derivatives **96** in 77–94% yields through a four-component reaction of (hetero)aromatic aldehydes **1**, malononitrile **2**, hydrazine hydrate **4**, ethyl acetoacetate **3**, and triethylamine in EtOH at an ambient temperature for 1–2 h (Table 28) [96]. Alternatively, the same reaction was conducted under microwave irradiation at 60 °C for 3–5 min to afford the expected products in 70–87% yields. Later, the pyranopyrazole derivatives **96** were evaluated for their in vitro anticancer activity against the Hep3B Hepatocellular carcinoma cell line. As shown in Table 28, the synthesized compounds showed anticancer activity with IC_50_ values ranging from 10 μg/mL to 128 μg/mL. Overall, the presence of certain heteroatom substituents at the 3-position of the pharmacophore may be crucial to enhancing the anticancer activity.

The 1*H*-1,2,3-triazole tethered pyrazolo[3,4-*b*]pyridin-6(7*H*)-ones **84a–l**, obtained via the multicomponent synthetic approach discussed previously in Section 2.1. Antibacterial activity, was also subjected to apoptosis studies on ovarian follicles of goats (Capra hircus) (Table 20) [75]. In summary, all compounds **84a–l** caused cellular degeneration and induced apoptosis within the granulosa cells at a 10 μM dose concentration and 6 h exposure duration with tje percentage of apoptosis ranging from 22.15% to 41.35% in comparison with the control (9.21%) (Table 20). In particular, the compounds **84b** (R = H, Ar = 3-Cl-4-FC_6_H_3_), **84e** (R = H, Ar = 4-MeC_6_H_4_), and **84l** (R = MeO, Ar = 3-ClC_6_H_4_) displayed the maximum incidence of apoptotic attributes within granulosa cells (37.50%, 36.08%, and 41.35%, respectively). To assess the DNA fragmentation within granulosa cells, an important hallmark of apoptosis, a TUNEL assay was performed using the DAB stain. Overall, the maximum incidence of DNA fragmentation was observed after treatment with compounds **84b**, **84e**, and **84l**.

In 2017, Salama and collaborators designed the synthesis of *bis*-1,4-dihydropyrano[2,3-*c*]pyrazole-5-carbonitrile derivatives **97** in high yields by a pseudo-five-component reaction of *bis*-aldehydes 1*, malononitrile **2**, and pyrazolone **19** catalyzed by piperidine in refluxing ethanol for 3 h (Table 29) [97]. All synthesized compounds **97a–f** were evaluated for their in vitro anticancer activity against *Lung* A549, *Breast* MCF-7, and *Liver* HepG2 cell lines with IC_50_ values ranging from 0.014 mM to 3.34 mM. Overall, the compound **97e** gave the highest cytotoxic values against the three selected lines of A549, MCF-7, and HEPG2 with IC_50_ values of 0.37, 0.31, and 0.014 mM, respectively. Later, a molecular docking simulation was performed to investigate the interactions of the compound **97e** with vascular endothelial growth factor receptor 2 (VEGFRTK) (PDB code: 3wze). It forms two hydrogen bonds with Arg1066 through an amino group and His816 through a cyano group. Furthermore, the compound **97e** forms three *π*–cation interactions between Arg1027 and the pyrazole ring, as well as His816 and Arg1027 with the 4-aryl group. Additionally, the compound **97e** showed a higher potent binding mode than the standard inhibitor Sorafenib.

**Scheme 5:**
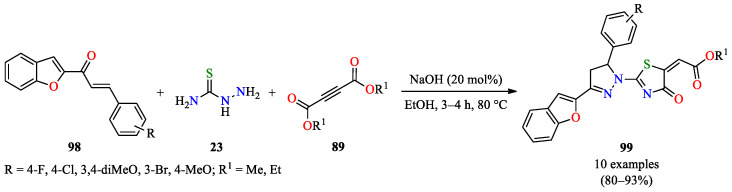
Three-component synthesis of pyrazolo-oxothiazolidine derivatives **99** as antiproliferative agents.

Some groups are interested in the use of *α*,*β*-unsaturated ketones to access functionalized pyrazoline derivatives. For instance, Yakaiah and colleagues described the synthesis of pyrazolo-oxothiazolidine derivatives **99** with 80–93% yields through a three-component reaction of benzofuran-based chalcones **98**, thiosemicarbazide **23**, and dialkyl acetylenedicarboxylates **89** catalyzed by NaOH (20 mol%) in ethanol at 80 °C (Scheme 5) [98]. The synthesized compounds were evaluated for their antiproliferative activity against the A549 *Lung* cancer cell line with IC_50_ values ranging from 0.81 μg/mL to > 5 μg/mL. Especially, the compound **99f** (R = 4-F, R^1^ = Me, IC_50_ = 0.81 μg/mL) showed higher activity than the standard drug sorafenib (IC_50_ = 3.78 μg/mL). Molecular docking studies indicated that compound **99f** had the greatest affinity for the catalytic site of the receptor VEGFR2 (PDB ID code: 4AGD and 4ASD). The binding mode of compound **99f** with the active site of VEGFR2 (PDB ID code: 4AGD) showed one hydrogen bond between the oxothiazolidine-containing carbonyl group and CYS919. In the case of VEGFR2 (PDB ID code: 4ASD), the oxygen atom of the benzofuran ring and carbonyl group formed two hydrogen bonds with residues ASP1046 and ARG1027, respectively.

**Scheme 6:**
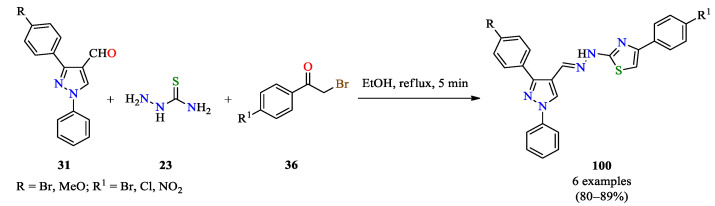
Three-component synthesis of (*E*)-2-(2-((3-aryl-1-phenyl-1*H*-pyrazol-4-yl)methylene)hydrazinyl)-4-arylthiazoles **100** as apoptosis inducers.

The multicomponent synthesis of highly functionalized *N*-heterocycles as apoptosis inducers has been successfully implemented. For instance, a mixture of 3-aryl-1-phenyl-1*H*-pyrazole-4-carbaldehydes **31**, thiosemicarbazide **23**, and *α*-bromoacetophenones **36** in refluxing ethanol for 5 min afforded a series of (*E*)-2-(2-((3-aryl-1-phenyl-1*H*-pyrazol-4-yl)methylene)hydrazinyl)-4-arylthiazoles **100** (Scheme 6) [99]. The solids were filtered and recrystallized from ethanol to afford pyrazole derivatives with high yields and short reaction times. The percentage of apoptosis was investigated in granulosa cells of ovarian antral follicles after treatment with compounds at a 10 μM concentration for a 6 h exposure duration. The compounds **100b** (R = Br, R^1^ = Cl) and **100e** (R = MeO, R^1^ = Cl) showed the maximum potency to induce apoptosis with percentages of apoptosis of 23.45% and 25.61%, respectively, in comparison with the control (5.14%). Moreover, the microphotograph of granulosa cells with a TUNEL assay revealed that all compounds induced DNA fragmentation. As expected, the compounds **100b** and **100e** induced the maximum DNA damage, indicating apoptotic cells with fragmented DNA.

**Scheme 7:**
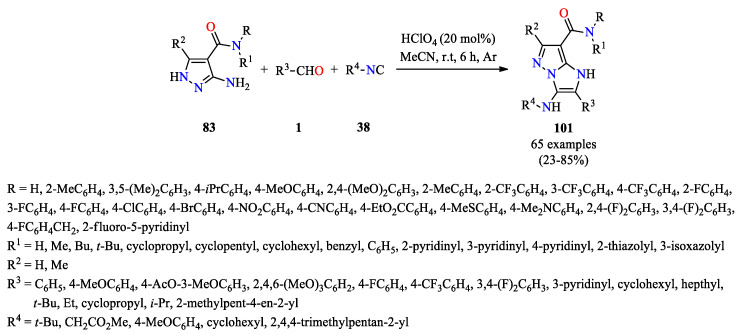
Three-component synthesis and anticancer activity of imidazo[1,2-*b*]pyrazole-7-carboxamide derivatives **101**.

Remarkably, the Groebke–Blackburn–Bienaymé reaction has been used to efficiently prepare diverse azole-fused imidazoles of biological interest. For instance, a series of imidazo[1,2-*b*]pyrazoles **101** were prepared in moderate to high yields through a GBB-type three-component reaction of 3-amino-1*H*-pyrazole-4-carboxamides **83**, aldehydes **1**, and isocyanides **38** catalyzed by HClO_4_ (20 mol%) in acetonitrile at an ambient temperature for 6 h (Scheme 7) [100]. Since an oxidative minor side reaction yielding dehydrogenated 3-imino derivatives of the target products was observed, an argon atmosphere was employed. The antitumor activity of all imidazo[1,2-*b*]pyrazole-7-carboxamides **101** was evaluated against two human (*Acute promyelocytic leukemia* HL-60 and *Breast* adenocarcinoma MCF-7) and one murine (Mammary carcinoma 4T1) cancer cell lines using doxorubicin as a positive control. Among 27 primary carboxamides, the compound **101a** (R = R^1^ = R^2^ = H, R^3^ = *t*-Bu, R^4^ = 2,4,4-trimethylpentan-2-yl) showed the most significant cytotoxic activity against HL-60, MCF-7, and 4T1 cell lines with IC_50_ values of 1.24, 1.49, and 1.88 μM, respectively. From 38 secondary and tertiary carboxamides, the compound **101b** (R = H, R^1^ = 4-FC_6_H_4_, R^2^ = H, R^3^ = *t*-Bu, R^4^ = 2,4,4-trimethylpentan-2-yl) displayed the highest potency against HL-60, MCF-7, and 4T1 cell lines with IC_50_ values ranging from 0.183 μM to 7.43 μM. Finally, the annexin V PI assay revealed that the most potent derivatives **101a** and **101b** induced apoptosis in HL-60 cells.

In 2019, Ansari and colleagues developed a Pd-catalyzed one-pot four-component protocol for the synthesis of highly functionalized pyrazolo[1,5-*c*]quinazolines **104**/**105** (Table 30) [101]. Initially, a mixture of type 2-azidobenzaldehyde **1**, isocyanide **38**, and tosyl hydrazide **103** in the presence of palladium acetate (7.5 mol%) as a catalyst in toluene was stirred at an ambient temperature for 15 min to generate azomethine imine in situ, then the acetonitrile derivative **50** and DABCO were added, and the reaction was stirred at 100 °C for 2 h to afford pyrazolo[1,5-*c*]quinazolines **104**. For aroylacetonitrile **102**, a similar methodology was developed for the synthesis of target compounds **105** with the addition of iodine (10 mol%) as a catalyst. The presence of electron-withdrawing groups on the *α*-position of acetonitrile such as CN, COOR^2^, and COR^2^ was essential for their participation in the reaction. For instance, compound **104d** was not formed due to the absence of such groups. The reaction proceeds with good functional group tolerance, excellent regioselectivity, a high atom economy, and low catalyst loading under simple reaction conditions. Target compounds **104** and **105** were screened for their antiproliferative potential against MDA-MB-231, A549, and H1299 cell lines using erlotinib and gefitinib as standard drugs. Notably, compound **105b** showed the most significant cytotoxic activity against MDA-MB-231, A549, and H1299 cell lines with IC_50_ values of 1.93, 1.06, and 1.32 μM, respectively, when compared to erlotinib and gefitinib. Next, the inhibition of **105b** in comparison to erlotinib toward the ATP-dependent phosphorylation of EGFR was investigated at concentrations of 100, 250, and 500 nM. The results suggested that compound **105b** possesses the most potent EGFR inhibition with an IC_50_ value of 157.63 nM, in comparison to erlotinib (IC_50_ = 201.34 nM). Additionally, compound **105b** elevated ROS levels and altered the mitochondrial potential, resulting in apoptosis via the G1 phase.

Furthermore, the molecular docking studies of the most potent EGFR inhibitor **105b** within the active site of the EGFR protein (PDB ID: 1M17) showed that the compound perfectly fits into the ATP domain of EGFR and has a much better docking score (−9.10 kcal/mol) than erlotinib (−7.20 kcal/mol); thus, revealing that smaller and polar substitutions on the pyrazole ring are essential for binding with EGFR.

**Scheme 8:**
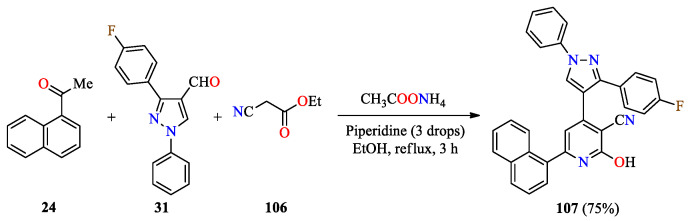
Four-component synthesis of 4-(3-(4-fluorophenyl)-1-phenyl-1*H*-pyrazol-4-yl)-2-hydroxy-6-(naphthalen-1-yl)nicotinonitrile **107** with anticancer activity.

Recently, the piperidine-catalyzed four-component reaction of 1-(naphthalen-1-yl)ethanone **24**, 3-(4-fluorophenyl)-1-phenyl-1*H*-pyrazole-4-carbaldehyde **31**, ethyl 2-cyanoacetate **106**, and ammonium acetate in refluxing ethanol for 3 h was reported for the regioselective synthesis of 4-(3-(4-fluorophenyl)-1-phenyl-1*H*-pyrazol-4-yl)-2-hydroxy-6-(naphthalen-1-yl)nicotinonitrile **107** in 75% yields (Scheme 8) [102]. Although the scope of the reaction was not further studied, compound **107** was employed in the construction of an important library of diversely functionalized *N*-heterocycles containing pyridine and pyrazole moieties without using a multicomponent approach. Next, the anticancer activity of compound **107** was screened against HepG2 and HeLa cell lines using a standard MTT assay in the presence of doxorubicin as a standard drug. Thus, compound **107** showed a low cytotoxic effect with IC_50_ values of 20.00 μM and 35.58 μM for HepG2 and HeLa cell lines, respectively, when compared to doxorubicin (IC_50_ = 4.50 μM and 5.57 μM, respectively).

Importantly, a green and efficient synthesis of 4,5-dihydropyrano[2,3-*c*]pyrazol-6(2*H*)-one derivatives **108** is described by the four-component reaction of aromatic aldehydes **1**, Meldrum’s acid **81**, methyl acetoacetate **3**, and hydrazine hydrate **4** catalyzed by potassium carbonate (10 mol%) in water–ethanol (5.0 mL, 1:1) at reflux for 2–4 h (Table 31) [103]. This protocol provides several advantages such as environmental friendliness, short reaction times, good yields (59–85%), and a simple workup procedure. The cytotoxic activity of synthesized compounds **108a–l** was evaluated by MTT assay on A2780, MCF-7, and PC-3 cell lines using doxorubicin as a standard drug. The compounds **108g** in the A2780 cell line (IC_50_ = 104 μM), **108g** and **108i** in the MCF-7 cell line (IC_50_ = 87 and 23 μM, respectively), and **108g–i** in the PC-3 cell line (IC_50_ = 60, 50, and 31 μM, respectively) showed the best results close to the control drug doxorubicin (Table 31). Therefore, the compounds **108g** and **108h** were adopted for the identification of mechanisms of action on A2780 and MCF-7 cell lines. In summary, the compound **108h** increased caspase-3 and caspase-9 activation in the A2780 cell line, while the compound **108g** significantly increased caspase-9 activation in the MCF-7 cell line.

Additional to the antibacterial activity previously discussed in Table 10 (Section 2.1. Antibacterial activity) for the spiroindenopyridazine-4*H*-pyrans **45** obtained via a four-component synthesis [43], their cytotoxic activity on non-small cell *lung* cancer (A549), *Breast* cancer (MCF-7), Human malignant *melanoma* (A375), *Prostate* cancer (PC-3 and LNCaP), and normal cells HDF (human dermal fibroblast) were also investigated using the MTT colorimetric assay in the presence of etoposide as a positive control. As shown in Table 32, the compounds have no inhibition effect on two cancer cell lines (MCF-7 and PC-3) and Normal cells HDF. Moreover, the compound **45a** displayed the highest cytotoxicity against A549, A375, and LNCaP cell lines with IC_50_ values of 40, 70.7, and 32.1 μM, respectively, when compared to etoposide (IC_50_ = 60, 25.3, and 90 μM, respectively). Interestingly, inverted fluorescent microscopy images showed that compound **45a** induced cell death in A549 cells. Treatment with **45a** leads to both the up-regulated expression of Bax and the down-regulated expression of Bcl-2 in A549 cells, confirming mitochondria-mediated apoptosis.

**Scheme 9:**
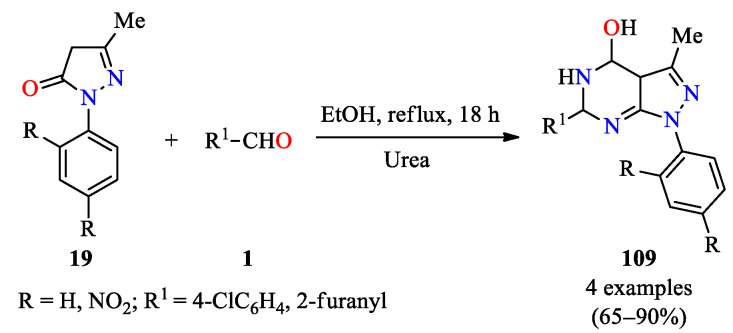
Three-component synthesis of pyrazolo[3,4-*d*]pyrimidin-4-ol derivatives **109** with anticancer activity.

In 2020, Alharthy reported the synthesis of pyrazolo[3,4-*d*]pyrimidin-4-ol derivatives **109** via a three-component reaction of 1-aryl-3-methyl-1*H*-pyrazol-5-ones **19**, urea, and (hetero)aromatic aldehydes **1** in refluxing EtOH for 18 h (Scheme 9) [104]. The reaction mixture was filtered, dried, and recrystallized from ethanol to afford compounds in 65–90% yields. The cytotoxic activity of all synthesized compounds was evaluated against MCF-7 (*Breast* cancer) and A549 (*Lung* cancer) cell lines using doxorubicin as a standard drug. Overall, the compound **109a** (R = H, R^1^ = 4-ClC_6_H_4_) displayed better inhibitory activity against MCF-7 and A549 cell lines with IC_50_ values of 74 μM and 11.5 μM, respectively, when compared to doxorubicin (IC_50_ = 35.2 μM and 9.80 μM, respectively).

**Scheme 10:**
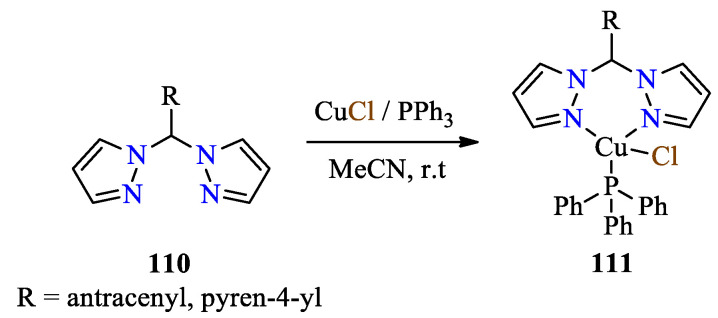
Three-component synthesis of copper(I) complexes **111** with pyrazole-linked triphenylphosphine moieties as mitochondria- and nucleolus-labelling probes.

Considerable interest in pyrazole-containing copper(I) complexes have been stimulated by promising pharmacological applications, fluorescence sensing, and catalytic properties [105,106]. For instance, the copper(I) complexes **111** with pyrazole-linked triphenylphosphine moieties have been described as photostable and cost-effective fluorescent probes for simultaneously tracking mitochondria and nucleolus via live cell imaging techniques, in a single run and within a timeframe of just 30 min [107]. Both metallo-complexes **111** were synthesized via a three-component reaction of *bis*-pyrazole derivatives **110**, copper(I) chloride, and triphenylphosphine in HPLC-grade acetonitrile at room temperature (Scheme 10). These metallo-complexes were found to be the least cytotoxic to HeLa cells, and even at a 20 μM treatment concentration, approximately 90% of cell viability was observed in both cases. Moreover, both complexes were found to be photostable when torched with 10% of a 100 mW laser for up to 10 min.

The pyrazolyl-dibenzo[*b*,*e*][1,4]diazepinones **(64–70)a** and **(71–77)b** were obtained via a multicomponent synthetic approach and previously discussed in Section 2.1. Antibacterial activity (Table 18) [70]. These compounds were also screened for their antiproliferative potential against six human cancer cell lines using a sulforhodamine B (SRB) assay in the presence of cisplatin, etoposide, and camptothecin as standard drugs. In particular, the compounds **73b** (R = H, R^1^ = 4-ClC_6_H_4_O) and **75b** (R = H, R^1^ = 4-ClC_6_H_4_S) showed better antiproliferative activity against A549 (*Lung* cancer), HBL-100 (*Breast* cancer), HeLa (*Cervix* cancer), SW1573 (*Lung* cancer), T-47D (*Breast* cancer), and WiDr (*Colon* cancer) cell lines with GI_50_ values ranging from 2.6–5.1 μM and 1.8–7.5 μM, respectively, when compared to *cis*-platin (GI_50_ = 1.9–26 μM), etoposide (GI_50_ = 1.4–23 μM), and camptothecin (GI_50_ = 0.23–2.0 μM). Docking studies were performed for compounds **73b** and **75b** along with etoposide in the active site of human topoisomerase II alpha (PDB ID: 5GWK) [70]. The oxygen atom of the carbonyl group of guanine, a part of DNA, forms a hydrogen bond with the NH group of the diazepine ring of the compound **73b**. However, compound **75b** did not form any hydrogen bond with the DNA. Etoposide showed a docking score of −3.59, which is better than compounds **73b** (−1.37) and **75b** (−0.95). In addition, etoposide displayed lower binding energy (−70.81 kcal/mol) than compounds **73b** and **75b** (−31.78 kcal/mol and −25.97 kcal/mol, respectively).

**Scheme 11:**
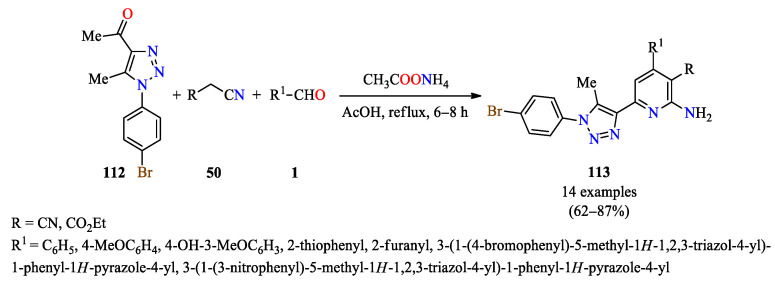
Four-component synthesis and anticancer evaluation of 1,2,3-triazolyl-pyridine hybrids **113**.

In 2021, Rashdan et al., described the synthesis of 1,2,3-triazolyl-pyridine hybrids **113** through a four-component reaction of 1,2,3-triazole derivatives **112**, active methylene compounds **50**, (hetero)aromatic aldehydes **1**, and ammonium acetate in refluxing acetic acid for 6–8 h (Scheme 11) [108]. After the completion of the reaction, the mixture was cooled and the precipitated products were filtered, washed with water, dried, and recrystallized from ethanol to give 1,2,3-triazolyl-pyridine hybrids **113** in 62–87% yields. The cytotoxic activities of all synthesized compounds were screened against HepG2 Hepatocellular carcinoma and BALB/3T3 (Murine fibroblast) cell lines using an MTT assay and the standard drug doxorubicin. Overall, the compounds **113d** (R = CN, R^1^ = 3-(1-(4-bromophenyl)-5-methyl-1*H*-1,2,3-triazol-4-yl)-1-phenyl-1*H*-pyrazole-4-yl) and **113e** (R = CN, R^1^ = 3-(1-(3-nitrophenyl)-5-methyl-1*H*-1,2,3-triazol-4-yl)-1-phenyl-1*H*-pyrazole-4-yl) showed an excellent anticancer activity against HepG2 cell line with IC_50_ values of 0.64 μg/mL and 1.08 μg/mL, respectively, in comparison to the reference drug (IC_50_ = 3.56 μg/mL). Meanwhile, they did not show toxicity on the Normal cell lines (BALAB/3T3).

**Scheme 12:**
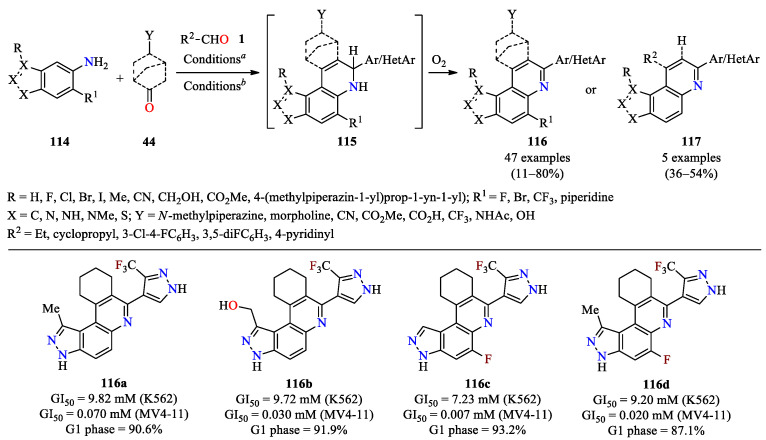
Three-component synthesis and anticancer evaluation of 3*H*-pyrazolo[4,3-*f*]quinoline derivatives **116** and **117**. Reaction conditions: (a) (i) EtOH, reflux, 2 h, and (ii) cyclic ketone **44**, catalyst HCl, reflux, 12 h; (b) (i) THF, reflux, 2 h, and (ii) acyclic ketone or acetophenone **44** and I_2_ (10 mol%), reflux, 12 h.

Very recently, novel 3*H*-pyrazolo[4,3-*f*]quinolines **116**/**117** were rapidly assembled through a one-pot Doebner/Povarov-type MCR utilizing arylamines **114** and (hetero)aromatic aldehydes **1** to form Schiff bases, which subsequently reacted with the enol form of cyclic or acyclic ketones **44** in the presence of catalytic acid to give an intermediate **115** that is readily oxidized by air to form a quinoline core (Scheme 12) [109]. Authors explored how various modifications on compounds **116**/**117** affected the proliferation of K562 (*Chronic myelogenous leukemia*, CML) and MV4–11 (*Acute myeloid leukemia*, AML) cell lines in the presence of quizartinib (GI_50_ = > 20 μM and 0.002 μM, respectively) and dinaciclib (GI_50_ = 0.013 μM and 0.007 μM, respectively) as positive controls. Overall, the compounds **116**/**117** exhibited antiproliferative activity against K562 and MV4–11 cell lines with GI_50_ values ranging from 3.28 to > 100 μM and 0.007 to > 100 μM, respectively. The most potent inhibitors **116a**, **116b**, **116c**, and **116d** caused the accumulation of >80% of treated MV4–11 cells in the G1 phase. These compounds blocked the proliferation of K562 and MV4–11 cell lines with GI_50_ values ranging from 7.23 to 9.82 μM and 7 to 70 nM, respectively (Scheme 12). Finally, molecular docking was performed between compound **116a** and active (activation loop-out, DFG-in, homology model) and inactive kinase conformation (DFG-out, PDB: 4XUF). The compound **116a** adopted a binding mode corresponding to type I FLT3 kinase inhibitors. The 3*H*-pyrazole ring formed a hydrogen bond with Cys694 in the hinge region, while the 3-trifluoromethyl moiety formed C−F···H−N and C−F···C=O interactions with Arg834 and Asp698, respectively.

**Scheme 13:**
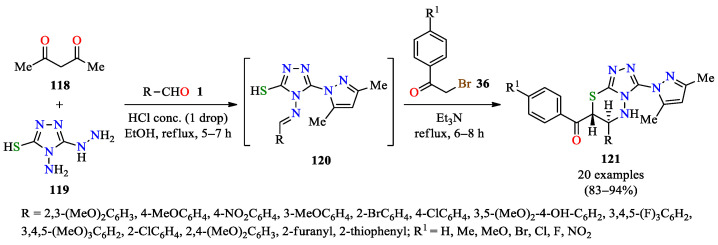
One-pot four-component synthesis and anticancer evaluation of 3-(1*H*-pyrazol-1-yl)-6,7-dihydro-5*H*-[1,2,4]triazolo[3,4-*b*][1,3,4]thiadiazine derivatives **121**.

Recently, pyrazole and dihydrothiadiazine skeletons were obtained by a one-pot four-component reaction [110]. Initially, a mixture of acetylacetone **118**, 4-amino-5-hydrazinyl-4*H*-1,2,4-triazole-3-thiol **119**, and diverse aldehydes **1** catalyzed by one drop of concentrated HCl in refluxing ethanol for 5–7 h afforded pyrazole-based intermediates **120** (Scheme 13). Then, substituted phenacyl bromides **36** and an excess of triethylamine (one drop of HCl was neutralized by one mole of Et_3_N) were added, and the resulting mixture was continued under reflux for 6–8 h. Finally, the reaction mixture was cooled to room temperature, and the formed solid was filtered and recrystallized from ethanol to afford pyrazole-based dihydrothiadiazine derivatives **121** in 83–94% yields. From the mechanistic perspective, the hydrazino functional group of compound **119** underwent cyclocondensation with acetylacetone **118** to form a pyrazole ring. Then, an appropriate amount of different aldehydes **1** and substituted phenacyl bromides **36** reacted with amine (–NH_2_) and thiol (–SH) groups mediated by HCl and Et_3_N, respectively, leading to dihydrothiadiazine derivatives **121** (Scheme 13). The synthesized compounds **121a–t** were screened for their antitumoral activity against LN-229 (*Glioblastoma*), Capan-1 (*Pancreatic adenocarcinoma*), HCT-116 (*Colorectal carcinoma*), NCI-H460 (*Lung carcinoma*), DND-41 (*Acute lymphoblastic leukemia*), HL-60 (*Acute myeloid leukemia*), K-562 (*Chronic myeloid leukemia*), and Z-138 (*Non-Hodgkin lymphoma*) cell lines using docetaxel (a microtubule depolymerization inhibitor) and staurosporine (a pan-kinase inhibitor) as positive controls. Overall, the compounds **121j** (R = 3,4,5-(F)_3_C_6_H_2_, R^1^ = Me) and **121q** (R = 2-Furanyl, R^1^ = MeO) displayed better activity against eight cancer cell lines with IC_50_ values in the range of 1.9–56.0 μM and 0.4–2.5 μM, respectively, in comparison to docetaxel (IC_50_ = 0.0009–0.0087 μM) and staurosporine (IC_50_ = 0.0004–0.0229 μM) as standard drugs. In addition, immune fluorescence analysis of tubulin in HEp-2 cells was performed with compounds **121j** and **121q** and compared to DMSO (vehicle control) and vincristine (positive control). Remarkably, these compounds inhibit the polymerization of tubulin in a dose-dependent manner.

Importantly, pyrazole-based 1,4-naphthoquinones **123** were rapidly assembled through a four-component reaction of ethylacetoacetate **3**, 2-hydroxy-1,4-naphthaquionone **122**, hydrazine derivatives **4**/**15**, and diverse aromatic aldehydes **1** catalyzed by V_2_O_5_ (5 mol%) in refluxing ethanol for 1 h (Table 33) [111]. This protocol was distinguished by its short reaction times, high yields, the use of a green solvent, and broad substrate scope. Moreover, some compounds were screened for their anticancer activity against the HeLa (*Cervical* cancer) cell line using the MTT colorimetric assay and doxorubicin as a positive control. These compounds showed good to excellent anticancer activity with IC_50_ values in the range of 2.9–25.12 μM, in comparison to doxorubicin (IC_50_ = 5.1 μM). Notably, the compounds **123i**, **123f**, and **123b** resulted in being more active than doxorubicin with IC_50_ values of 2.9, 4.36, and 4.81 μM, respectively.

**Scheme 14:**
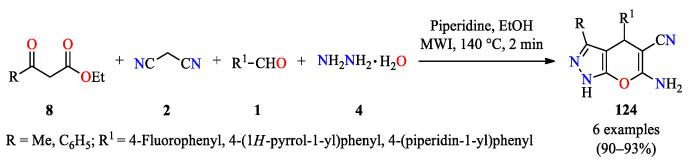
Microwave-assisted four-component synthesis of 1,4-dihydropyrano[2,3-*c*]pyrazoles **124** as anticancer agents.

Very recently, 1,4-dihydropyrano[2,3-*c*]pyrazole derivatives **124** were obtained in excellent yields through a piperidine-catalyzed four-component reaction of *β*-ketoesters **8**, malononitrile **2**, aryl/heteroaryl aldehydes **1**, and hydrazine hydrate **4** in ethanol under microwave heating at 140 °C for 2 min (Scheme 14) [112]. After completion of the reaction, the product was filtered, washed with methanol, and recrystallized from ethanol. The pyrazole derivatives **124a–f** were evaluated for their antitumor activity against four human cancer cell lines: PC-3 (*Prostate* cancer), SKOV-3 (*Ovarian* cancer), HeLa (*Cervical* cancer), and A549 (*Non-small cell lung* cancer), and two normal cell lines: Human fetal lung (HFL-1) and Human diploid fibroblasts (WI-38) using the MTT colorimetric assay in the presence of vinblastine and doxorubicin as standard drugs. These compounds displayed good cytotoxicity against PC-3, SKOV-3, HeLa, and A549 with IC_50_ values in the range of 2.0–5.5, 2.0–4.2, 1.1–3.9, and 1.1–20.9 μM, respectively, when compared to vinblastine and doxorubicin (IC_50_ = 2.7–3.1 and 1.4–2.2 μM, respectively). Overall, the compound **124c** (R = Me, R^1^ = 4-(1*H*-pyrrol-1-yl)phenyl) displayed the highest cytotoxicity against PC-3 and HeLa cell lines with IC_50_ values of 2.0 and 1.1 μM, respectively. In addition, the compounds **124a** (R = Me, R^1^ = 4-fluorophenyl) and **124e** (R = Me, R^1^ = 4-(piperidin-1-yl)phenyl) showed better cytotoxicity against SKOV-3 and A549 cell lines with IC_50_ values of 2.0 and 1.1 μM, respectively. Molecular docking studies were performed for all synthesized compounds against His-tag human thymidylate synthase (HT-hTS) in a complex with 2′-deoxyuridine 5′-monophosphate (dUMP) (PDB: 6QXH) [112]. The compounds **124a** (R = Me, R^1^ = 4-fluorophenyl), **124b** (R = C_6_H_5_, R^1^ = 4-fluorophenyl), and **124f** (R = C_6_H_5_, R^1^ = 4-(piperidin-1-yl)phenyl) stabilized in a TS binding pocket similar to dUMP through an arrangement of the pyranopyrazole cluster in perpendicular mode with Tyr270 via a hydrogen bond interaction, while the compounds **124c** (R = Me, R^1^ = 4-(1*H*-pyrrol-1-yl)phenyl) and **124d** (R = C_6_H_5_, R^1^ = 4-(1*H*-pyrrol-1-yl)phenyl) occupied the binding pocket by interaction with Asn238.

**Scheme 15:**
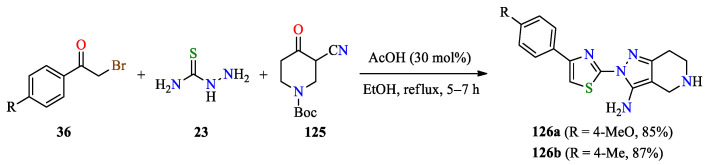
Three-component synthesis and anticancer evaluation of thiazolyl-based pyrazoles **126**.

Interestingly, thiazolyl-based pyrazoles **126** were obtained in good yields through a three-component reaction of substituted phenacyl bromides **36**, thiosemicarbazide **23**, and 1-boc-3-cyano-4-piperidone **125** catalyzed by acetic acid (30 mol%) in refluxing ethanol for 5–7 h (Scheme 15) [113]. After the completion of the reaction, the mixture was cooled to room temperature, and the precipitate was filtered, washed, and recrystallized from ethanol. The anticancer activity of synthesized compounds was screened against three human cancer cell lines: HeLa (*Cervical* cancer), A549 (*Lung* cancer), and MDA-MB-231 (*Breast* cancer) using the MTT colorimetric assay in the presence of combretastatin A-4 as a positive control. The compounds **126a** (R = 4-MeO) and **126b** (R = 4-Me) showed good cytotoxicity against HeLa, A549, and MDA-MB-231 cell lines with IC_50_ values in the range of 3.60–4.17 μM and 4.61–5.29 μM, respectively. Besides, thiazolyl-based pyrazoles and combretastatin A-4 were docked into the colchicine binding site of *β*-tubulin (PDB: 4YJ2), finding that compounds **126a** (−8.79 kcal/mol) and **126b** (−8.77 kcal/mol) have better docking scores than combretastatin A-4 (−8.45 kcal/mol). The compound **126a** interacted via two hydrogen bonds with Gln136 and Glh200.

A series of 6-amino-1,4-dihydropyrano[2,3-*c*]pyrazole-5-carbonitriles **127** were synthesized in high yields via a one-pot multicomponent approach [114]. Initially, a mixture of *β*-keto esters **8**, aryl hydrazines **15**, and zinc triflate (10 mol%) was irradiated under microwave at 80 °C for 10 min. Then, aromatic aldehydes **1** and malononitrile **2** were added to the above reaction mixture. The resulting mixture was further irradiated under microwave at 120 °C for 15 min to afford pyrazole derivatives **127a–f** under solvent-free conditions. The anticancer activity of compounds was investigated against four human cancer cell lines: 786-0 (*Renal* cancer), A431 (*Epidermal carcinoma*), MCF-7 (*Breast* cancer), and U-251 (*Human glioblastoma*) employing doxorubicin as a positive control. As shown in Table 34, the compound **127h** with –NO_2_ and –OMe groups displayed significant activity with an IC_50_ value of 9.9 μg/mL against the 786-0 cell line. Furthermore, the compounds **127b** (IC_50_ = 22.78 μg/mL), **127i** (IC_50_ = 21.98 μg/mL), and **127j** (IC_50_ = 19.98 μg/mL) with –OH or –Br groups on phenyl ring showed moderate activity against the A431 cell line, when compared to doxorubicin (IC_50_ = 1.6 μg/mL). Moreover, the compounds **127h** and **127j** showed better activity against MCF-7 and U-251 cell lines with IC_50_ values of 31.87 and 25.78 μg/mL, respectively, in comparison to doxorubicin (IC_50_ = 2.1 and 1.9 μg/mL, respectively).

**Scheme 16:**
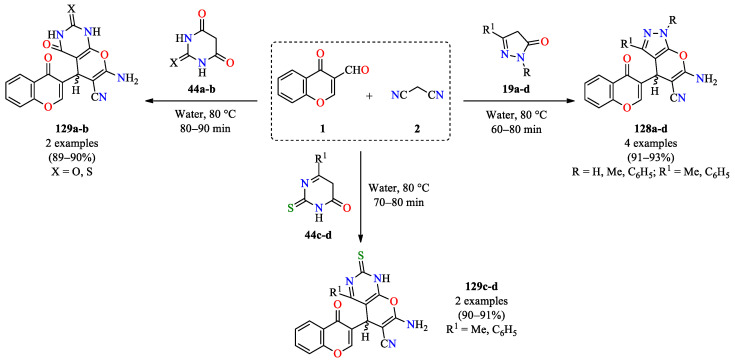
Three-component synthesis and anticancer activity of new 4-(4-oxo-4*H*-chromen-3-yl)pyrano[2,3-*c*]pyrazoles **128a−d** and 5-(4-oxo-4*H*-chromen-3-yl)pyrano[2,3-*d*]pyrimidines **129a−d**.

Ali and colleagues described a one-pot three-component reaction of 4-oxo-4*H*-chromene-3-carbaldehyde **1**, malononitrile **2**, and cyclic active methylene compounds such as pyrazolones **19a−d** and diverse pyrimidinones **44a−d** in water at 80 °C for 60−90 min to afford a series of new 4-(4-oxo-4*H*-chromen-3-yl)pyrano[2,3-*c*]pyrazoles **128a−d** and 5-(4-oxo-4*H*-chromen-3-yl)pyrano[2,3-*d*]pyrimidines **129a−d** in 91−93% and 89−91% yields, respectively (Scheme 16) [115].

The antiproliferative activity of synthesized compounds **128a−d** and **129a−d** was evaluated against three human cancer cell lines: PC-3 (*Prostate* cancer), SKOV3 (*Ovarian* cancer), and HeLa (*Cervical* cancer) using the sulforhodamine B (SRB) assay in the presence of doxorubicin as a standard drug [115]. The IC_50_ values obtained after 72 h of incubation are reported in Table 35. The compounds **128** showed antiproliferative activity against PC-3, SKOV3, and HeLa cell lines with IC_50_ values in the range of 9.7–190.3, 16.5–234.3, and 8.4–91.3 μg/mL, respectively, in comparison to doxorubicin (IC_50_ = 2.1, 2.3, and 1.9 μg/mL, respectively). In addition, compounds **129** showed antiproliferative activity against PC-3, SKOV3, and HeLa cell lines with IC_50_ values in the range of 8.9–73.4, 4.7–35.5, and 11.3–32.8 μg/mL, respectively. Particularly, compound **129a** displayed the best cytotoxic effect against PC-3, SKOV3, and HeLa cell lines with IC_50_ values of 8.9, 4.7, and 11.3 μg/mL, respectively (Table 35). Furthermore, compound **128c** showed a promising cytotoxic effect in *Cervical* cancer (HeLa) with an IC_50_ value of 8.4 μg/mL, and a moderate cytotoxicity against *Prostate* cancer (PC-3) and *Ovarian* cancer (SKOV3) with IC_50_ values of 13.2 and 16.5 μg/mL, respectively. In general, the 5-(4-oxo-4*H*-chromen-3-yl)pyrano[2,3-*d*]pyrimidines **129** resulted in being more active than 4-(4-oxo-4*H*-chromen-3-yl)pyrano[2,3-*c*]pyrazoles **128**. It should be noted that the 3-methyl-1-phenylpyrazole fragment in **128c** improved the cytotoxicity in comparison to other pyrazole derivatives. Likewise, the barbituric acid fused to the pyran core in **129a** showed higher activity than thiobarbituric acid moiety in **129b**, while the 6-phenylthiouracil moiety in **129d** displayed a better antiproliferative effect than the 6-methylthiouracil moiety in **129c**.

### 2.3. Antifungal Activity

Currently, the incidence and severity of fungal diseases have increased in patients with increased vulnerability such as neonates, burns patients, cancer patients receiving chemotherapy, patients with acquired immunodeficiency syndrome, and organ transplant patients [116]. Moreover, the use of standard antifungal therapies has been limited due to problems of toxicity, low efficacy rates, and resistance to antifungal drugs [117]. These reasons have given rise to the design and production of new chemical libraries of aza-heterocycles with distinct action or multitargeted combination therapy [116,117,118,119]. In this way, several pyrazole-containing pyrimidines **52**, 1,4-dihydropyridines **53**, and imidazoles **54** were prepared via multicomponent synthetic approaches and discussed in Section 2.1. Antibacterial activity (Table 14) [58]. Moreover, all of these compounds were screened for their antifungal activity against *Aspergillus niger* and *Aspergillus flavus* using itraconazole as a positive control. In most cases, the pyrimidines **52** showed appreciable antifungal activity against *Aspergillus niger* and *Aspergillus flavus* with MIC values of 6.25–25 μg/mL and 12.5–25 μg/mL, respectively. In particular, the compound **52f** (R = F, R^1^ = MeO) showed excellent antifungal activity against *Aspergillus niger* and *Aspergillus flavus* with MIC values of 6.25 μg/mL and 12.5 μg/mL, respectively, in comparison to itraconazole (MIC = 6.25 μg/mL for both strains). Moreover, the 1,4-dihydropyridines **53** showed some degree of inhibition for both fungal strains with MIC values ranging from 12.5 to > 100 μg/mL. However, imidazoles **54** showed that imidazole and pyrazole rings did not contribute to antifungal efficacy.

**Scheme 17:**
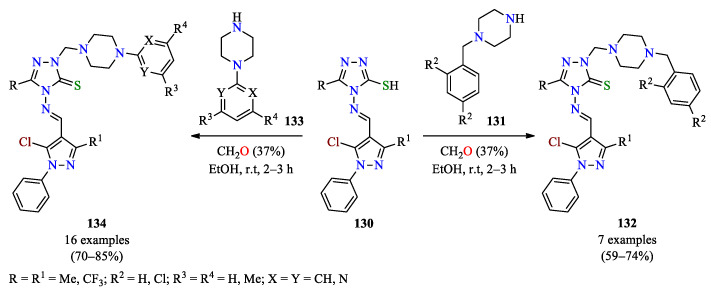
Three-component synthesis and antifungal activity of highly functionalized 1,2,4-triazole-5(4*H*)-thiones **132** and **134**.

The Mannich reaction has provided elegant and efficient solutions for the carbon–carbon and carbon–nitrogen bond formation via an iminium intermediate [120,121]. According to thione-thiol tautomerism, Wang et al. reported that the thione form undergoes a Mannich reaction via the N-H at the *α*-position of the thiocarbonyl group [121]. As a result, the Mannich reaction of 1,2,4-triazole-3-thiol forms **130**, formaldehyde solution (37 wt. % in H_2_O), and 4-(substituted benzyl)piperazines **131** in ethanol at room temperature for 2–3 h afforded 1,2,4-triazole-5(4*H*)-thiones **132** in acceptable yields (Scheme 17). The reaction was successfully extended to 4-(substituted pyrimidyl/phenyl/pyridyl)piperazines **133** under the same reaction conditions to give 1,2,4-triazole-5(4*H*)-thiones **134** in good yields. The crude reaction was placed in a refrigerator overnight, and the resulting precipitate was filtered and recrystallized from ethanol to give products. Later, the 1,2,4-triazole-5(4*H*)-thiones **132** and **134** were screened for their in vitro fungicidal activity against six plant fungal pathogens, including *Alternaria solani Sorauer*, *Gibberella sanbinetti*, *Fusarium omysporum*, *Cercospora arachidicola*, *Physalospora piricola*, and *Rhizoctonia cerealis* using triadimefon, carbendazim, and chlorothalonil as positive controls. It was found that at a 50 μg/mL concentration, most of the compounds **132** and **134** exhibited significant fungicidal activities against *Alternaria solani Sorauer*, *Physalospora piricola*, and *Rhizoctonia cerealis* with the inhibition of 26.7–47.6%, 12.5–75.0%, and 35.7–98.0%, respectively, when compared to triadimefon (31.3%, 71.4%, and 98.0%, respectively). Several compounds displayed favorable activities against other plant fungal pathogens, such as compound **134g** (R = CF_3_, R^1^ = Me, R^3^ = Me, R^4^ = H, X = Y = N) with 62.5% inhibition against *Gibberella sanbinetti*, and compounds **132g** (R = R^1^ = CF_3_, R^2^ = Cl) and **134p** (R = R^1^ = CF_3_, R^3^ = R^4^ = Me, X = Y = N) with 75.0% inhibition against *Cercospora arachidicola*, which were more effective than triadimefon (52.9% and 66.7%, respectively).

On the other hand, a series of 1*H*-1,2,3-triazole tethered pyrazolo[3,4-*b*]pyridin-6(7*H*)-ones **84a–l** was obtained via the multicomponent synthetic approach discussed in Section 2.1. Antibacterial activity (Table 20) [75]. The antifungal activity of these compounds was evaluated against *Candida albicans* and *Saccharomyces cerevisiae*. The diameter of growth of the inhibition zone (mm) and MIC value (μg/mL) of all compounds was determined using amphotericin-B as a standard drug. Overall, the pyrazolo[3,4-*b*]pyridin-6(7*H*)-ones **84a–l** showed a diameter of growth of the inhibition zone in the range of 12.3–16.3 mm and 14.3–16.6 mm for *Candida albicans* and *Saccharomyces cerevisiae*, respectively, when compared to amphotericin-B (17.6 mm and 18.3 mm, respectively). Moreover, the compounds **84a–l** showed MIC values in the range of 64–256 μg/mL for both *Candida albicans* and *Saccharomyces cerevisiae*, when compared to amphotericin-B (100 μg/mL for both cases). Interestingly, the compound **84k** (R = MeO, Ar = 4-MeC_6_H_4_) showed the highest activity against *Candida albicans* with a diameter of growth of the inhibition zone of 16.3 mm and a MIC value of 64 μg/mL. Furthermore, compound **84k** displayed the highest activity against *Saccharomyces cerevisiae* with a diameter of growth of the inhibition zone of 16.6 mm and a MIC value of 64 μg/mL.

**Figure 8:**
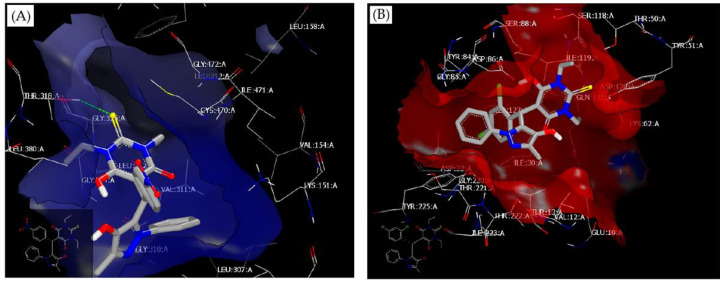
Ligand–receptor interaction profiles by molecular docking. (**A**) Interactions between 4WMZ and **61h**, and (**B**) interactions between 4WMZ and **61l**. Image adapted from Elshaier et al. [65].

In the same way, the series of pyrazole-thiobarbituric acid derivatives **61**, obtained via a four-component synthetic approach and discussed in Section 2.1. Antibacterial activity (Table 17) [65], was also evaluated for their antifungal activity against *Candida albicans* by the diffusion method and serial dilution method using fluconazole as a standard drug (Table 36). Overall, compounds **61** showed MIC values in the range of 4–64 μg/L against *Candida albicans*. In particular, the compounds **61h** and **61l** exerted significant activity against *Candida albicans* with a MIC value of 4 μg/L, in comparison to fluconazole (MIC = 0.5 μg/L). Later, docking molecular was performed for all compounds and fluconazole against Lanosterol 14 *α*-demethylase (CYP51A1) (PDB ID code: 4WMZ). The fluconazole (consensus score of 57) forms two hydrogen bonds with Thr318 via the nitrogen atom of the triazole moiety and Leu312 via the oxygen atom of the hydroxyl group. The compound **61h** (consensus score of 55) forms one hydrogen bond between Thr318 and the sulfur atom of the thiocarbonyl group (Figure 8A), while the compound **61l** (consensus score of 48) interacts with Thr318 via hydrophobic–hydrophobic interactions (Figure 8B).

**Figure 9:**
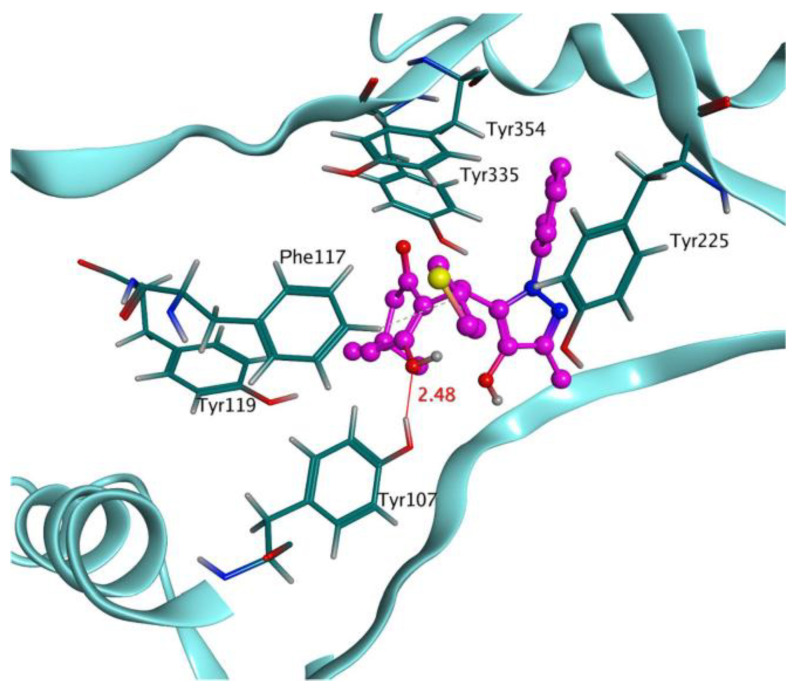
Docking molecular of the compound **21o** with *N*-myristoyl transferase (PDB ID code: 1IYL) from *Candida albicans*. Image adapted from Barakat et al. [31].

The pyrazole-dimedone derivatives **21a–o**, obtained via a three-component synthetic approach and discussed in Section 2.1. Antibacterial activity (Table 4) [31], were also evaluated for their antifungal activity against *Candida albicans* with MIC values in the range of 4–32 μg/L and a diameter of growth of the inhibition zone in the range of 13–21 mm, when compared to fluconazole as a standard drug (0.5 μg/L and 28 mm, respectively) (Table 37). Remarkably, the pyrazole-dimedone **21o** bearing thiophene was the most active against *Candida albicans* with a MIC value of 4 μg/L. The docking molecular of the compound **21o** against *N*-myristoyl transferase (NMT) (PDB ID code: 1IYL) from *Candida albicans* displayed a docking score of −8.7 kcal/mol and molecular interactions with the NMT enzyme. As shown in Figure 9, the hydroxyl group of the dimedone ring formed one hydrogen bond with Tyr107 at a distance of 2.48 Å. Apart from this, multiple hydrophobic and π–π electrostatic interactions were observed with crucial residues such as Tyr107, Phe117, Tyr119, Tyr225, and Tyr335.

Similarly, the polyhydroquinoline derivatives **51a–p**, obtained via a three-component process and discussed in Section 2.1. Antibacterial activity (Table 13) [54], wee also screened for their antifungal activity against *Candida albicans* and *Aspergillus fumigatus* using griseofulvin as a positive control (Table 38). The compounds **51j**, **51k**, and **51n** were found to be more active than griseofulvin (MIC = 500 μg/mL) against *Candida albicans*. Moreover, the compounds **51i** and **51l** showed the same antifungal activity as griseofulvin (MIC = 100 μg/mL) against *Aspergillus fumigatus*.

The pyrano[2,3-*c*]pyrazole derivatives **12**, obtained via a four-component process and discussed in Section 2.1. Antibacterial activity (Table 2) [28], were also screened for their antifungal activity against *Aspergillus flavus* and *Aspergillus niger* using ketoconazole as a standard drug (Table 39). It was found that at a 50 μg/well concentration, the compounds showed a diameter of growth of the inhibition zone in the range of 2–23 mm and 8–30 mm against *Aspergillus flavus* and *Aspergillus niger*, respectively, in comparison to ketoconazole (28 mm and 33 mm, respectively). In particular, compound **12f** showed better antifungal efficacy against *Aspergillus flavus* and *Aspergillus niger* with a diameter of growth of the inhibition zone of 23 mm and 30 mm, respectively, at a 50 μg/well concentration.

The pyrazolyl-dibenzo[*b*,*e*][1,4]diazepinones (**64–70**)**a** and (**71–77**)**b**, obtained via a multicomponent process and discussed in Section 2.1. Antibacterial (Table 18) [70], were also evaluated for their antifungal activity. The MIC values for such compounds against *Aspergillus fumigates* and *Candida albicans* were determined using griseofulvin as a standard drug. The compounds showed MIC values ranging from 250 to > 500 μg/mL and 250 to 1000 μg/mL against *Aspergillus fumigates* and *Candida albicans*, respectively, when compared to griseofulvin (MIC = 100 μg/mL and 500 μg/mL, respectively). Although compounds are very poor in their antifungal potency against *Aspergillus fumigates*, the resistance in many cases against *Candida albicans* is not disappointing. For instance, the compounds **65a** (R = H, R^1^ = 4-MeC_6_H_4_O), **75b** (R = H, R^1^ = 4-ClC_6_H_4_S), **66a** (R = H, R^1^ = 4-ClC_6_H_4_O), **66′a** (R = COC_6_H_5_, R^1^ = 4-ClC_6_H_4_O), and **74b** (R = H, R^1^ = C_6_H_5_S) displayed better activity against *Candida albicans* with MIC values of 250 μg/mL, in comparison to griseofulvin (MIC = 500 μg/mL).

**Scheme 18:**
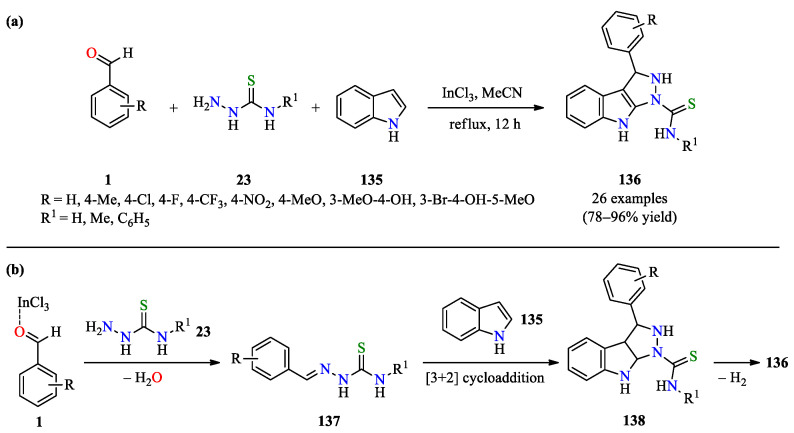
(**a**) Three-component synthesis of fused indolo-pyrazoles **136** for evaluation of their antifungal activity, (**b**) plausible mechanism for the synthesis of compounds **136**.

Recently, Makhanya et al. reported the InCl_3_-catalyzed synthesis of fused indolo-pyrazoles (FIPs) **136** up to 96% yield. In this approach, FIPs **136** were successfully obtained through a three-component reaction between aromatic aldehydes **1**, thiosemicarbazide derivatives **23**, and indole **135** in the presence of a catalytic amount of InCl_3_ in acetonitrile under reflux conditions (Scheme 18a) [122]. This approach shows a broad substrate scope and excellent functional group tolerance with diverse electron-rich and electron-deficient aromatic substrates. A plausible mechanism proposed by the authors is shown in Scheme 18b. The mechanism is triggered by the InCl_3_-catalyzed condensation reaction between aromatic aldehyde **1** and thiosemicarbazide derivative **23** to afford intermediate **137**. Thereafter, the [3+2] annulation reaction between indole **135** and Schiff base **137** and subsequent aromatization of the intermediate **138** enables the construction of a fused indolo-pyrazole scaffold. The antifungal activity was evaluated based on the diameter of the zone of inhibition (mm) against *Candida albicans*, *Candida utilis*, *Saccharomyces cerevisiae*, *Aspergillus flavus*, and *Aspergillus niger* using Amphotericin B as a standard drug. Overall, the compounds **136a–z** showed a diameter of growth of the inhibition zone ranging from 0 to 23 mm, in comparison to amphotericin-B (22 to 32 mm). Particularly, compound **136t** (R = 4-Cl, R^1^ = Me) showed moderate potency against *Candida albicans* and *Candida utilis* with an inhibition diameter of 15 and 14 mm, respectively, while the compound **136x** (R = 4-MeO, R^1^ = Me) showed good activity against *Saccharomyces cerevisiae* with an inhibition diameter of 20 mm, when compared to amphotericin-B (32, 30, and 29 mm, respectively).

Some spiropyrrolidine-oxindoles **29**, obtained from a three-component process and discussed in Section 2.1. Antibacterial (Table 6) [33], were also screened for their antifungal activity against *Candida albicans* and *Malassezia pachydermatis* using ketoconazole as a positive control (Table 40). Overall, the compounds showed a diameter of growth of the inhibition zone in the range of 9–12 mm and 8–10 mm for *Candida albicans* and *Malassezia pachydermatis*, respectively, when compared to ketoconazole (28 mm and 26 mm, respectively). Moreover, the compounds showed MIC values in the range of 125–500 μg/mL for *Candida albicans* and *Malassezia pachydermatis*, when compared to ketoconazole (MIC = 25 μg/mL). Remarkably, compound **29j** showed better activity against *Candida albicans* and *Malassezia pachydermatis* with a MIC value of 125 μg/mL. Unfortunately, the diameter of the growth of the inhibition zone for compound **29j** was not reported.

All the pyrazole derivatives **9** and **10**, synthesized from four-component processes, each one, and discussed in Section 2.1. Antibacterial (Table 1) [27], were also screened against *Candida krusei*, *Aspergillus fumigatus*, and *Aspergillus niger* using the Broth microdilution method in the presence of griseofulvin and nystatin as standard drugs (Table 41). Overall, the compounds showed a good antifungal activity with MIC values in the range of 3.75–12.5 μg/mL against three strains of fungi, in comparison to griseofulvin (MIC = 1.25–3.12 μg/mL) and nystatin (MIC = 1.00–1.25 μg/mL). Notably, compounds **9c** and **10e** displayed better antifungal activity against *Candida krusei* with a MIC value of 3.75 μg/mL. In addition, the compounds **9e** and **10c** showed a MIC value of 3.75 μg/mL against *Aspergillus fumigatus* and *Aspergillus niger*, respectively, which were almost equally active compared to griseofulvin (MIC = 1.25 μg/mL) and nystatin (MIC = 1.00 μg/mL) as standard drugs.

The synthesis of 4-[(3-aryl-1-phenyl-1*H*-pyrazol-4-yl)methylidene]-2,4-dihydro-3*H-*pyrazol-3-ones **34a–j** was reported by Sivaganesh et al. [35] and previously discussed in Section 2.1. Antibacterial (Table 7). The obtained compounds were also screened against *Aspergillus niger* and *Sclerotium rolfsii* fungal strains at a 500 μg/mL concentration by the disc diffusion method using ketoconazole as a standard drug (Table 42). These compounds showed acceptable activity against *Aspergillus niger* and *Sclerotium rolfsii* with a zone of inhibition in the range of 6.8–9.8 mm and 8.5–13.2 mm, respectively, when compared to ketoconazole (18.3 mm and 22.1 mm, respectively). Interestingly, compounds **34a** and **34j** showed the best antifungal potency against *Aspergillus niger* and *Sclerotium rolfsii* with a zone of inhibition of 9.8 mm and 13.2 mm, respectively. In addition, the compounds **34h**, **34c**, and **34a** displayed moderate activity against *Sclerotium rolfsii* with a zone of inhibition of 11.3, 12.0, and 12.8 mm, respectively.

In the same way, a series of 3-alkyl-1-(4-(aryl/heteroaryl)thiazol-2-yl)indeno[1,2-*c*]pyrazol-4(1*H*)-ones **37a–l** was reported by Mor et al. [36], and previously discussed in Section 2.1. Antibacterial (Table 8). These compounds were also screened for their antifungal activity against *Candida albicans* and *Aspergillus niger* showing MIC values in the range of 0.0067–0.0635 μmol/mL and 0.0270–0.1258 μmol/mL, respectively, when compared to fluconazole (MIC = 0.0408 μmol/mL) as a positive control (Table 43). Interestingly, the indenopyrazole **37d** displayed the highest potency against *Candida albicans* and *Aspergillus niger* with MIC values of 0.0067 and 0.0270 μmol/mL, respectively, in comparison to the standard drug fluconazole (MIC = 0.0408 μmol/mL).

**Scheme 19:**
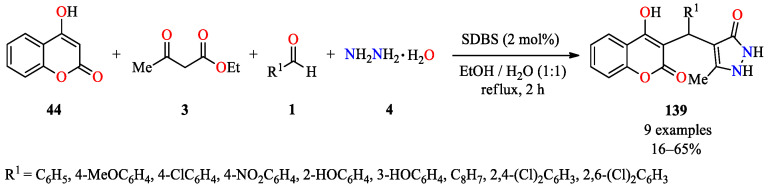
One-pot four-component synthesis and antifungal activity of benzylpyrazolyl-coumarin derivatives **139**.

Alternatively, a one-pot multicomponent protocol has been described for the synthesis of various benzylpyrazolyl-coumarins **139** in 16–65% yields through a reaction of 4-hydroxycoumarin **44**, ethyl acetoacetate **3**, diverse aldehydes **1**, and hydrazine hydrate **4** catalyzed by sodium dodecyl benzene sulfonate (SDBS, 2 mol%) in an ethanol–water mixture at reflux for 2 h (Scheme 19) [123]. This new approach is distinguished by its short reaction time, recovery of the catalyst, and reuse without loss of activity. The synthesized compounds **139a–i** were screened against *Candida albicans* by the disk diffusion technique using ketoconazole as a standard drug. The compounds **139a–i** showed moderate antifungal activity with a diameter of the inhibition zone in the range of 7–11 mm at a 5 mg/mL concentration, in comparison to ketoconazole (22 mm). Moreover, the compounds **139a–i** showed MIC values ranging from ≥125 to ≥500 μg/mL against *Candida albicans*, when compared to ketoconazole (6.25 μg/mL). Interestingly, the compound **139e** (R^1^ = 2-OHC_6_H_4_) showed the highest antifungal activity with a MIC value of ≥125 μg/mL. Docking molecular was performed for compound **139e** and ketoconazole against *N*-myristoyl transferase (NMT) (PDB ID code: 1IYL) from *Candida albicans*. The compound **139e** showed better binding energy (−14.16 kcal/mol) than the standard drug ketoconazole (−12.91 kcal/mol). According to the docking calculations results, the phenyl ring of chromen-2-one and the 2-hydroxyphenyl moiety of compound **139e** formed π–π interactions with Phe176 and Phe117. The hydroxyl group of chromen-2-one ring formed a hydrogen bond with Leu451. In addition, the oxygen atom and phenyl ring of the chromen-2-one moiety formed a hydrogen bond and π–anion interaction with Tyr107 and Leu451, respectively. Apart from this, multiple hydrophobic interactions were observed with crucial residues such as Val108 and Leu415.

### 2.4. Antioxidant Activity

Antioxidants are radical scavengers that help in delaying or preventing oxidation by trapping free radicals such as the superoxide radical (O_2_^•–^), hydroxyl radical (OH^•^), and lipid peroxide radicals [124,125]. In consequence, they are essential to relieve the oxidative stress and production of reactive oxygen species (ROS), and subsequently decrease diverse degenerative diseases of aging [125,126]. Due to their protective roles in food and pharmaceutical products, the discovery of heterocyclic compounds with antioxidant properties continues to be of great interest to the scientific community. For instance, a series of dihydropyrano[2,3-*c*]pyrazole derivatives **90** was reported by Ambethkar et al. [82], and previously discussed in Section 2.1. Antibacterial (Table 24). These compounds were also screened for their antioxidant activity against 2,2-diphenyl-1-picrylhydrazyl (DPPH) radical scavenger at concentrations ranging from 25 to 100 µg/mL using ascorbic acid as a reference (Table 44). Notably, the compounds **90i** and **90k** at a 100 µg/mL concentration displayed significant scavenging capacity with 60.65% and 57.82% inhibition, respectively, when compared to the ascorbic acid (98.85%). These results revealed that the presence of a hydroxyl group at the *para* position could extend the π-conjugation for stabilizing the formed free radical.

A series of pyrazole-containing pyrimidines **52**, 1,4-dihydropyridines **53**, and imidazoles **54**, obtained via a multicomponent approach and previously discussed in Section 2.1. Antibacterial activity (Table 14) [58], were also screened for their antioxidant activity. In summary, the pyrimidine derivatives **52c** (R = Cl, R^1^ = Me) and **52f** (R = F, R^1^ = Me) displayed better DPPH radical scavenging activity with 89.41% and 83.34%, respectively, as compared to glutathione (89.09%). By comparing the antioxidant results, a gradual decrease in the activity of the acetyl (–CO–Me) substituent was observed, followed by methoxy (–CO–OMe) and ethoxy (–CO–OEt) ester substituents. These results showed that modulation of the basic structure through ring substituents and/or additional functionalization decreases the antioxidant activity.

Additional to the antibacterial activity previously discussed in Table 2 (Section 2.1. Antibacterial activity) for the pyranopyrazole derivatives **12** obtained via a five-component synthesis [28], their DPPH and H_2_O_2_ radical scavenging activity were also investigated using ascorbic acid as a standard drug. In a DPPH assay, the compounds **12a** (R = 4-MeOC_6_H_4_) and **12j** (R = 2-MeOC_6_H_4_) showed higher IC_50_ values of 34.35 μg/mL and 35.93 μg/mL, respectively, when compared to the standard drug (39.51 μg/mL). In the H_2_O_2_ radical scavenging assay, compound **12a** (38.71 μg/mL) displayed similar activity to standard ascorbic acid (39.47 μg/mL), whereas compound **12j** (44.05 μg/mL) exhibited moderate activity. Importantly, the results of DPPH and H_2_O_2_ radical scavenging activity studies showed a linear correlation.

The pyrazolyl-dibenzo[*b*,*e*][1,4]diazepinones **(64–70)a** and **(71–77)b** were obtained via a multicomponent synthetic approach and previously discussed in Section 2.1. Antibacterial activity (Table 18) [70]. In addition, ferric reducing antioxidant power (FRAP) values were determined using Benzie and Strain’s modified FRAP method. Overall, the compounds **65a** (R = R^1^ = H, R^2^ = 4-MeC_6_H_4_O), **77b** (R = H, R^1^ = Me, R^2^ = PhCH_2_S), and **75b** (R = H, R^1^ = Me, R^2^ = 4-ClC_6_H_4_S) registered better FRAP values with 459, 464, and 468 (mm/100 g), respectively, indicating that they are good in resistance to the reduction of the ferric tripyridyl triazine (Fe(III)-TPTZ) complex into a blue color ferrous tripyridyl triazine (Fe(II)-TPTZ) complex.

**Scheme 20:**
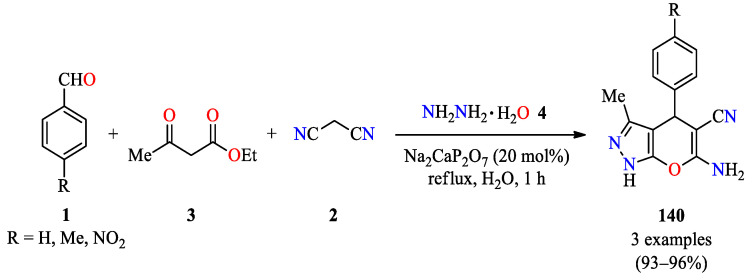
Na_2_CaP_2_O_7_-Catalyzed four-component synthesis of pyrano[2,3-*c*]pyrazole derivatives **140** and evaluation of their antioxidant activity.

Recently, the Na_2_CaP_2_O_7_-catalyzed synthesis of pyrano[2,3-*c*]pyrazoles derivatives **140** has been successfully implemented through a four-component reaction of aromatic aldehydes **1**, ethyl acetoacetate **3**, malononitrile **2**, and hydrazine hydrate **4** in refluxing water for 1 h (Scheme 20) [127]. In the DPPH assay, the compounds **140a** (R = H) and **140b** (R = Me) showed significant scavenging effects, while the compound **140c** (R = NO_2_) displayed a very low effect. In addition, the pyrano[2,3-*c*]pyrazole derivatives **140** had cytoprotective properties against the harmful effects of stressors, such as H_2_O_2_ and SNP (sodium nitroprusside) by quenching free radicals, while improving the activities of antioxidant enzymes.

**Scheme 21:**
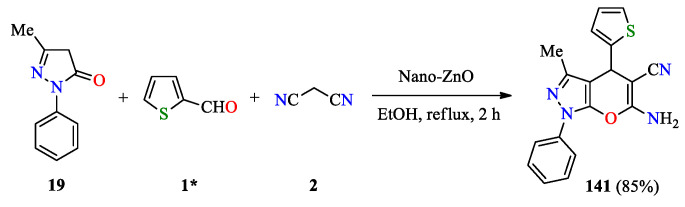
Three-component synthesis of the pyrano[2,3-*c*]pyrazole-5-carbonitrile **141** and evaluation of its antioxidant activity.

Very recently, the nano-ZnO-catalyzed reaction of 5-methyl-2-phenyl-2,4-dihydro-3*H*-pyrazol-3-one **19**, thiophene-2-carbaldehyde 1*, and malononitrile **2** in refluxing ethanol for 2 h has been reported for the synthesis of the pyrano 2,3-c]pyrazole-5-carbonitrile **141** in 85% yield (Scheme 21) [128]. Although the scope of the reaction was not further studied, compound **141** was used in the construction of an important library of highly functionalized pyrano[2,3-*c*]pyrazole derivatives without using a multicomponent approach. The synthesized compound **141** was screened for its antioxidant activity. As a result, it exhibited a total antioxidant activity of 22.26 U/mL, which is similar to standard ascorbic acid (29.40 U/mL).

**Scheme 22:**
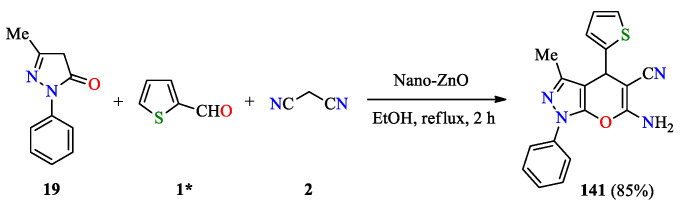
Three-component synthesis of tetrahydrobenzo[*b*]pyran derivatives **142** with antioxidant activity.

The eco-friendly three-component reaction of 3-aryl-1-phenyl-1*H*-pyrazole-4-carbaldehydes **31**, dimedone **20**, and malononitrile **2** catalyzed by *L*-proline (10 mol%) in refluxing aqueous ethanol for 30–60 min has been reported for the synthesis of tetrahydrobenzo[*b*]pyran derivatives **142** (Scheme 22) [129]. The solids were filtered and washed with a mixture of ethanol/water (1:1, *v/v*) to afford pyrazole derivatives **142** in 71–83% yields. All the synthesized compounds **142a–i** were screened for their antioxidant activity against the 2,2-diphenyl-1-picrylhydrazyl (DPPH) radical scavenger at the concentration of 1.0 mM using ascorbic acid as a positive control. Notably, the compounds **142a** (R^1^ = 4-NO_2_) and **142b** (R^1^ = 4-Br) at a 1.0 mM concentration showed better scavenging capacity with 61.87% and 60.62% inhibition, respectively, when compared to the ascorbic acid (72.00%).

### 2.5. α- Glucosidase and α-Amylase Inhibitory Activity

**Scheme 23:**
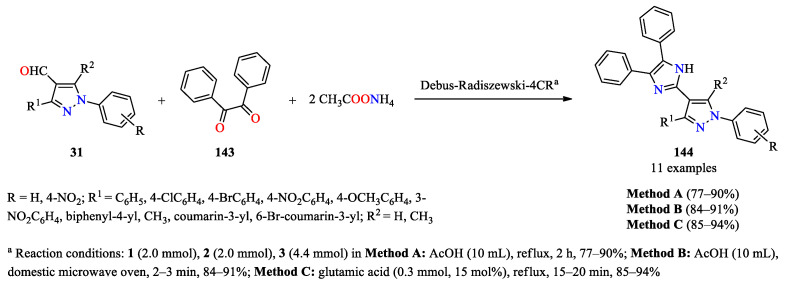
Pseudo four-component synthesis of imidazolylpyrazoles **144** as *α*-glucosidase inhibitors.

Type 2 diabetes is the most common form of diabetes, which is a challenging metabolic disease characterized by insulin resistance, leading to hyperglycemia or abnormal blood glucose levels and damage to various physiological processes in the body [130]. The significant increase in the number of people affected by this disease and its worldwide spread makes blood glucose control very complex in patients affected by type 2 diabetes [131,132]. Other problems are the side effects of antidiabetic drugs currently used for its treatment [133,134]. One aspect to be taken into account in the development of new antidiabetic drugs is the relationship between diabetes mellitus and the inhibition of hydrolase enzymes such as *α*-glucosidases and *α*-amylases, showing that the incorporation of azole-type heterocycles such as pyrazole, imidazole, and triazole, among others, is required in the design of new antihyperglycemic agents with higher activity than acarbose [135]. For instance, Chaudhry et al. described a multicomponent Debus–Radiszewski reaction to efficiently prepare imidazolylpyrazoles **144** in 77–90% yields from pyrazole-4-carbaldehydes **31** obtained by the Vilsmeier–Haack formylation reaction [24,136], benzil **143**, and ammonium acetate in refluxing acetic acid for 2 h (Scheme 23). The same reaction was conducted under microwave heating using a domestic oven to afford products **144** in 84–91% yields after short reaction times (2–3 min) [137]. Finally, the use of glutamic acid (15 mol%) as a catalyst at reflux for 15–20 min furnished products **144** in 85–94% yields.

**Figure 10:**
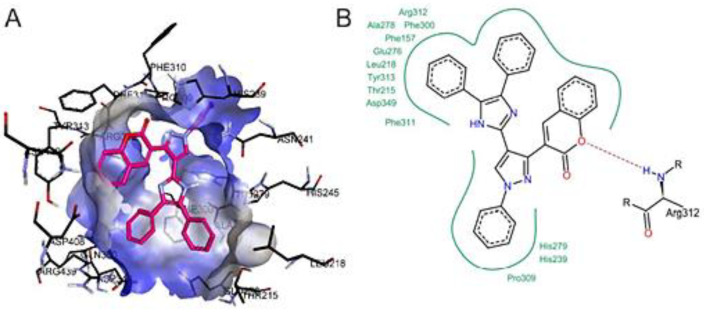
(**A**) Overall structure of the oligo-1,6-glucosidase (PDB ID: 3A4A) from *Saccharomyces cerevisiae* with compound **144j**, and (**B**) 2D interactions for compound **144j**. Image adapted from Chaudhry et al. [137].

In vitro *α*-glucosidase inhibition assays of imidazolylpyrazoles **144a–k** showed an inhibitory effect compared to control acarbose (IC_50_ = 38.25 µM), with the most potent inhibitors being those that contain substituents such as the coumarinyl ring, which exhibited percentages of inhibition at 98.56% (0.5 mM) with an IC_50_ value of 2.78 µM in compound **144j** (R^1^ = coumarin-3-yl, R = R^2^ = H) and 97.69% (0.5 mM) with an IC_50_ value of 2.95 µM in compound **144k** (R^1^ = 6-Br-coumarin-3-yl, R = R^2^ = H) [137]. According to molecular docking studies, the activity of the compound **144j** can be explained through a hydrogen bond interaction between the oxygen atom of the coumarin ring and Arg312 at the active site of the oligo-1,6-glucosidase (PDB ID: 3A4A) from *Saccharomyces cerevisiae* (Figure 10). The imidazolylpyrazoles containing electron-withdrawing groups such as R^1^ = 4-ClC_6_H_4_ (**144b**), 4-BrC_6_H_4_ (**144c**), and 4-NO_2_C_6_H_4_ (**144h**), where R and R^2^ = H, have markedly improved activity via the binding of the substrate with the target positions. Therefore, the comparative study has helped to find some key structural elements that could give rise to promising anti-diabetic compounds.

The same authors carried out structural modifications to generate imidazole–pyrazole hybrids as potent *α*-glucosidase inhibitors [138]. Thus, the multicomponent Debus–Radziszewski reaction of pyrazole-4-carbaldehydes **31**, benzyl **143**, substituted anilines **32**, and ammonium acetate under microwave irradiation afforded a series of imidazole–pyrazole hybrids **145** in 69–88% yields. This multicomponent approach shows high compatibility with pyrazole-4-carbaldehydes **31** containing electron-donating and electron-withdrawing groups. The imidazolylpyrazoles **145** were screened against *α*-glucosidase using acarbose as a standard drug (Table 45). In particular, the compounds containing electron-withdrawing substituents such as **145f** and **145m** showed a better inhibitory effect with IC_50_ values of 25.19 μM and 33.62 μM, respectively, when compared to acarbose (IC_50_ = 38.25 μM).

**Figure 11:**
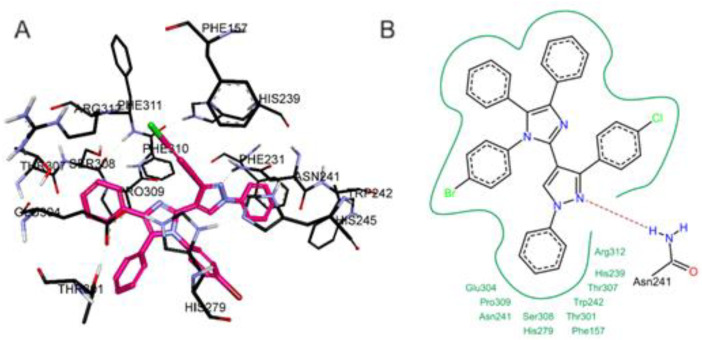
(**A**) The overall structure of the oligo-1,6-glucosidase (PDB ID: 3A4A) from *Saccharomyces cerevisiae* with compound **145f**, and (**B**) 2D interactions for compound **145f**. Image adapted from Chaudhry et al. [138].

Later, a homology model was constructed using oligo-1,6-glucosidase (PDB ID: 3A4A) from *Saccharomyces cerevisiae* as the protein template because it exhibits one of the best results and also shares 72% identity and 85% similarity of the *α*-glucosidase sequence. Figure 11 shows 3D and 2D interactions of the most likely coupled conformation of the compound **145f** with the homology model. It was found that imidazoylpyrazole **145f** presented a significant fit to the binding cavity, with the hydrogen bond interaction between the unsubstituted nitrogen atom of the pyrazole ring and Asn241 being important [138].

**Scheme 24:**
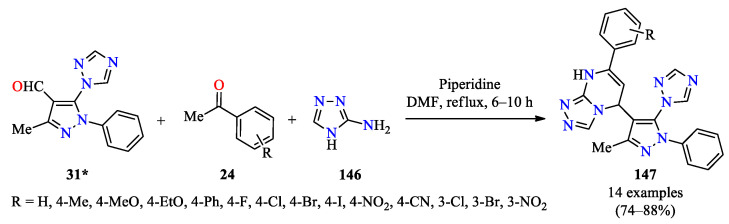
Three-component synthesis of pyrazole-triazolopyrimidine hybrids **147** as *α*-glucosidase inhibitors.

**Scheme 25:**
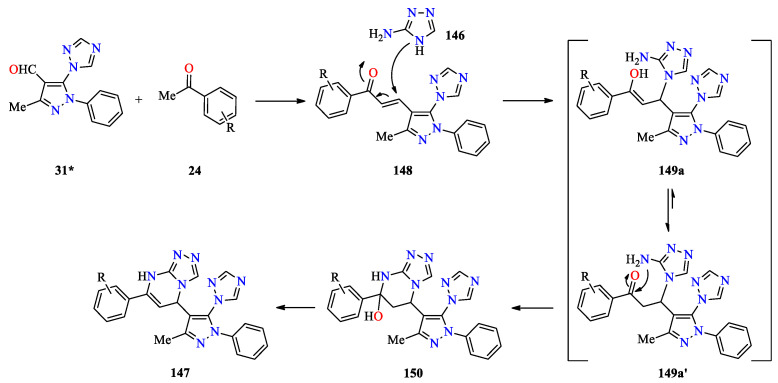
The plausible mechanism for the synthesis of pyrazole-triazolopyrimidine hybrids **147**.

Remarkably, Pogaku et al. described an interesting one-pot multicomponent approach to synthesize new pyrazole-triazolopyrimidine hybrids **147** as potent *α*-glucosidase inhibitors (Scheme 24) [139]. The synthesis of compounds involved an optimization process varying the base, solvent, and reaction time. Thus, the one-pot three-component reaction of pyrazole-4-carbaldehydes 31*, substituted acetophenones **24**, 4*H*-1,2,4-triazol-3-amine **146**, and a slight excess of piperidine in refluxing DMF for 6–10 h afforded pyrazole-triazolopyrimidine hybrids **147** in 74–88% yields. The plausible mechanism for the synthesis of pyrazole derivatives **147** is illustrated in Scheme 25. Initially, Claisen–Schmidt condensation of pyrazole-4-carbaldehydes 31* with substituted acetophenones **24** afforded chalcones **148**, which subsequently reacted with 4*H*-1,2,4-triazol-3-amine **146** to generate enol/keto intermediates **149a**/**149a’**. Finally, keto forms suffered an intramolecular cyclization/dehydration sequence to give pyrazole-triazolopyrimidine hybrids **147**.

The synthesized compounds **147** were tested against *α*-glucosidase using acarbose as a standard drug [139]. It was observed that compounds substituted with electron-withdrawing groups on the phenyl ring showed higher inhibitory activity against *α*-glucosidase than compounds with electron-donating groups. Among them, the compounds **147h** (R = 4-Cl), **147f** (R = 4-F), and **147l** (R = 4-NO_2_) exhibited the highest inhibitory activity with IC_50_ values of 12.45, 14.47, and 17.27 µM, when compared to acarbose (IC_50_ = 12.68 µM). Moreover, in silico docking studies of the ligand **147h** with the active site of the *α*-glucosidase (PDB ID: 3WY1) were performed using the GOLD 5.6 tool. From the two chains of *α*-glucosidase, the A chain with the polyacrylic acid as the crystalline co-ligand was selected. The compound **147h** forms hydrophobic, van der Waals, and hydrogen bond interactions with various amino acids of the active site of *α*-glucosidase. The formation of a hydrogen bond between the nitrogen atom of the triazole and the oxygen atom of the Asp202 was observed, as well as a hydrogen bond between Asp62 and the nitrogen atom of the pyrazole moiety. In addition, the residue Asp333 forms two hydrogen bonds with nitrogen atoms of triazole and pyrimidine rings.

In 2019, Duhan et al. described the three-component synthesis of thiazole-clubbed pyrazole hybrids **151** from 1-aryl-3-phenyl-1*H*-pyrazole-4-carbaldehydes **31**, thiosemicarbazide **23**, and substituted *α*-bromoacetophenones **36** in refluxing EtOH for 5 min (Table 46) [140]. The formed solids were filtered, dried, and recrystallized from ethanol to afford thiazole–pyrazole hybrids **151** in 71–89% yields after short reaction times. All synthesized pyrazole derivatives **151** were screened for their *α*-amylase activity at three different concentrations (12.5, 25, and 50 μg/mL) using acarbose as a standard drug. At a 50 μg/mL concentration, the synthesized compounds **151a–r** exhibited a percentage of inhibition in the range from 70.04% to 89.15%, when compared to acarbose (77.96%). In particular, the compounds **151g** and **151h** displayed a significant percentage of inhibition with values of 89.15% and 88.42%, respectively, in comparison to acarbose (77.96%).

**Figure 12:**
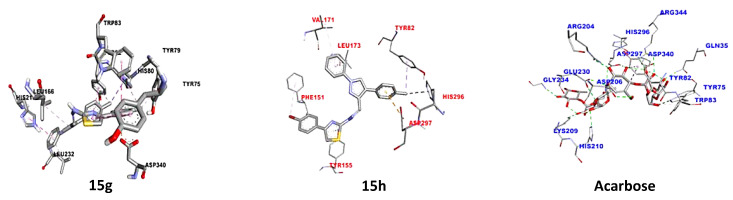
3D Interactions of compounds **151g**, **151h**, and acarbose with binding sites of *Aspergillus oryzae α*-amylase (PDB ID: 7TAA). Image adapted from Duhan et al. [140].

Additionally, molecular docking studies of the most potent compounds **151g** and **151h** were performed with the active site residues of *Aspergillus oryzae α*-amylase (PDB ID: 7TAA) to establish the binding conformation and interactions associated with the activity [140]. As shown in Figure 12, compound **151g** showed four hydrophobic, one hydrogen bond, and one electrostatic interaction, while compound **151h** displayed one electrostatic, one hydrogen bond, and eleven hydrophobic interactions. As a result, the binding interactions found for compounds **151g** and **151h** with *α*-amylase were similar to those responsible for *α*-amylase inhibition by acarbose.

**Figure 13:**
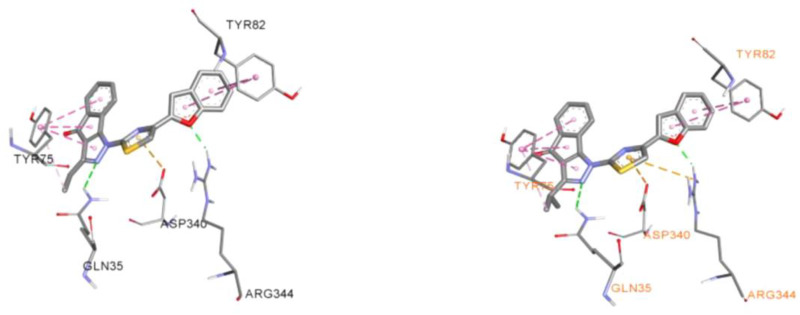
3D Interactions of indenopyrazoles **37j** (left) and **37k** (right) with binding sites of *Aspergillus oryzae α*-amylase (PDB ID: 7TAA). Image adapted from Mor et al. [36].

On the other hand, a series of 3-alkyl-1-(4-(aryl/heteroaryl)thiazol-2-yl)indeno[1,2-*c*]pyrazol-4(1*H*)-ones **37a–l** was obtained via the one-pot three-component approach discussed in Section 2.1. Antibacterial activity (Table 8) [36]. The pyrazole derivatives **37a–l** were also screened for their *α*-amylase activity by using the starch-iodine method in the presence of acarbose as a standard drug (Table 47). Overall, compounds **37a–l** showed IC_50_ values in the range of 0.46−20.51 μM, when compared to acarbose (IC_50_ = 0.11 μM). Interestingly, compounds **37j** and **37k** resulted in being the best inhibitors of *α*-amylase with IC_50_ values of 0.79 μM and 0.46 μM, respectively. In addition, the compounds **37i** and **37l** displayed good inhibitory activity with IC_50_ values of 0.94 μM and 0.89 μM, respectively, whereas the compounds **37a**, **37e**, **37f**, and **37h** were found to be moderately active with IC_50_ values in the range of 3.21−6.88 μM. To determine the binding conformation, molecular docking for indenopyrazoles **37j** and **37k** was performed in the active site of *Aspergillus oryzae α*-amylase (PDB ID: 7TAA). The binding affinity of compounds **37j**, **37k**, and acarbose are −8.7, −9.0, and −10.1 kcal/mol, respectively. As shown in Figure 13, the oxygen atom of the benzofuran ring forms a hydrogen bond with Arg344, while the nitrogen atom of the pyrazole ring interacts with Gln35 via a hydrogen bond. The indenopyrazole and benzofuran ring form π–π stacked interactions with Tyr75 and the aromatic ring of the Tyr82, respectively. Finally, the thiazole ring forms π–anion interactions with Asp340 in both compounds, and π–cation interactions with Arg344 only in compound **37k**.

### 2.6. Anti-Inflammatory Activity

Inflammation is part of the complex biological response of vascular tissues to harmful stimuli, such as pathogens, damaged cells, or irritants [141,142]. Essentially, inflammation involves the production of pro-inflammatory mediators, an influx of innate immune cells, and tissue destruction [141,142]. In this sense, steroidal and non-steroidal anti-inflammatory drugs have been extensively employed to inhibit the production of pro-inflammatory prostaglandins (PGs) [143]. In recent decades, NSAIDs have become a widely used therapeutic group due to fewer adverse effects or other side effects such as renal impairment and gastric ulcers [143]. In consequence, the development of efficient and safer anti-inflammatory drugs is still highly desired in chemical biology and drug design. In this way, the series of 4-coumarinylpyrano[2,3-*c*]pyrazole derivatives **93**, obtained via a one-pot four-component approach and discussed in Section 2.1. Antibacterial activity (Table 26) [87], was also subjected to an anti-inflammatory effect against the denaturation of hen’s egg albumin method at the concentration of 31.25 μM with aceclofenac as a standard drug (Table 48). Compounds **93** showed very high activity against the denaturation of protein with inhibition percentages ranging from 7.60% to 51.43%. Remarkably, the compounds **93g**, **93h**, and **93j** showed excellent activity with inhibition percentages of 51.43%, 39.06%, and 37.65%, respectively, which are more active compared to the standard aceclofenac drug (5.50%). Moreover, the anti-inflammatory activity was also screened using the Human Red Blood Cell (HRBC) membrane stabilization technique at the concentration of 100 μM with the standard acetyl salicylic acid drug. Notably, the compounds **93g**, **93h**, and **93j** exhibited good activity with inhibition percentages of 54.06%, 39.86%, and 38.56%, respectively, in comparison with the standard acetyl salicylic acid drug (36.16%).

The thiazolo[2,3-*b*]dihydropyrimidinones **80a**–**p**, obtained via a three-component synthetic approach and discussed in Section 2.1. Antibacterial activity (Table 19) [72], was also screened for its anti-inflammatory activity using the carrageenan-induced paw edema method of inflammation in rats at successive intervals of 1, 2, and 4 h compared with the standard indomethacin drug. The tested compounds **80a**–**p** exhibited moderate anti-inflammatory activity within 2 h, while the activity increased and reached peak level at 4 h and declined after 4 h. Remarkably, the compounds **80a** (R = 3-F-4-Me, R^1^ = R^2^ = Cl), **80e** (R = 3-F-4-Me, R^1^ = R^2^ = F), and **80i** (R = 3-F-4-Me, R^1^ = Cl, R^2^ = H) showed potent anti-inflammatory activity at 4 h with 85.33%, 81.32%, and 80.75% inhibition of the edema, respectively, which is comparable with the standard indomethacin drug (86.76%). Later, the synthesized compounds **80a**–**p** were docked into the active sites of the COX-2 enzyme. The molecular docking revealed that compounds **80a**, **80e**, and **80i** were more selective towards the COX-2 active site with calculated binding energy of −540.47, −315.73, and −129.88 kcal/mol, respectively, involving a hydrogen bonding between the nitrogen atom of the pyrimidine ring and hydrogen atom of the amino group into ARG 120. These results are in good agreement with experimental results.

Similarly, the pyrazole derivatives **16a–c**, obtained via a three-component process and discussed in Section 2.1. Antibacterial activity (Scheme 2) [29], were also screened for their anti-inflammatory activity in rats at successive intervals of 1, 2, 4, and 6 h. The standard indomethacin drug (10 mg/kg) and tested heterocycles (50 mg/kg) were administered to the rats 30 min before the injection of 0.1 mL of 1% carrageenan suspension in normal saline. The reduction in edema volume reached a maximum level at 6 h ranging from 9.30% to 13.31%, which is compared to the standard indomethacin drug (16.27%). In particular, pyrazole derivatives **16a** (R = H) and **16c** (R = 2,4-(NO_2_)_2_C_6_H_3_) displayed the highest reduction in edema volume with 13.31% and 12.26%, respectively.

The pyrano[2,3-*c*]pyrazole-5-carbonitrile **141**, obtained via a multicomponent process and discussed in Section 2.4. Antioxidant (Scheme 21) [128], was also evaluated for its anti-inflammatory activity using celecoxib and quercetin as standard drugs. Notably, the compound **141** exhibited significant activity against COX-1 and COX-2 enzymes with values of 5.47 and 0.25 μM, respectively, in comparison to the standard celecoxib (14.60 and 0.04 μM, respectively). It also inhibited the LOX enzyme with a value of 4.43 μM, which is comparable to the standard quercetin (3.35 μM).

Tetrahydrobenzo[*b*]pyran derivatives **142**, obtained from a three-component process and discussed in Section 2.4. Antioxidant (Scheme 22) [129], were also screened for their anti-inflammatory activity using the protein denaturation method at the concentration of 1.0 mM using diclofenac as a standard drug. Importantly, the compounds **142i** (R^1^ = 3-NO_2_), **142a** (R^1^ = 4-NO_2_), and **142d** (R^1^ = 4-MeO) exhibited good activity with inhibition percentages of 69.72%, 65.13%, and 63.30%, respectively, in comparison with the standard diclofenac drug (90.21%).

### 2.7. Antimycobacterial Activity

Tuberculosis (TB) is a disease caused by *Mycobacterium tuberculosis* that claims approximately 1.5 million deaths every year [144]. When *M. tuberculosis* is not treated adequately, it takes the form of MDR-TB (multidrug-resistant TB) and XDR-TB (extensive-drug resistant TB) [144]. Thus, the re-emergence of tuberculosis has stimulated the search for new drugs against drug-resistant organisms, preferably acting on new targets [145,146]. Screening of new compounds has been carried out in several mycobacteria models. The main model used remains the infective agent, *M. tuberculosis* with H37Rv and H37Ra as virulent and avirulent reference strains, respectively [145,146]. In this way, Patel’s group reported the synthesis of thirteen examples of pyrazole-linked triazolo[1,5-*a*]pyrimidines **153** through a three-component reaction of pyrazole-4-carbaldehyde derivatives **31**, 1*H*-1,2,4-triazol-3-amine **146**, and *β*-ketoamides **152** containing a pyridine nucleus in refluxing DMF for specific time intervals as visualized by TLC (Table 49) [147]. After cooling, acetone (10 mL) was added, and the reaction mixture was stirred overnight at room temperature. Then, the solid was filtered, recrystallized from ethanol, and dried in the air. Later, the compounds **153** were evaluated for their anti-tuberculosis activity against *Mycobacterium tuberculosis* strain H37Rv using the Lowenstein–Jensen medium and broth dilution technique. The most significant results are summarized in Table 49. Particularly, the compounds **153a–e** inhibited *Mycobacterium tuberculosis* ranging from 95% to 99% at a 6.25 μg/mL concentration. Further, the secondary screening results showed that the compounds **153a** and **153b** inhibited the *mycobacterium* strain at MIC 3.13 and 1.56 μg/mL, respectively, while the compounds **153c**, **153d**, and **153e** inhibited *Mycobacterium tuberculosis* at a MIC lower than 1.0 μg/mL. In summary, the triazolo[1,5-*a*]pyrimidine derivatives **153** exhibited moderate anti-TB activity as compared to Isoniazid (MIC = 0.3 μg/mL) but good anti-TB activity as compared to Ethambutol and Rifampicin (MIC = 0.5 and 3.12 μg/mL, respectively).

**Figure 14:**
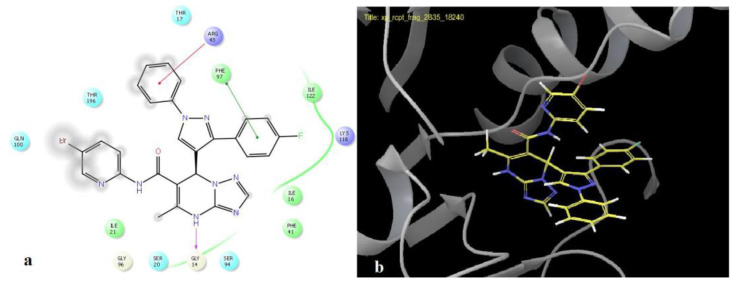
(**a**) 2D model enumerating the interactions between ligand **153c** and InhA enzyme, (**b**) 3D model of compound **153c** in the binding pocket of the InhA enzyme. Image adapted from Bhatt et al. [147].

It should be noted that compounds **153a–e** were found to be non-toxic against Vero cells (IC_50_ ≥ 20 μg/mL), while compounds **153c–e** displayed good mycobacterial enoyl-reductase (InhA) inhibitory potency with IC_50_ values of 0.11, 0.16, and 0.11 μg/mL, respectively (Table 49). In addition, molecular docking studies were performed for the most active compounds **153a–e** against the active site of the InhA enzyme (PDB: 2B35). As shown in Figure 14, the significant binding affinities with docking energies ranging from −44.89 to −56.60 kcal/mol and the RMS deviation values were observed to fall in the range of 2–3 Å, which can be considered to be an acceptable value of deviation [147]. As shown in Figure 14, the compound **153c** with binding energy of −49.48 kcal/mol and the considerably high XP glide score of −9.07 binds with the InhA active site forming a hydrogen bond between NH of the dihydropyrimidine ring and oxygen of the carboxylate group of Gly14 at a distance of 2.17 Å. Furthermore, π–π stacking was observed between the two phenyl rings linked to the pyrazole ring with the phenyl ring of Phe97 and amine group of Arg43, respectively.

Similarly, the polyhydroquinoline derivatives **51a–p**, obtained via a three-component process and discussed in Section 2.1. Antibacterial activity (Table 13) [54], were also screened against the *Mycobacterium tuberculosis* H37Rv strain at a 250 μg/mL concentration using isoniazid and rifampicin as standard drugs (Table 50). Remarkably, the polyhydroquinoline derivatives **51e**, **51i**, and **51l** exhibited significant antituberculosis activity with percentages of inhibition of 94%, 95%, and 91%, respectively, which are comparable to isoniazid and rifampicin (99% and 98%, respectively).

**Scheme 26:**
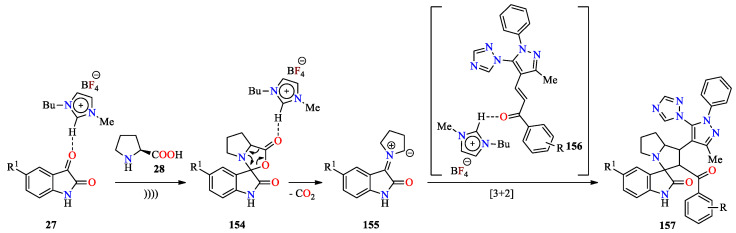
Plausible mechanism for the ultrasound-assisted multicomponent synthesis of spirooxindolopyrrolizidine derivatives **157**.

Ultrasonic irradiation has been frequently used in the synthesis of diverse *N*-heterocyclic systems of biological interest [105]. For instance, a series of 1,2,4-triazol-1-yl-pyrazole-based spirooxindolopyrrolizidines **157** have been synthesized in 76–92% yields by an ultrasound-assisted three-component reaction of pyrazole-based chalcones **156**, substituted isatins **27**, and *L*-proline **28** using an ionic liquid ([Bmim]BF_4_) at 60 °C for 6–16 min (Table 51) [148]. The [Bmim]BF_4_ is reused in up to five cycles without a significant change in yields and catalytic activity. This multicomponent approach is distinguished by its operational simplicity, high yielding, short reaction time, as well as easy separation and recyclability of the [Bmim]BF_4_. The plausible mechanism for the synthesis of compounds **157** is depicted in Scheme 26. It should be mentioned that the ionic liquid can act as both a solvent and catalyst through the interaction of its electron-deficient hydrogen atom with the oxygen atom of carbonyl groups. This interaction facilitates the polarization of the carbonyl group of isatin **27** to react with *L*-proline **28** for the formation of intermediate **154**, and subsequent decarboxylation leads to the highly reactive azomethine ylide **155**. Ultimately, the 1,3-dipolar cycloaddition reaction between the adjacent double bond of dipolarophile **156** and azomethine ylide **155** affords the spirooxindolopyrrolizidine system **157**.

The compounds **157a–p** were screened against the *Mycobacterium tuberculosis* H37Rv strain using ethambutol as a standard drug (Table 51) [148]. Most compounds **157** exhibited significant anti-TB activity with MIC values ranging from 0.78 to 12.5 μg/mL, except for compounds **157a**, **157b**, **157f**, **157i**, and **157p**, which presented MIC values equal to or greater than 20 μg/mL. Remarkably, the compound **157h** displayed higher anti-TB activity (MIC = 0.78 μg/mL) than the standard drug ethambutol (MIC = 1.56 μg/mL). Additionally, the cytotoxicity of the most potent anti-TB compounds was screened against the RAW 264.7 cell line at a 25 μg/mL concentration by adopting the MTT assay. In summary, the promising antituberculosis active compounds **157e**, **157h**, **157k**, **157l**, **157n**, **157q**, and **157r** exhibited a lower percentage of inhibition ranging from 17.91% to 27.15%.

The pyrazolyl-dibenzo[*b*,*e*][1,4]diazepinones **(64–70)a** and **(71–77)b** were obtained via a multicomponent synthetic approach and previously discussed in Section 2.1. Antibacterial activity (Table 18) [70]. These compounds exhibited anti-TB activity ranging from 6% to 90% at a 250 μg/mL concentration using isoniazid as a standard drug. In particular, the synthesized compounds **66a** (R = H, R^1^ = 4-ClC_6_H_4_O), **66′a** (R = COPh, R^1^ = 4-ClC_6_H_4_O), **75b** (R = H, R^2^ = 4-ClC_6_H_4_S), and **75′b** (R = COPh, R^2^ = 4-ClC_6_H_4_S) displayed better anti-TB activity with percentages of inhibition of 86%, 85%, 90%, and 88%, respectively, which are comparable to the standard drug isoniazid (99%).

### 2.8. Antimalarial Activity

Malaria is a disease caused by the parasite *Plasmodium*, which is transmitted by the bite of an infected mosquito belonging to the *Anopheles* genus [149]. Among the five *Plasmodium* species, *Plasmodium falciparum* is considered responsible for approximately 90% of malaria deaths worldwide [150,151]. For that reason, developing novel antimalarials with remarkable activity against all five human-infecting *Plasmodium* species is still highly desirable [150,151]. In this way, the polyhydroquinoline derivatives **51a–p**, obtained via a three-component process and discussed in Section 2.1. Antibacterial activity (Table 13) [54], were also screened for their in vitro antimalarial activity using chloroquine and quinine as standard drugs. Overall, the compounds **51a** (R = 4-F, R^1^ = CN, R^2^ = 4-Cl), **51c** (R = 4-F, R^1^ = CONH_2_, R^2^ = 4-Cl), and **51d** (R = 4-F, R^1^ = CN, R^2^ = 4-Me) exhibited IC_50_ values of 0.065, 0.085, and 0.076 μM, respectively, which is remarkable against *Plasmodium falciparum* as compared to chloroquine and quinine (IC_50_ = 0.020 and 0.268 μM, respectively). Later, molecular docking was performed between ligands (**51a**, **51c**, **51d**, chloroquine, and quinine) and the receptor wild-type *Plasmodium falciparum* dihydrofolate reductase-thymidylate synthase (PDB ID: 4DPD) [54]. The molecules interacted with the active pockets of protein by forming an H-bond. The docking scores of molecules **51a**, **51c**, and **51d** were found to be −27.88, −28.78, and −27.82, respectively, which were not as good compared to the standard drugs chloroquine and quinine with values of −49.93 and −45.82, respectively. The cyano group of compounds **51a** and **51d** and the carbonyl group of the amide **51c** interacted with the active pockets of the enzyme, forming hydrogen bonds with TYR 365. In addition, the C=O of the cyclohexane ring formed a hydrogen bond with LYS 297.

**Scheme 27:**
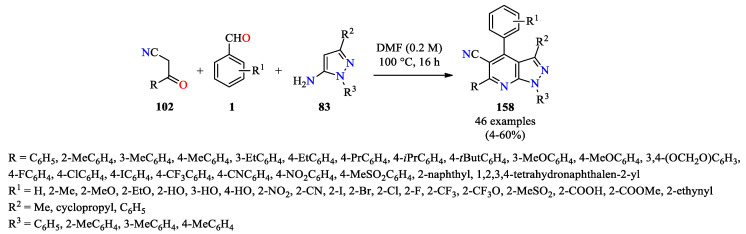
Three-component synthesis of densely substituted pyrazolo [3,4-*b*]pyridines **158** as antimalarial agents.

The pyrazolo[3,4-*b*]pyridine core has been proven to be antimalarial for overcoming the burden of resistance in *Plasmodium falciparum* [151]. Consequently, the simple and efficient synthesis of functionalized pyrazolo[3,4-*b*]pyridines continues to be an important factor in modern drug discovery [152]. In this way, Eagon et al. developed the three-component reaction of aryl-3-oxopropanenitrile derivatives **102**, aromatic aldehydes **1**, and *N*-aryl-5-aminopyrazoles **83** in DMF at 100 °C for 16 h to afford densely substituted pyrazolo[3,4-*b*]pyridines **158** (Scheme 27) [153]. In most cases, compounds **158** were obtained in low to moderate yields because the crude product was purified via trituration with methanol or absolute ethanol, and subsequent filtration and drying. Later, all synthesized compounds **158** were tested against the chloroquine-sensitive *Plasmodium falciparum* 3D7 strain grown in the presence of O-positive erythrocytes with EC_50_ values ranging from 0.0692 to 2.04 µM. Overall, most compounds displayed sub-micromolar potency against the intraerythrocytic stage of the parasite, with the most potent compound **158w** (R = 4-*t*ButC_6_H_4_, R^1^ = 2-OH, R^2^ = Me, R^3^ = C_6_H_5_) presenting an EC_50_ value of 0.0692 μM. Additional blood stage assays of the compound **158w** showed a moderate killing profile with no activity against the gametocyte stage of the parasite.

### 2.9. Miscellaneous Activities

#### 2.9.1. AChE and BChE Inhibitory Activity

Alzheimer′s disease (AD) is a progressive neurological disorder and the most common cause of dementia in the elderly. It is widely known that one of the possible treatments is the inhibition of acetylcholinesterase (AChE) and butyrylcholinesterase (BChE) to maintain the levels of the neurotransmitter ACh [154,155]. Currently, three AChE inhibitors have been approved by the US Food and Drug Administration for AD therapy: Donepezil, rivastigmine, and galantamine; however, these drugs neither cure nor stop the progression of the disease [154,155]. In the search for new ChE inhibitors, Derabli et al. provided the synthesis, docking studies, and in vitro anticholinesterase activity of new tacrine-pyranopyrazole analogs via a one-pot four-component reaction [156]. By mixing (hetero)aromatic aldehydes **1**, malononitrile **2**, and 3-methyl-1*H*-pyrazol-5-one **19** in refluxing 1,2-dichloroethane (DCE) generates pyrano[2,3-*c*]pyrazole intermediates **160**. Then, AlCl_3_-mediated Friedländer condensation with cyclohexanone **159** in the same flask gave rise to a library of tacrine-pyranopyrazoles **161** with good to excellent yields (Table 52). The use of aromatic or heterocyclic aldehydes did not have a significant impact on the yields.

The corresponding tacrine-pyranopyrazoles were evaluated on their in vitro anticholinesterase activity [156]. The data obtained for the inhibition of AChE/BChE by target compounds **161a–l** demonstrated that the majority of compounds had higher selectivity towards AChE than BChE with IC_50_ values ranging from 0.044 to 5.80 μM, wherein compounds **161e** and **161j** were found to be most active inhibitors against AChE with IC_50_ values of 0.058 and 0.044 μM, respectively, in comparison to Galantamine as the reference drug (IC_50_ = 21.82 μM). Afterward, molecular modeling simulation of compound **161j** with the AChE receptor showed two hydrogen bonds with Ser286 and one hydrogen bond with Tyr70. The *S* configuration was rotated in the opposite way to direct the biphenyl ring in the same direction as the *R* isomer. Since it was inversely oriented, it formed three hydrogen bonds with different amino acid residues such as Arg289 (two hydrogen bonds) and Tyr334 (one hydrogen bond). The amino group and pyrazole ring seemed to act as the pharmacophores for these interactions.

#### 2.9.2. Antihyperuricemic Activity

Xanthine oxidase (XO) is a complex molybdoflavoprotein that catalyzes the hydroxylation of xanthine and hypoxanthine by using molecular oxygen as an electron acceptor [157]. However, XO produces reactive oxygen species leading to oxidative damage to the tissue and a variety of clinical disorders [157]. In this sense, purine-based xanthine oxidase inhibitors such as allopurinol, pterin, and 6-formylpterin have been successfully utilized to prevent XO-mediated tissue damage. However, these inhibitors have been reported to be associated with Steven–Johnson syndrome and worsening of renal function in some patients [157,158]. In 2015, Kaur et al. developed the solvent-free four-component reaction of (hetero)aromatic aldehydes **1**, malononitrile **2**, ethyl 3-oxobutanoate **3**, and hydrazine hydrate **4** in the presence of DMAP as a catalyst under microwave heating at 150 °C for 20 min, affording pyrano[2,3-*c*]pyrazole derivatives **162** in high yields (Table 53) [158]. Later, an in vitro xanthine oxidase assay was conducted by the authors. Among a series of 19 compounds, 6 compounds were found to display a % age inhibition of >80%. It should be noted that molecules exhibiting % age inhibition of more than 80% at 50 μM were further tested for the xanthine oxidase inhibitory activity using allopurinol as a reference inhibitor (IC_50_ = 8.29 μM). Remarkably, non-purine xanthine oxidase inhibitors **162l** and **162m** displayed the most potent inhibition against the enzyme with IC_50_ values of 3.2 and 2.2 μM, respectively.

#### 2.9.3. Antileishmanial Activity

Leishmaniasis is a chronic infection caused by a protozoan parasite that belongs to the genus *Leishmania*. Among all forms of leishmaniasis, visceral leishmaniasis (VL) is the most serious form of the disease [159]. In recent years, several therapeutic options for VL have been employed such as the oral drug miltefosine, the aminoglycoside antibiotic paromomycin, and pentamidine [159,160]. Nevertheless, major concerns such as teratogenicity, nephrotoxicity, hepatotoxicity, ototoxicity, and unaffordable cost are associated with current antileishmanial chemotherapeutic agents [159,160]. Note that combining two or more potentially bioactive moieties to construct heterocyclic scaffolds is a known process in drug discovery [160]. In 2017, Anand et al. reported the three-component reaction of indole-based 5-aminopyrazoles **163**, aryl aldehydes **1**, and cyclic 1,3-diketones **44** in acetic acid at 100–110 °C for 2 h to afford pyrazolodihydropyridine derivatives **164** in 55–80% yields (Table 54) [161]. Most of the compounds were purified by crystallization in EtOH without the need for column chromatography. Later, in vitro antileishmanial activity of pyrazolodihydropyridines was evaluated at 25 μM and 50 μM concentrations against extracellular promastigotes and intracellular amastigotes of luciferase-expressing *Leishmania donovani.* As shown in Table 54, the compounds **164d** and **164j** displayed excellent activity with parasite killing >95% at 50 μM. Remarkably, IC_50_ values of **164d** and **164j** were found to be 7.36 μM and 4.05 μM against amastigotes, respectively, which is better than the antileishmanial drug miltefosine (IC_50_ = 9.46 μM).

#### 2.9.4. Antiurease Activity

**Scheme 28:**
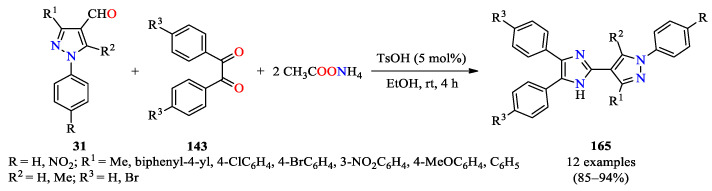
Pseudo-four-component synthesis of imidazolylpyrazoles **165** as antiurease agents.

From a medicinal perspective, urease is the major virulence factor of some lethal bacterial pathogens such as *Mycobacterium tuberculosis*, *Proteus mirabilis*, and *Helicobacter pylori*, among others [162]. Generally, it causes infections of the gastrointestinal tract, gastric ulcers, kidney stones, hepatic coma, and the risk of developing gastric cancer [163]. As a contribution to this topic, Chaudhry et al. reported a pseudo-four-component reaction of pyrazole-4-carbaldehyde **31**, symmetrical 1,2-diarylethane-1,2-diones **143**, and ammonium acetate catalyzed by *p*-toluenesulfonic acid in ethanol at ambient temperature, affording a series of imidazolylpyrazole derivatives **165** up to 94% yield in short reaction times (4 h) (Scheme 28) [164]. This TsOH-catalyzed approach resulted in being a simple and convergent method to prepare products via the formation of four C-N bonds in one-step.

**Figure 15:**
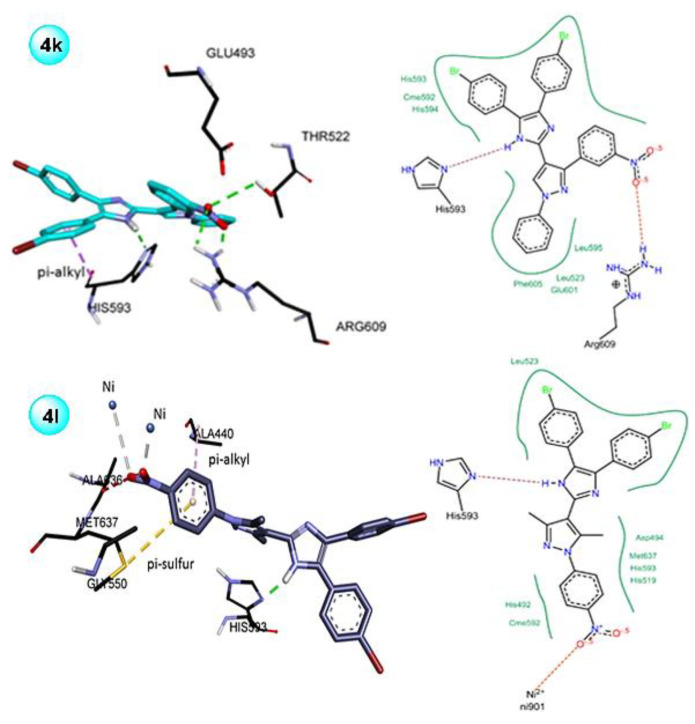
3D and 2D Interactions of compounds **165k** and **165l** with the amino acids of 4GY7. Image adapted from Chaudhry et al. [164].

The twelve synthesized compounds were tested for their antiurease activity with IC_50_ values ranging from 0.7 to 154.6 µM. Seven compounds were more potent compared to the standard thiourea (IC_50_ = 21.26 µM) as a positive control. Remarkably, the compounds **165k** (R = R^1^ = H, R^2^ = 3-NO_2_C_6_H_4_, and R^3^ = Br) and **165l** (R = NO_2_, R^1^ = R^2^ = Me, and R^3^ = Br) have excellent activity with IC_50_ values of 0.7 and 1.0 μM, respectively. Afterward, molecular docking studies were performed to understand the interaction mode of best inhibitors **165k** and **165l** with the active site of urease. The crystal structure of Jack bean’s (*Canavalia ensiformis*) urease [PDB ID: 4GY7, 1.49 Å] was selected for the study. As shown in Figure 15, the oxygen of the nitro group of inhibitor **165k** bonded with NH of ARG609 amino acid through a hydrogen bond, whereas, in the case of inhibitor **165l**, the nitro group seemed to interact with the embedded Ni^2+^ ion.

#### 2.9.5. GSK3α and GSK3β Inhibitors

**Scheme 29:**
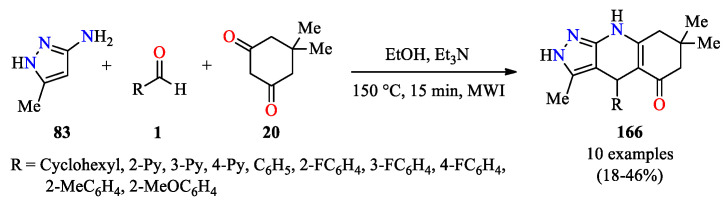
Microwave-assisted three-component synthesis of pyrazolo-tetrahydroquinolinones **166** as GSK3*α* and GSK3*β* inhibitors.

Glycogen Synthase Kinase 3 (GSK3) is a key regulator of insulin-dependent glycogen synthesis, which has been shown to function as a master regulator of multiple signaling pathways, including insulin signaling, neurotrophic factor signaling, neurotransmitter signaling, and microtubule dynamics [165,166]. In this context, Wagner et al. reported the discovery of a novel pyrazolo-tetrahydroquinolinone scaffold **166** with selective and potent GSK3 inhibition. The tricyclic compounds **166** were prepared through a three-component reaction of 5-methyl-1*H*-pyrazol-3-amine **83**, aldehydes **1**, 5,5-dimethylcyclohexane-1,3-dione **20**, and triethylamine in ethanol under microwave heating at 150 °C for 15 min (Scheme 29) [167]. The racemic compounds **166** were obtained in low to moderate yields after a simple filtration process. IC_50_ values for GSK3*α* and GSK3*β* inhibition were measured in a mobility shift microfluidic assay measuring the phosphorylation of a synthetic substrate. It was noted that the tested racemic compounds **166** inhibit GSK3*α* and GSK3*β* with IC_50_ values ranging from 0.018 to >33.33 μM and 0.051 to >33.33 μM, respectively. Afterward, the enantiomers of BRD4003 (R = C_6_H_5_) were separated by chiral HPLC and the absolute stereochemistry of each enantiomer was determined indirectly via a high-resolution *h*GSK3*β* co-crystal structure of the closely related analog. Remarkably, the (*S*)-enantiomer strongly inhibits GSK3*α* and GSK3*β* (IC_50_ 0.343 μM and 0.468 μM, respectively). In contrast, the (*R*)-enantiomer weakly inhibits GSK3*α* and GSK3*β* (IC_50_ 4.27 μM and 7.27 μM, respectively).

#### 2.9.6. Larvicidal and Insecticidal Activity

Malaria remains a major public health challenge with an estimated 229 million cases recorded in 2019 [168]. The disease spreads from one person to another via the bite of a female mosquito of the genus Anopheles [169]. There are 465 to 474 described *Anopheles* species with 70 of its members recognized to transmit the *Plasmodium* parasite to humans [170]. Therefore, the control of *Anopheles arabiensis* populations still represents the best line of defense. In this way, fused indolo-pyrazoles (FIPs) **136** were prepared via a multicomponent approach and discussed in Section 2.3. Antifungal activity (Scheme 18) [122] and were also screened for larvae mortality. It should be mentioned that *Anopheles arabiensis* mosquitoes were obtained from a colonized strain from Zimbabwe, which had been reared according to the WHO (1975) guidelines in an insectary simulating the temperature (27.5 °C), humidity (70 %), and lighting (12/12) of a malaria-endemic environment. Each container was monitored for larval mortality at 24 h intervals for three days [122]. The percentage mortality was calculated relative to the initial number of exposed larvae. Overall, seven FIPs produced more than 60% mortality at a dose of 4.0 μM. The highest activity was detected for **136c** (R = 4-Cl, R^1^ = H), **136r** (R = H, R^1^ = Me), and **136v** (R = 4-CF_3_, R^1^ = Me), which was comparable to the positive control Temephos, an effective emulsifiable organophosphate larvicidal used by the malarial control program. Additionally, the insecticidal activity assessment was conducted by exposing susceptible adult mosquitoes to a treated surface, by following WHO protocol (1975). Deltamethrin (15 g/ L, K-Othrine) was used as a positive control. The effect of FIPs **136** was measured by determining the knock-down rate, which was based on temporary paralysis of mosquitoes during a 60 min exposure period, and mortality 24 h post-exposure. Overall, the FIPs **136a** (R = R^1^ = H), **136c** (R = 4-Cl, R^1^ = H), and **136p** (R = 3-MeO-4-OH, R^1^ = C_6_H_5_) showed a 40% knock-down of activity within the first 60 min of exposure. After 24 h, the mortality of *Anopheles arabiensis* adults exposed to FIPs **136a**, **136c**, and **136p** was nearly 80%, which was comparable to the positive control K-Othrine.

## 3. Conclusions

In the present comprehensive review, a variety of MCR-based approaches applied to the synthesis of biologically active pyrazole derivatives was described. Particularly, it covered the articles published from 2015 to date related to antibacterial, anticancer, antifungal, antioxidant, *α*-glucosidase and *α*-amylase inhibitory, anti-inflammatory, antimycobacterial, and antimalarial activities, among others, of pyrazole derivatives obtained exclusively through MCRs, giving significant insight into reported MCR-based synthetic routes of pyrazole derivatives, as well as various plausible synthetic mechanisms, a comprehensive view of their diverse biological activity data, and some discussions on molecular docking studies showing how the obtained pyrazole-based compounds interacted with therapeutically relevant targets for potential pharmaceutical applications.

## Figures and Tables

**Table 1:** Four-component synthesis and antimicrobial evaluation of pyrano[2,3-*c*]pyrazole derivatives **9** and **10**.

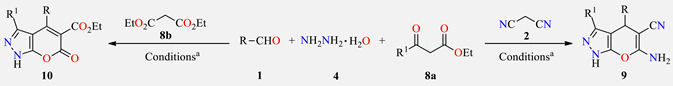										
Compound	R	R^1^	Yield 9 (%)	Yield 10 (%)	MIC (μg/mL)					
					*BS*	*CT*	*SP*	*ST*	*PA*	*VC*
**9a**	2-OHC_6_H_4_	Me	92	--	3.57	3.57	5.00	1.87	1.25	3.125
**9b**	4-MeC_6_H_4_	Me	91	--	1.25	0.10	3.125	3.57	7.50	1.87
**9c**	4-MeOC_6_H_4_	Me	91	--	3.125	7.50	2.50	6.25	1.25	1.00
**9d**	C_6_H_5_	Me	93	--	3.57	1.00	1.25	5.00	1.87	6.25
**9e**	2-Furanyl	Me	90	--	2.50	1.87	5.00	6.25	3.57	1.25
**9f**	2-OHC_6_H_4_	EtO	91	--	3.125	1.87	2.50	5.00	3.125	6.25
**9g**	4-MeC_6_H_4_	EtO	89	--	1.00	1.25	3.75	3.125	5.00	0.93
**9h**	4-MeOC_6_H_4_	EtO	90	--	0.75	1.00	1.25	3.75	2.50	5.00
**9i**	C_6_H_5_	EtO	91	--	1.00	2.50	3.75	2.50	3.125	6.25
**9j**	9-Anthracenyl	EtO	86	--	1.25	0.93	1.87	3.75	3.125	7.50
**9k**	2-Furanyl	EtO	91	--	0.10	1.00	0.10	0.50	0.75	2.50
**10a**	2-OHC_6_H_4_	Me	--	89	1.87	0.93	1.25	3.125	1.87	5.00
**10b**	4-MeC_6_H_4_	Me	--	85	1.87	1.00	2.50	5.00	3.57	6.25
**10c**	4-MeOC_6_H_4_	Me	--	86	1.87	3.125	6.25	1.87	6.25	7.50
**10d**	C_6_H_5_	Me	--	88	3.125	1.00	2.50	3.75	1.25	5.00
**10e**	2-Furanyl	Me	--	86	0.75	0.10	1.25	1.87	2.50	3.125
Ciprofloxacin ^b^	--	--	--	--	6.25	6.25	3.125	3.125	3.125	3.125

^a^ Reaction conditions: (hetero)aromatic aldehyde **1** (1 mmol), hydrazine hydrate **4** (1 mmol), ethyl acetoacetate **8a** or diethyl malonate **8b** (1 mmol), malononitrile **2** or diethyl malonate **8b** (1 mmol), and piperidine (5 mol%) at room temperature for 20 min. ^b^ Positive control for the study. *BS* (*Bacillus subtilis*), *CT* (*Clostridium tetani*), *SP* (*Streptococcus pneumoniae*), *ST* (*Salmonella typhi*), *PA* (*Pseudomonas aeruginosa*), *VC* (*Vibrio cholerae*).

**Table 2:** Solvent-free five-component synthesis of pyrano[2,3-*c*]pyrazoles **12** as antimicrobial agents.

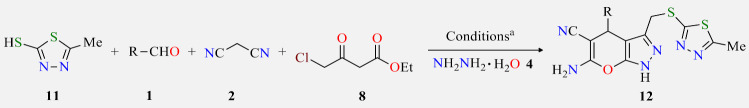										
Compound	R	Yield 12 (%)	Zone of Inhibition (mm)							
			*SA*		*BS*		*PV*		*EC*	
			50 μg/Well	100 μg/Well	50 μg/Well	100 μg/Well	50 μg/Well	100 μg/Well	50 μg/Well	100 μg/Well
**12a**	C_6_H_5_	85	11	13	13	15	16	20	20	24
**12b**	2-MeOC_6_H_4_	81	3	6	5	6	6	9	10	12
**12c**	3-OHC_6_H_4_	86	6	9	8	9	10	13	12	16
**12d**	4-ClC_6_H_4_	91	15	16	18	21	22	26	27	31
**12e**	2-OHC_6_H_4_	82	8	10	9	11	11	15	14	18
**12f**	4-NO_2_C_6_H_4_	90	16	19	21	24	25	28	31	34
**12g**	4-FC_6_H_4_	87	16	18	19	22	23	27	29	33
**12h**	4-OHC_6_H_4_	89	9	10	10	12	13	18	17	21
**12i**	4-MeOC_6_H_4_	84	5	7	6	7	8	11	11	13
**12j**	2-MeC_6_H_4_	82	0	0	0	0	0	0	0	0
**12k**	4-MeC_6_H_4_	86	0	0	0	0	0	0	0	0
**12l**	2-ClC_6_H_4_	83	14	16	17	19	20	24	25	29
**12m**	3-FC_6_H_4_	88	13	15	14	16	17	23	22	26
**12n**	4-BrC_6_H_4_	85	10	12	11	13	14	19	19	23
Ciprofloxacin ^b^	--	--	21	24	23	26	31	33	32	36

^a^ Reaction conditions: 2-thiolyl-5-methyl-1,3,4-thiadiazole **11** (1.1 mmol), aldehyde **1** (1 mmol), malononitrile **2** (1 mmol), ethyl 4-chloro-3-oxobutanoate **8** (1 mmol), hydrazine hydrate **4** (1 mmol), and montmorillonite K10, 65–70 °C, 5 h. ^b^ Positive control for the study. *SA* (*Staphylococcus aureus*), *BS* (*Bacillus subtilis*), *PV* (*Proteus vulgaris*), *EC* (*Escherichia coli*).

**Table 3:** Ultrasound-assisted four-component synthesis of pyrano[2,3-*c*]pyrazoles **18** as antimicrobial agents.

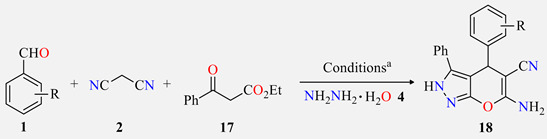						
Compound	R	Yield 18 (%)	MIC (μg/mL)			
			*EC*	*PA*	*SS*	*SA*
**18a**	H	94	20	30	25	20
**18b**	4-Me	85	25	35	45	25
**18c**	3-NO_2_	89	30	45	45	40
**18d**	3-OH	84	30	30	30	30
**18e**	2-Me	87	25	40	35	35
**18f**	2-NO_2_	92	35	45	35	20
**18g**	4-MeO	85	35	30	25	35
**18h**	4-Br	93	40	25	45	45
**18i**	4-*i*Pr	86	45	40	35	40
**18j**	2-Cl	90	35	30	45	35
Cefazolin ^b^	--	--	>35	>35	>35	>35

^a^ Reaction conditions: Aldehyde **1** (1 mmol), malononitrile **2** (1 mmol), ethyl 3-oxo-3-phenylpropanoate **17** (1 mmol), hydrazine hydrate **4** (1.5 mmol), and graphene oxide (10 mol%) in H_2_O (5 mL) at r.t for 2–6 min under ultrasound irradiation. ^b^ Positive control for the study. *EC* (*Escherichia coli*), *PA* (*Pseudomonas aeruginosa*), *SS* (*Staphylococcus saprophyticus*), *SA* (*Staphylococcus aureus*).

**Table 4:** Four-component synthesis and antibacterial evaluation of pyrazole-dimedone derivatives **21**.

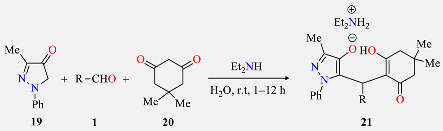								
Compound	R	Yield 21 (%)	*SA*		*EF*		*BS*	
			CPM (mm)	MIC (μg/L)	CPM (mm)	MIC (μg/L)	CPM (mm)	MIC (μg/L)
**21a**	2,4-diClC_6_H_3_	78	13	32	14	32	12	32
**21b**	C_6_H_5_	62	15	32	13	16	15	16
**21c**	4-ClC_6_H_4_	50	13	32	24	32	16	32
**21d**	4-MeC_6_H_4_	62	16	32	16	32	18	32
**21e**	3-MeC_6_H_4_	66	19	16	15	32	14	64
**21f**	4-BrC_6_H_4_	71	14	32	13	64	15	32
**21g**	3-BrC_6_H_4_	70	14	32	15	32	16	32
**21h**	4-NO_2_C_6_H_4_	52	12	64	14	32	16	32
**21i**	3-NO_2_C_6_H_4_	63	14	32	12	64	17	32
**21j**	4-MeOC_6_H_4_	64	10	64	13	32	10	32
**21k**	4-FC_6_H_4_	57	13	32	13	32	20	8
**21l**	4-CF_3_C_6_H_4_	76	16	16	16	32	16	32
**21m**	2,6-diClC_6_H_3_	40	15	32	13	32	12	32
**21n**	2-Naphthyl	76	14	32	13	32	15	32
**21o**	2-Thienyl	75	13	32	20	32	15	16
Ciprofloxacin ^a^	--	--	27	≤0.25	24	≤0.25	25	≤0.25

^a^ Positive control for the study. *SA* (*Staphylococcus aureus*), *EF* (*Enterococcus faecalis*), *BS* (Bacillus subtilis).

**Table 5:** One-pot three-component synthesis and antibacterial activity of coumarin substituted thiazolyl-3-aryl-pyrazole-4-carbaldehydes **25**.

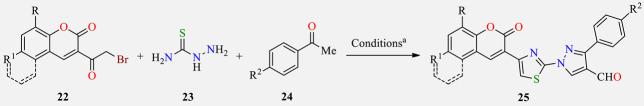										
Compound	R	R^1^	R^2^	Yield 25 (%)	MIC (μg/mL)					
					*EC*	*KP*	*PV*	*MRSA*	*BS*	*BC*
**25a**	H	H	H	84	135	150	133	150	132	145
**25b**	H	Cl	H	80	134.6	129	130	150	129.5	138.6
**25c**	Cl	Cl	H	75	119.6	118	131.5	115	123	117
**25d**	H	Br	H	80	118.1	116.8	124	116	125	129
**25e**	Br	Br	H	85	115.6	119	120	116.8	118	119
**25f**	H	Benzo	H	78	132.8	150	150	135	122.8	138.9
**25g**	H	Cl	Cl	77	126.3	117.4	130.7	134	112.2	110.8
**25h**	Cl	Cl	Cl	73	110.9	105	113	112.5	110.7	112
**25i**	H	Br	Cl	83	117.5	115	127.5	122.9	119.7	115
**25j**	MeO	H	Cl	82	125.3	129	132.3	139	132.6	126
**25k**	H	H	Me	77	132.5	150	150	150	150	135
**25l**	H	Br	Me	82	134.6	124	121.8	138.1	125	126
**25m**	Cl	Cl	Me	81	101.3	100.9	105	115.6	98.2	100.1
**25n**	Br	Br	Me	85	86.5	79.1	100.7	105.9	92.4	72.8
**25o**	MeO	H	Me	82	132.7	150	142	150	150	136
Gentamycin ^b^	--	--	--	--	2	4	4	22	45	10
Ampicillin ^b^	--	--	--	--	10	-- ^c^	-- ^c^	4	5	4

^a^ Reaction conditions: 3-(2-bromoacetyl)coumarin derivative **22** (1 mmol), thiosemicarbazide **23** (1 mmol), substituted acetophenone **24** (1 mmol), DMF (5 mL), POCl_3_ (5 mmol), 0–60 °C, 5–6 h. ^b^ Positive control for the study. ^c^ Not determined. *EC* (*Escherichia coli*), *KP* (*Klebsiella pneumoniae*), *PV* (*Proteus vulgaris*), *MRSA* (*Methicillin-resistant Staphylococcus aureus*), *BS* (*Bacillus subtilis*), and *BC* (*Bacillus cereus*).

**Table 6:** Three-component synthesis and antibacterial activity of spiropyrrolidine-oxindoles **29**.

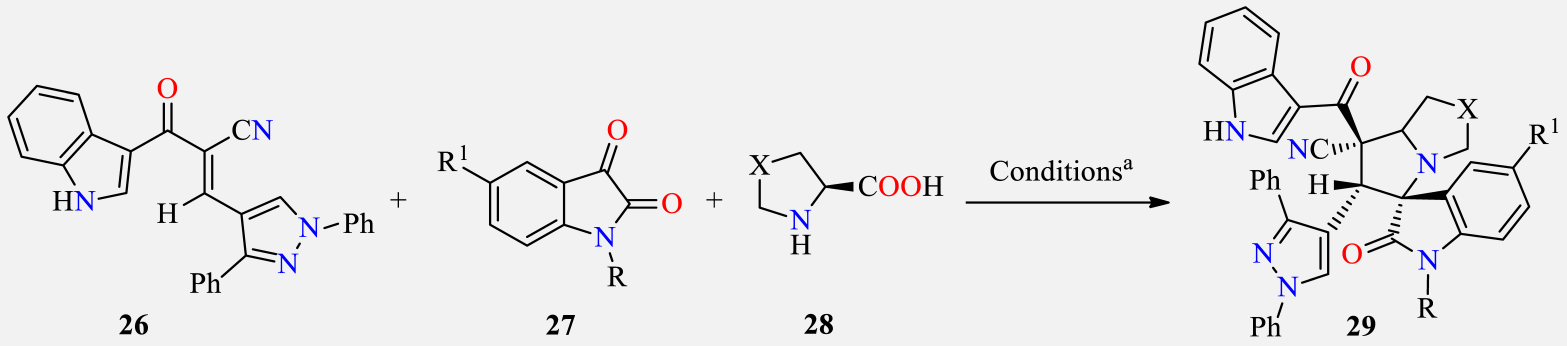													
Compound	R	R^1^	X	Yield 29 (%)	MIC (μg/mL)								
					*ST*	*KP*	*PV*	*SF*	*ML*	*EA*	*SE*	*SA*	*MRSA*
**29a**	H	H	CH_2_	92	250	31.2	125	31.2	31.2	62.5	62.5	31.2	31.2
**29b**	Allyl	H	CH_2_	86	500	500	250	250	250	250	125	125	250
**29c**	*n*-Butyl	H	CH_2_	90	125	250	250	-- ^c^	500	500	62.5	125	500
**29d**	Me	H	CH_2_	93	500	500	250	250	500	250	250	250	125
**29e**	Propargyl	H	CH_2_	87	125	250	250	500	500	250	125	500	250
**29f**	H	NO_2_	CH_2_	80	250	250	125	500	500	250	250	125	500
**29g**	H	H	S	91	500	500	500	500	500	500	500	500	500
**29h**	Allyl	H	S	88	250	500	500	500	500	250	125	250	500
**29i**	Benzyl	H	S	86	250	250	500	500	250	250	125	62.5	500
**29j**	*n*-Butyl	H	S	93	250	500	500	500	250	500	250	250	500
**29k**	Me	H	S	89	-- ^c^	500	500	500	^-- c^	250	500	125	500
Streptomycin ^b^	--	--	--	--	30	6.25	-- ^c^	6.25	6.25	25	6.25	6.25	6.25

^a^ Reaction conditions: (*E*)-3-(1,3-diphenyl-1*H*-pyrazol-4-yl)-2-(1*H*-indole-3-carbonyl)acrylonitrile **26** (1 mmol), isatin derivative **27** (1 mmol), and amino acid **28** (1.1 mmol), MeOH (5 mL), reflux, 3 h. ^b^ Positive control for the study. ^c^ Not determined. *ST* (*Salmonella typhimurium*), *KP* (*Klebsiella pneumoniae*), *PV* (*Proteus vulgaris*), *SF* (*Shigella flexneri*), *ML* (*Micrococcus luteus*), *EA* (*Enterobacter aerogenes*), *SE* (*Staphylococcus epidermidis*), *SA* (*Staphylococcus aureus*), *MRSA* (*Methicillin-resistant Staphylococcus aureus*).

**Table 7:** One-pot three-component synthesis and antibacterial activity of 4-[(3-aryl-1-phenyl-1*H*-pyrazol-4-yl)methylidene]-2,4-dihydro-3*H-*pyrazol-3-ones **34**.

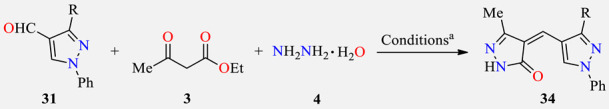						
Compound	R	Yield 34 (%)	Zone of Inhibition (mm)			
			*SA*	*BS*	*PA*	*EC*
**34a**	C_6_H_5_	84	15.8	12.0	13.5	14.3
**34b**	4-FC_6_H_4_	85	17.2	11.6	12.0	11.5
**34c**	4-ClC_6_H_4_	88	14.0	8.9	10.9	10.8
**34d**	4-BrC_6_H_4_	88	14.1	8.0	11.2	10.5
**34e**	4-MeC_6_H_4_	90	9.0	8.4	9.6	10.2
**34f**	4-MeOC_6_H_4_	92	10.0	7.9	8.8	9.2
**34g**	3-MeOC_6_H_4_	85	12.2	9.4	12.0	8.0
**34h**	4-OHC_6_H_4_	82	9.6	11.0	9.7	9.7
**34i**	4-NO_2_C_6_H_4_	82	14.8	8.8	10.3	11.8
**34j**	2,4-(Cl)_2_C_6_H_3_	86	15.6	10.8	12.1	12.0
Norfloxacin ^b^	--	--	25.6	19.2	24.2	24.0

^a^ Reaction conditions: 3-aryl-1-phenyl-1*H*-pyrazole-4-carbaldehyde **31** (1 mmol), ethyl acetoacetate **3** (1 mmol), hydrazine hydrate **4** (1 mmol), and sodium acetate (2 mmol), EtOH (5 mL), reflux, 1 h. ^b^ Positive control for the study. *SA* (*Staphylococcus aureus*), *BS* (*Bacillus subtilis*), *PA* (*Pseudomonas aeruginosa*), *EC* (*Escherichia coli*).

**Table 8:** One-pot three-component synthesis of 3-alkyl-1-(4-(aryl/heteroaryl)thiazol-2- yl)indeno[1,2-*c*]pyrazol-4(1*H*)-ones **37** as antibacterial agents.

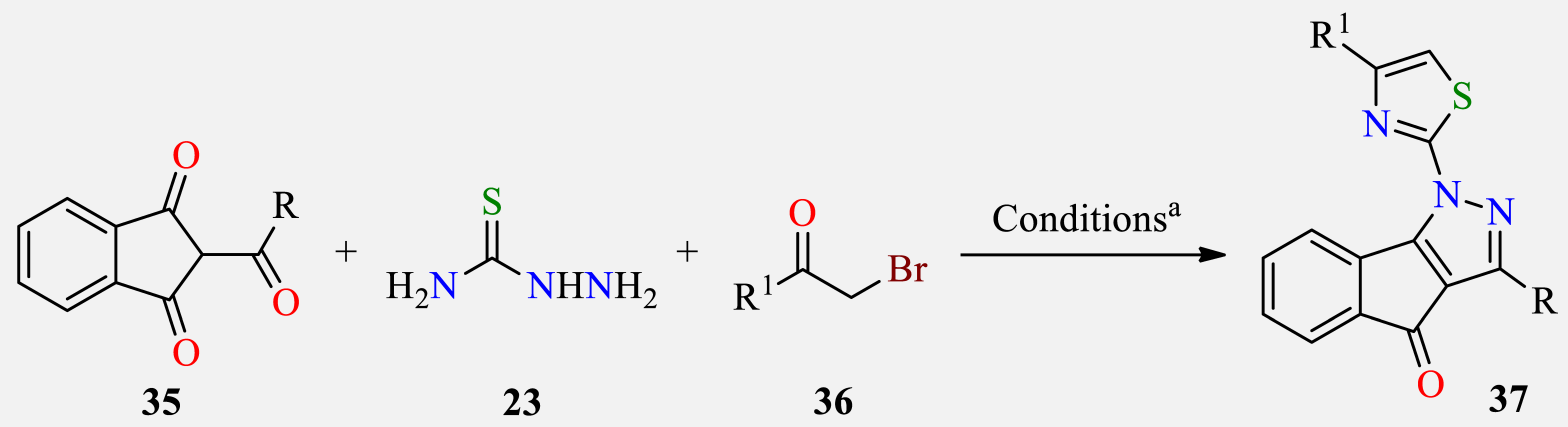							
Compound	R	R^1^	Yield 37 (%)	MIC (μmol/mL)			
				*SA*	*BS*	*PA*	*EC*
**37a**	Me	Biphenyl	74	0.0297	0.0595	0.0297	0.0297
**37b**	Et	Biphenyl	78	0.0288	0.0576	0.0288	0.0288
**37c**	*i*Pr	Biphenyl	72	0.0559	0.0559	0.0559	0.0559
**37d**	*i*Bu	Biphenyl	80	0.0270	0.0541	0.0270	0.0270
**37e**	Me	2-Naphthyl	72	0.0317	0.0635	0.0317	0.0317
**37f**	Et	2-Naphthyl	75	0.0613	0.0613	0.0306	0.0613
**37g**	*i*Pr	2-Naphthyl	79	0.0593	0.0593	0.0593	0.0593
**37h**	*i*Bu	2-Naphthyl	76	0.0287	0.0574	0.0287	0.0287
**37i**	Me	2-Benzofuranyl	63	0.0326	0.0652	0.0326	0.0326
**37j**	Et	2-Benzofuranyl	60	0.0629	0.0629	0.0629	0.0629
**37k**	*i*Pr	2-Benzofuranyl	58	0.0607	0.0607	0.0607	0.0607
**37l**	*i*Bu	2-Benzofuranyl	53	0.0293	0.0587	0.0293	0.0293
Ciprofloxacin ^b^	--	--	--	0.0094	0.0094	0.0094	0.0094

^a^ Reaction conditions: 2-acyl-(1*H*)-indene-1,3-(2*H*)dione **35** (1 mmol), thiosemicarbazide **23** (1 mmol), MeOH (30 mL), reflux, 10–15 min, then *α*-bromoketone **36** (5 mmol), sodium acetate (5 mmol), MeOH/AcOH (2:1, *v/v*) (20 mL), reflux, 5–8 h. ^b^ Positive control for the study. *SA* (*Staphylococcus aureus*), *BS* (*Bacillus subtilis*), *PA* (*Pseudomonas aeruginosa*), *EC* (*Escherichia coli*).

**Table 9:** Schematic route for the multicomponent synthesis of pyrazole-based Ugi products **41** and their in vitro antibacterial activities (MIC) against five bacterial strains.

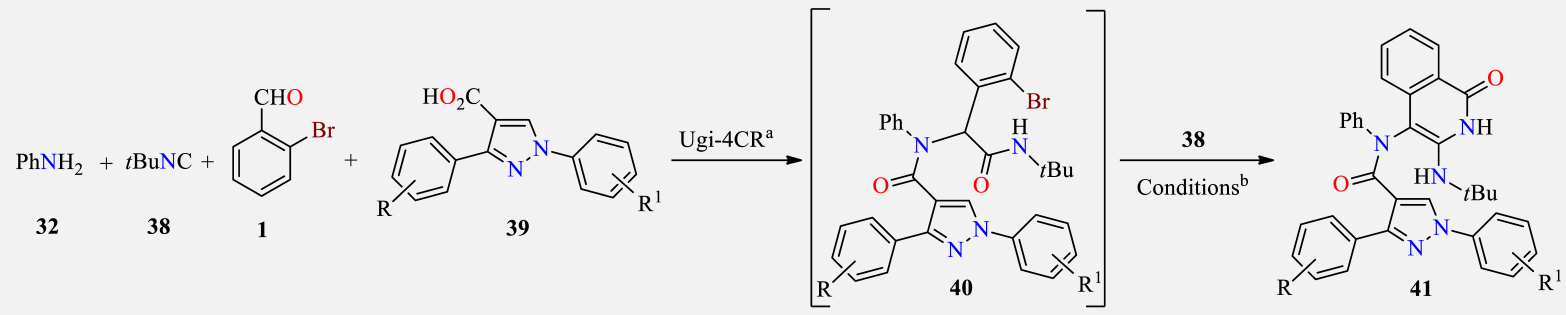								
Compound	R	R^1^	Yield of 41 (%)	Antibacterial Activity of the Synthesized Compounds 41 (in μM)				
				*SA*	*EC*	*EF*	*EP*	*VC*
**41a**	H	H	91	>250	>250	>250	>250	>250
**41b**	4-Br	H	51	>250	>250	>250	>250	>250
**41c**	4-Me	H	76	>250	>250	>250	>250	>250
**41d**	H	4-F	88	>250	>250	>250	>250	>250
**41e**	H	4-NO_2_	79	20.85	31.5	>250	200	41.25
**41f**	H	4-Cl	87	90.5	100	200	>250	125
**41g**	H	3,5-diCl	81	37.25	37.25	>250	>250	90.5
Kanamycin ^c^	--	--	--	31.3	3.9	62.5	62.5	62.5

^a^ Reaction conditions: **32** (1 mmol), **38** (1 mmol), **1** (1 mmol), **39** (1.2 mmol) in MeOH (3 mL), 35–40 C, 15–18 h, 77–96%. ^b^ Reaction conditions: **40a** (1 mmol), *t-*butyl isocyanide **38** (1.2 mmol), catalyst Pd(OAc)_2_ (10 mol%), base Cs_2_CO_3_ (2 mmol), solvent DMF (2.5 mL), MWI (150 °C), 15 min under inert (N_2_) conditions. *SA* (*Staphylococcus aureus*), *EC* (*Escherichia coli*), *EF* (*Enterococcus faecalis*), *EP* (*Streptococcus pyogens*), *VC* (*Vibrio cholera*). ^c^ Positive control for the study, commonly used antibacterial drug.

**Table 10:** Multicomponent synthesis and antimicrobial activity of spiroindenopyridazine-4*H*-pyrane derivatives **45**.

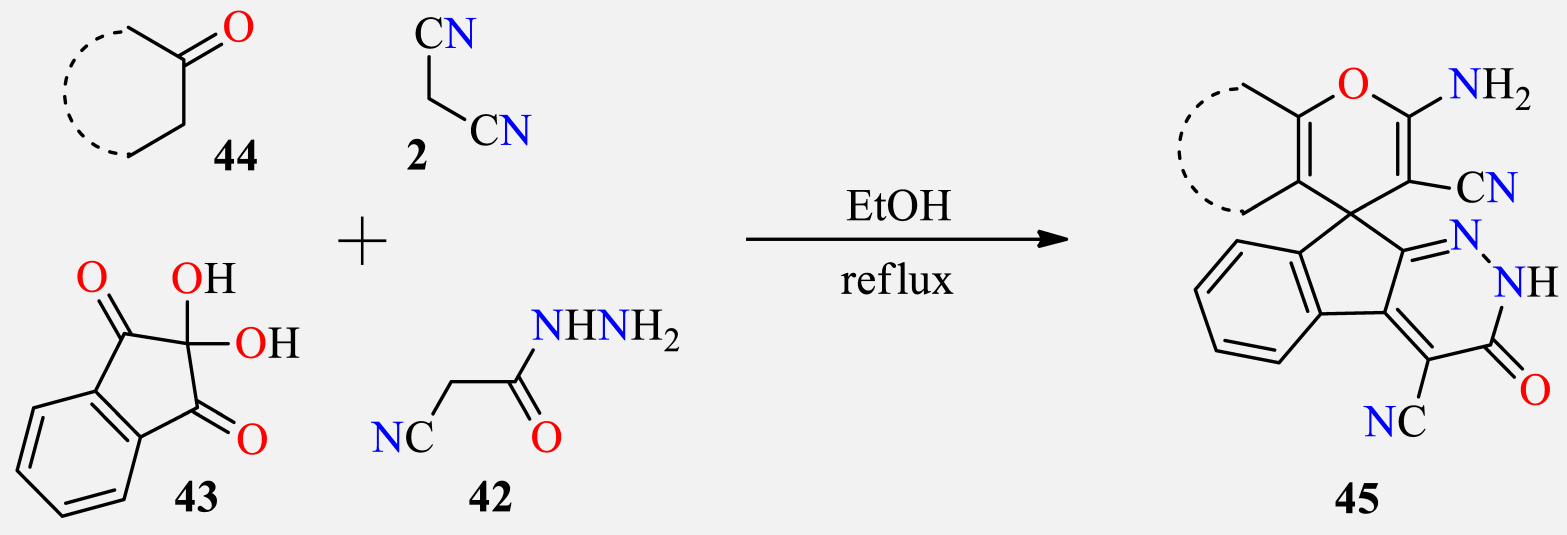					
Compound	Reagent 44	Yield of 45 (%)	Antibacterial Activity of the Synthesized Compounds 45 (in μM)		
			MIC’S (mM) *SA*	Inhibition Zone (mm) *SA*	Inhibition Zone (mm) *EC*
**45a**	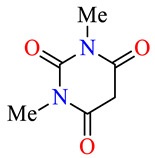	95	5	7	--- ^a^
**45b**	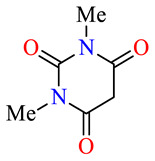	97	5	5	---
**45c**	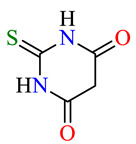	93	50	7	---
**45d**	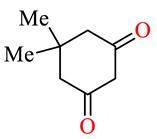	98	---	---	---
**45e**	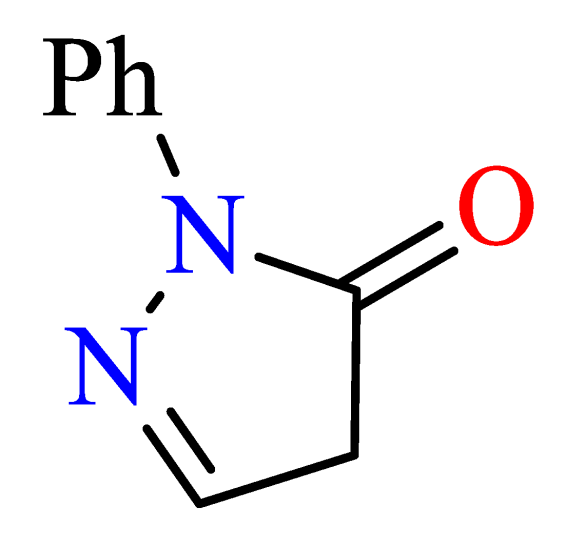	97	10	9	---
**45f**	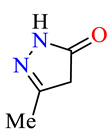	93	5	5	---
**45g**	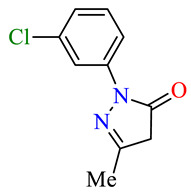	98	5	15	---
**45h**	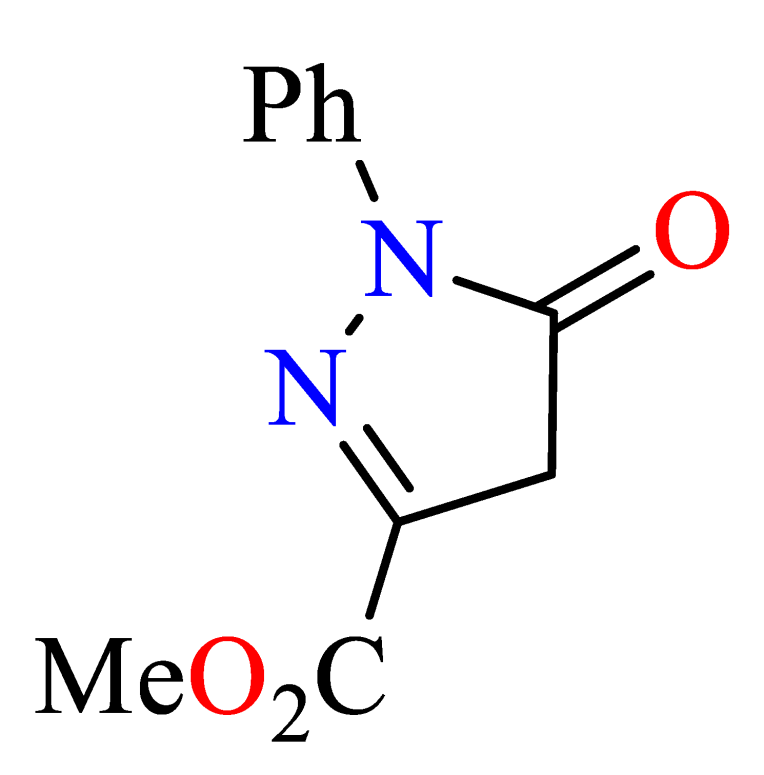	96	---	---	---
**45i**	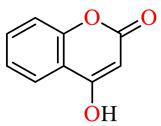	90	5	4	---
**45j**	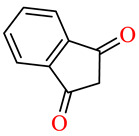	92	---	---	---
Tetracycline ^b^	---	---	---	30	23
DMSO	---	---	---	0	0

^a^ inactive at concentration 50 mM. ^b^ Positive control for the study. *SA* (*Staphylococcus aureus*), *EC* (*Escherichia coli*).

**Table 11:** Multicomponent synthesis and their in vitro antibacterial activity of pyranopyrazole derivatives **46** using ChCl•2 thiourea and ChCl•2 thiourea as catalysts, under both ultrasonic irradiation and reflux conditions.

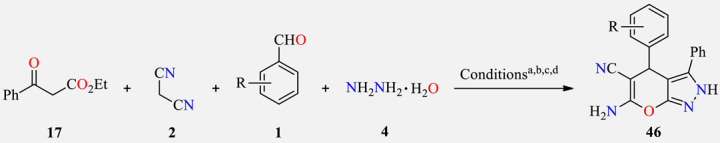						
Compound	R	Yield of 46 (%)	Antibacterial Activity of the Synthesized Compounds 46 (MIC in μg•mL^·1^)			
			*EC*	*PA*	*SS*	*SA*
**46a**	H	(98) ^a^, (95)^c^	25	20	30	35
**46b**	4-Me	(94) ^a^, (80) ^b^, (90) ^c^, (76) ^d^	30	40	35	30
**46c**	3-NO_2_	(93) ^a^, (83) ^b^, (92) ^c^, (80) ^d^	30	40	25	40
**46d**	3-OH	(94) ^a^, (89) ^b^	35	20	35	35
**46e**	2-Me	(97) ^a^	35	20	30	40
**46f**	4-OH	(97) ^a^, (88) ^b^	25	40	30	25
**46g**	2-NO_2_	(93) ^a^	30	20	35	30
**46h**	2-OH	(88) ^a^, (69) ^b^	40	20	40	35
**46i**	4-OMe	(98) ^a^, (73) ^b^	40	45	30	30
**46j**	4-Cl	(91) ^a^, (80) ^b^, (90) ^c^, (82) ^d^	30	35	35	35
**46k**	4-Br	(89) ^a^	35	30	30	30
**46l**	4-N(Me)_2_	(96) ^a^, (75) ^b^	30	25	25	35
**46m**	4-*i*Pr	(89) ^a^, (80) ^b^	30	45	35	35
**46n**	4-NO_2_	(91) ^a^, (89) ^b^	30	45	15	35
**46o**	2-Cl	(89) ^a^	25	30	35	15
**46p**	3-Br	(96) ^a^	15	20	35	25
Cefazolin ^e^	--	--	>35	>35	>35	>35

^a^ Reaction conditions: ^a^ (EtOH, reflux at 80 °C, catalyst ChCl•2 thiourea 10 mol%), ^b^ (EtOH, reflux at 80 °C, catalyst ChCl•2 urea 10 mol%), ^c^ (sonication at rt, catalyst ChCl•2 thiourea 10 mol%), ^d^ (sonication at rt, catalyst ChCl•2 urea 10 mol%). *EC* (*Escherichia coli*), *PA* (*Pseudomonas aeruginosa*), *SS* (*Staphylococcus saprophyticus*), *SA* (*Staphylococcus aureus*). ^e^ Cefazolin is taken as a standard drug for this study.

**Table 12:** Pseudo three-component synthesis and antibacterial evaluation of pyrazolylbiscoumarins **47** and pyrazolylxanthenediones **48**.

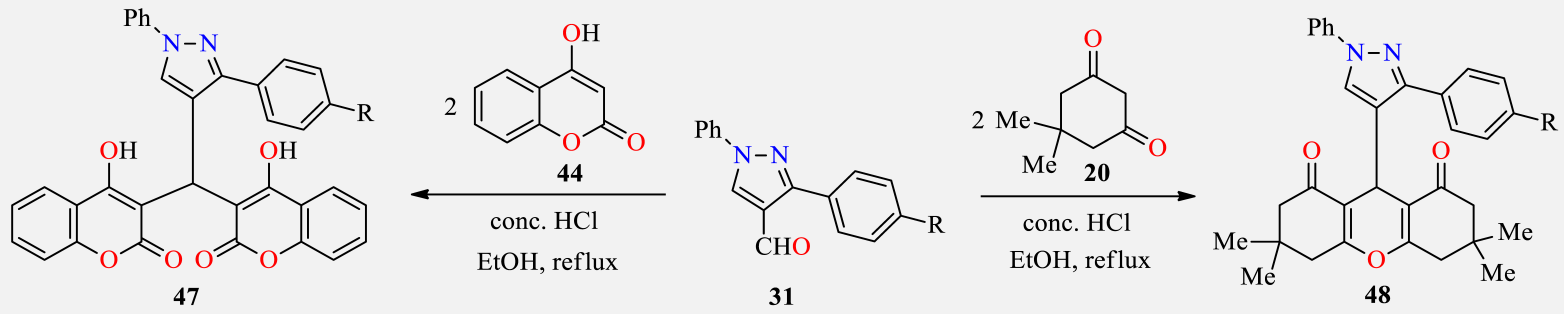										
Compound	R	Yield of 47/48 (%)	Activity (Zone of Inhibition in mm) at Various Concentrations in ppm							
			*SA*				*KP*			
			50	100	150	250	50	100	150	250
**47a**	H	80	11	13	15	19	13	14	16	17
**47b**	Me	82	7	9	11	12	9	11	12	14
**47c**	OMe	84	--	8	10	14	6	9	12	15
**47d**	Br	86	6	8	11	13	8	11	13	16
**47e**	NO_2_	82	10	13	14	17	11	12	14	16
**48a**	H	83	13	17	18	19	16	18	20	22
**48b**	Me	82	8	11	13	14	9	11	14	17
**48c**	OMe	86	--	7	9	12	--	8	9	11
**48d**	Br	80	6	8	10	11	7	9	10	12
**48e**	NO_2_	84	--	7	9	13	--	8	12	14
Streptomycin ^a^	--	--	21				25			

*SA (Staphylococcus aureus)*, *KP (Klebsiella pneumoniae)*. ^a^ Positive control for the study.

**Table 13:** Three-component synthesis of pyrazolo-quinolines **51** and their in vitro antibacterial activity (MIC) against six bacterial strains.

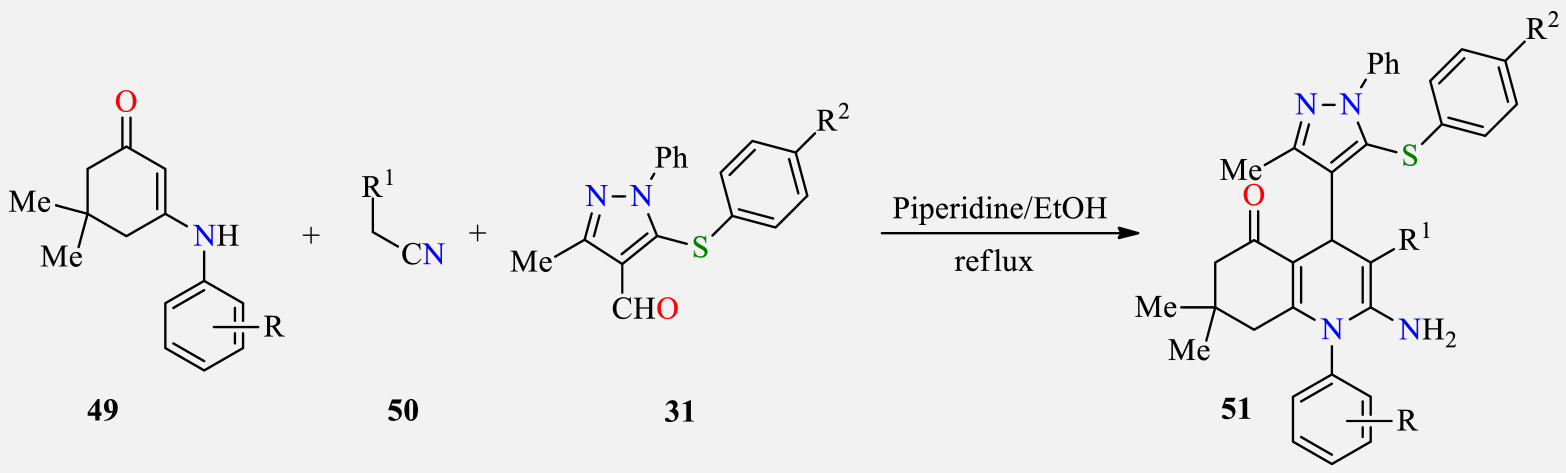										
Compound	R	R^1^	R^2^	Yield of 51 (%)	Antibacterial Activity of Compounds 51 (MIC in μg/mL)					
					*SP*	*BS*	*CT*	*EC*	*ST*	*VC*
**51a**	4-F	CN	Cl	81	500	500	250	100	250	250
**51b**	4-F	CO_2_Et	Cl	79	100	500	62.5	200	500	200
**51c**	4-F	CONH_2_	Cl	73	200	200	500	250	200	200
**51d**	4-F	CN	Me	84	62.5	250	125	200	200	1000
**51e**	4-F	CO_2_Et	Me	76	500	250	1000	250	250	1000
**51f**	4-CF_3_	CN	Me	72	500	500	500	250	100	62.5
**51g**	4-CF_3_	CO_2_Et	Me	80	100	100	500	500	200	500
**51h**	2,4-F	CN	Me	79	100	62.5	100	100	500	500
**51i**	2,4-F	CO_2_Et	Me	73	200	500	250	500	100	100
**51j**	2,4-F	CN	Cl	75	500	500	200	100	200	200
**51k**	2,4-F	CO_2_Et	Cl	78	100	1000	250	200	200	100
**51l**	2,4-F	CONH_2_	Cl	81	500	500	200	500	500	500
**51m**	4-F	CN	F	83	200	100	200	62.5	100	500
**51n**	4-F	CO_2_Et	F	71	500	100	500	100	200	500
**51o**	CF_3_	CN	F	72	200	200	62.5	62.5	100	100
**51p**	CF_3_	CO_2_Et	F	77	500	1000	200	500	500	100
Ampicillin ^a^	--	--	--	--	100	250	250	100	100	100
Norfloxacin ^a^	--	--	--	--	10	100	50	10	10	10
Chloramphenicol ^a^	--	--	--	--	50	50	50	50	50	50
Ciprofloxacin ^a^	--	--	--	--	25	50	100	25	25	25

*SP (Streptococcus pneumoniae)*, *BS (Bacillus subtilis)*, *CT (Clostridium tetani)*, *EC (Escherichia coli)*, *ST (Salmonella typhi)*, *VC (Vibrio cholerae)*. ^a^ Positive control for the study.

**Table 14:** Multicomponent synthesis of pyrimidine-, dihydropyridine-, and imidazole-based pyrazoles **52**, **53**, and **54** and their in vitro antibacterial activity.

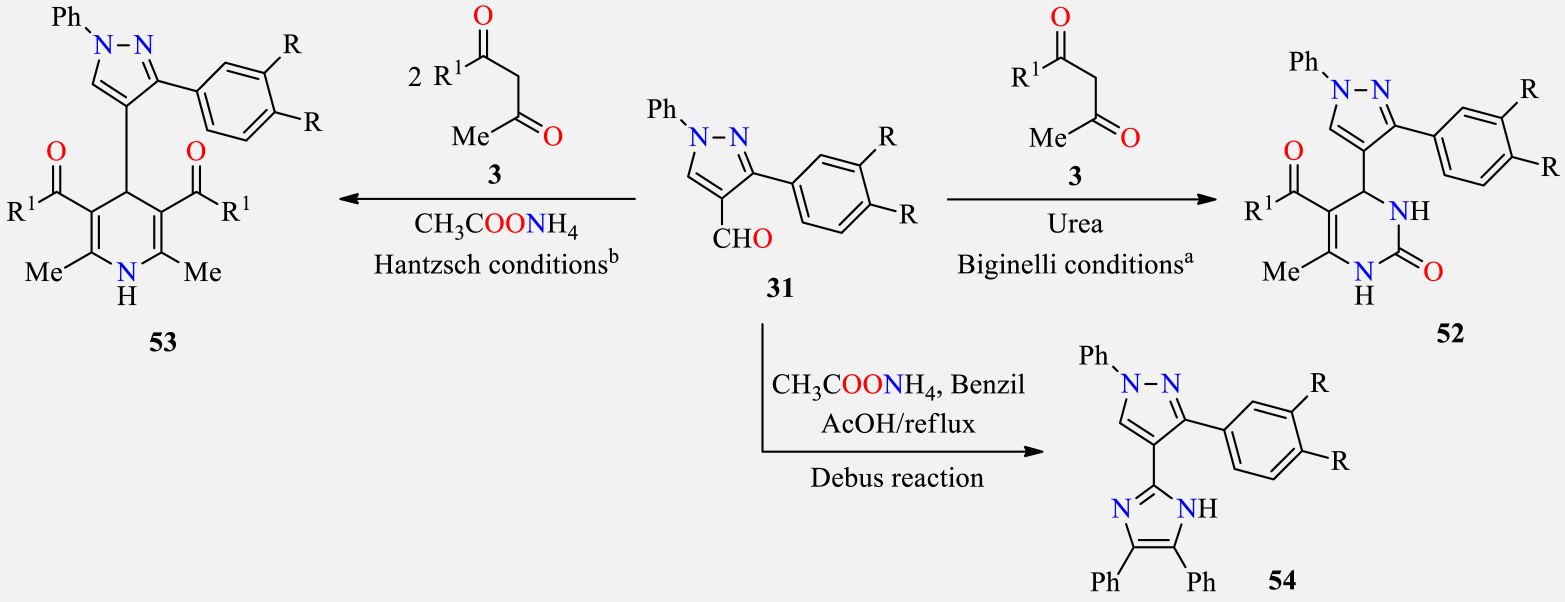							
Compound	R	R^1^	Yield of 52, 53, 54 (%)	Antibacterial Activity of the Synthesized Compounds 52, 53, 54(MIC in μg·mL^−1^) ^c^			
				*SA*	*EC*	*PA*	*KP*
**52a**	Cl	OEt	90	25 (13)	25 (14)	NP	50 (9)
**52b**	Cl	Ome	88	12.5 (15)	12.5 (15)	25 (10)	25 (11)
**52c**	Cl	Me	77	6.25 (18)	6.25 (18)	6.25 (19)	12.5 (12)
**52d**	F	Oet	89	12.5 (12)	12.5 (14)0	NP	12.5 (11)
**52e**	F	Ome	80	25 (12)	12.5 (13)	50 (10)	25 (11)
**52f**	F	Me	72	12.5 (16)	6.25 (16)	6.25 (17)	6.25 (19)
**53a**	Cl	Oet	74	12.5 (14)	12.5 (14)	25 (11)	25 (12)
**53b**	Cl	Ome	79	50 (9)	25 (11)	NP	100 (4)
**53c**	Cl	Me	64	50 (10)	50 (11)	50 (9)	NP
**53d**	F	Oet	72	25 (11)	25 (10)	NP	12.5 (12)
**53e**	F	Ome	65	12.5 (13)	12.5 (15)	25 (10)	12.5 (15)
**53f**	F	Me	62	50 (7)	50 (8)	100 (8)	NP
**54a**	Cl	--	86	NP	25 (12)	12.5 (10)	NP
**54b**	F	--	78	50 (10)	25 (11)	25 (9)	100 (5)
Streptomycin ^d^	--		--	6.25 (20)	6.25 (17)	6.25 (19)	6.25 (18)

^a^ Biginelli reaction conditions: A mixture of compound **31** (2 mol), type keto-derivative **3** (2.2 mol), urea (3 mol), and HCl (0.5 mL) in ethanol was heated to reflux for 6 h. ^b^ Hantzsch reaction conditions: A mixture of compound **31** (1.0 mol), type keto-derivative **3** (2 mol), and ammonium acetate (1.1 mol) in ethanol (20 mL) was refluxed for 8 h. ^c^ Values in brackets correspond to zone of inhibition in mm. *SA* (*Staphylococcus aureus*), *EC* (*Escherichia coli*), *PA* (*Pseudomonas aeruginosa*), *KP* (*Klebsiella pneumonia*). ^d^ Positive control for the study.

**Table 15:** Three-component synthesis of thiadiazole-1*H*-pyrazol-4-yl-thiazolidin-4-ones **57** and their in vitro antibacterial activity (MBC) against six bacterial strains.

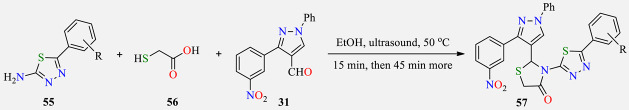								
Compound	R	Yield of 57 (%) ^a^	Antibacterial Activity of Compounds 57 (MBC in μg/mL)					
			*MRSA*	*SA*	*EC*	*PA*	*ST*	*KP*
**57a**	H	96 (74)	1250	625	2500	2500	625	625
**57b**	4-F	94 (62)	625	625	625	312.5	625	625
**57c**	4-Br	95 (56)	625	312.5	312.5	312.5	625	625
**57d**	3-F	91 (55)	1250	78.5	312.5	2500	312.5	625
**57e**	3-Cl	94 (58)	1250	625	312.5	1250	9.8	625
**57f**	3-Br	93 (60)	156.3	625	156.3	312.5	625	625
**57g**	4-NO_2_	97 (64)	625	39.1	4.9	156.3	312.5	500
**57h**	3-NO_2_	92 (56)	312.5	156.3	39.1	625	625	156.3
**57i**	4-Me	96 (57)	2500	2500	1250	1250	2500	1250
**57j**	4-OMe	95 (61)	1250	625	1250	1250	1250	1250
**57k**	3,4,5-OMe	91 (62)	1250	625	1250	2500	625	625
**57l**	3,4-OMe	93 (60)	2500	625	625	2500	625	1250
Ciprofloxacin ^b^	--	--	625	39.1	1.22	625	1.22	4.9

^a^ Yields in brackets were obtained under conventional heating at 50 °C. *MRSA* (*Methicillin-resistant Staphylococcus aureus*), *SA* (*Staphylococcus aureus*), *EC* (*Escherichia coli*), *PA* (*Pseudomonas aeruginosa*), *ST* (*Salmonella typhimurium*), *KP* (*Klebsiella pneumonia*). ^b^ Positive control for the study.

**Table 16:** Microwave-assisted three-component synthesis of pyrano-pyrazole derivatives **59** and their in vitro antibacterial activity evaluation against a panel of six pathogenic strains.

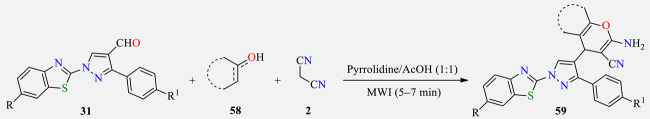										
Compound	Reagent 58	R	R^1^	Yield of 59 (%)	Antibacterial Activity of Compounds 59 (MIC in μg/mL)					
					*BS*	*CT*	*SA*	*EC*	*ST*	*VC*
**59a**	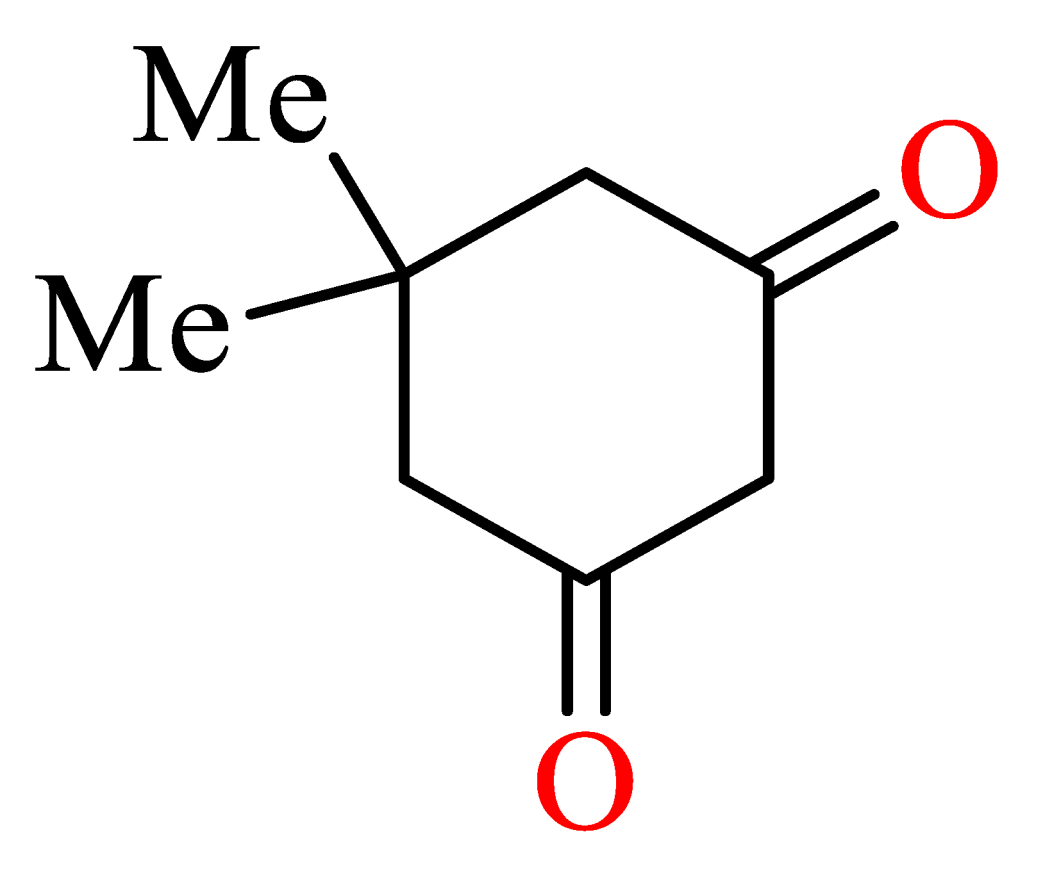	H	F	71	250	250	500	200	200	250
**59b**		H	Me	83	250	100	250	500	500	250
**59c**		Me	F	78	250	100	125	200	250	200
**59d**		Me	Me	79	500	500	500	200	250	500
**59e**	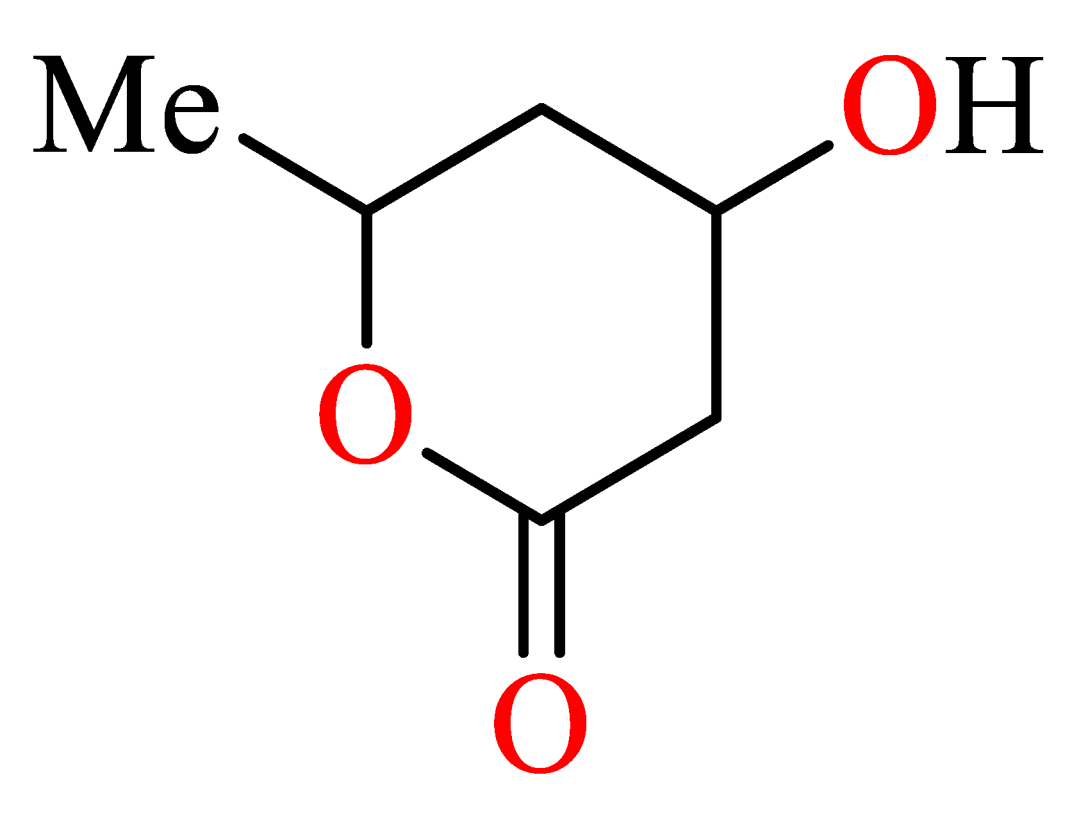	H	F	78	100	250	500	250	500	250
**59f**		H	Me	72	200	500	100	250	250	200
**59g**		Me	F	75	250	250	250	200	250	250
**59h**		Me	Me	70	250	250	250	125	100	125
**59i**	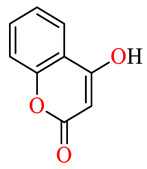	H	F	73	250	100	500	100	200	100
**59j**		H	Me	83	200	250	500	125	100	250
**59k**		Me	F	81	100	100	62.5	200	125	125
**59l**		Me	Me	84	500	200	200	100	200	100
**59m**	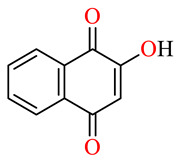	H	F	75	200	100	500	200	250	200
**59n**		H	Me	86	500	500	200	200	200	500
**59o**		Me	F	70	125	500	500	125	100	100
**59p**		Me	Me	82	100	100	500	200	250	100
**59q**	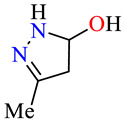	H	F	68	250	500	250	100	125	250
**59r**		H	Me	73	500	250	250	250	250	500
**59s**		Me	F	75	250	250	250	62.5	100	250
**59t**		Me	Me	76	250	250	500	250	500	250
Ampicillin ^a^	--	--	--	--	100	250	100	100	100	250
Norfloxacin ^a^	--	--	--	--	10	50	10	10	10	100
Chloramphenicol ^a^	--	--	--	--	50	50	50	50	50	50
Ciprofloxacin ^a^	--	--	--	--	50	100	25	25	25	50

*BS (Bacillus subtilis)*, *CT (Clostridium tetani)*, *SA (Staphylococcus aureus)*, *EC (Escherichia coli)*, *ST (Salmonella typhi)*, *VC (Vibrio cholerae)*. ^a^ Positive control for the study.

**Table 17:** Three-component synthesis of pyrazole-thiobarbituric derivatives **61** and their in vitro antibacterial activity against three Gram-positive bacterial strains.

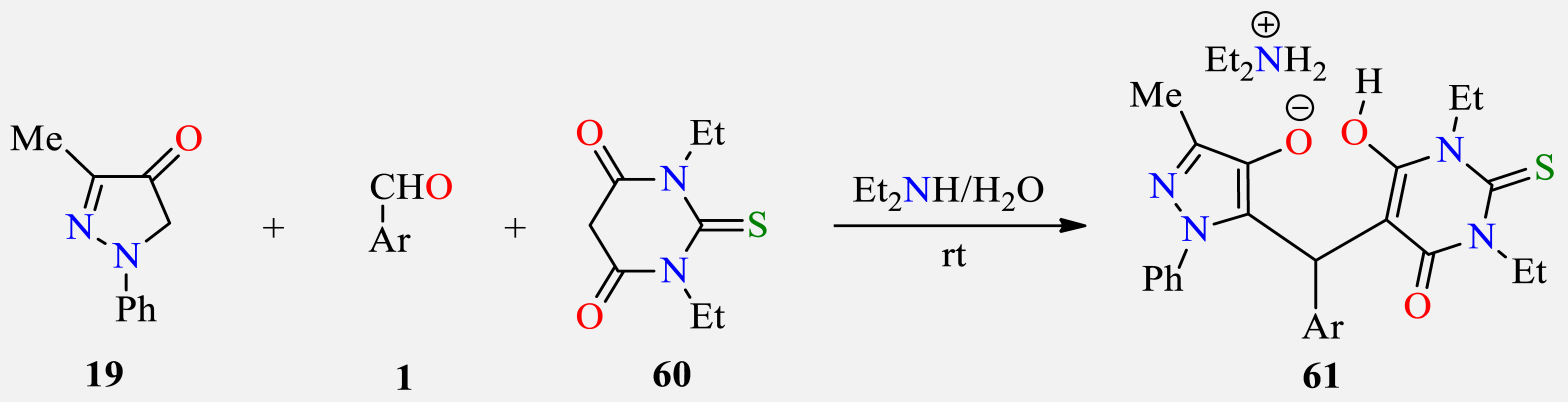								
Compound	Ar	Yield of 61 (%) ^a^	Antibacterial Activity of Compounds 61 (MBC in μg/L) ^a^					
			*SA*		*EF*		*BS*	
			*CPM* (mm)	*MIC* (μg/L)	*CPM* (mm)	*MIC* (μg/L)	*CPM* (mm)	*MIC* (μg/L)
			13	32	15	32	12	32
**61b**	Ph	83	12	32	12	32	10	32
**61c**	4-ClC_6_H_4_	84	14	16	12	16	11	32
**61d**	4-MeC_6_H_4_	73	Nil	128	9	128	11	64
**61e**	3-MeC_6_H_4_	78	Nil	128	10	64	11	64
**61f**	4-BrC_6_H_4_	88	Nil	128	10	64	11	64
**61g**	3-BrC_6_H_4_	73	13	32	12	64	11	64
**61h**	4-NO_2_C_6_H_4_	73	13	32	16	32	11	64
**61i**	3-NO_2_C_6_H_4_	72	14	32	16	32	13	32
**61j**	4-MeOC_6_H_4_	69	14	32	18	16	13	32
**61k**	4-CF_3_C_6_H_4_	63	13	64	10	32	11	32
**61l**	2,4-Cl_2_C_6_H_3_	68	13	32	20	32	15	16
**61m**	2,6-Cl_2_C_6_H_3_	65	13	32	24	32	16	32
**61n**	2-Naphthyl	67	14	32	11	32	11	32
**61o**	Thiophen-2-yl	78	14	32	Nil	32	11	16
Ciprofloxacin ^b^	--	--	27	≤0.25	24	≤0.25	25	≤0.25

^a^*SA (Staphylococcus aureus)*, *EF (Staphylococcus faecalis)*, *BS (Bacillus subtilis)*, *ST (Salmonella typhimurium)*, *KP (Klebsiella pneumonia)*. ^b^ Standard drug.

**Table 18:** Three-component synthesis of pyrazolo-diazepinones (**64–70**)**a**/(**71–77**)**b** and their in vitro antibacterial activity (MIC) against six bacterial strains.

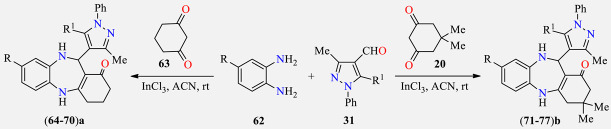									
Compound	R	R^1^	Yield of (64–70)a/(71–77)b (%)	Antibacterial Activity of Compounds (64–70)a/(71–77)b (MIC in μg/mL)					
				Gram-Positive Bacteria			Gram-Negative Bacteria		
				*SP*	*VC*	*EC*	*EC*	*EC*	*EC*
**64a**	H	PhO	87	250	500	250	500	500	500
**64′a**	COPh	PhO	76	100	250	500	500	250	500
**65a**	H	MeC_6_H_4_O	67	250	250	500	250	500	250
**65′a**	COPh	MeC_6_H_4_O	78	200	250	250	500	250	200
**66a**	H	ClC_6_H_4_O	79	250	250	500	200	500	500
**66′a**	COPh	ClC_6_H_4_O	89	125	125	500	250	250	250
**67a**	H	PhS	87	200	200	500	500	500	500
**67′a**	COPh	PhS	74	250	250	250	250	500	500
**68a**	H	ClC_6_H_4_S	87	125	250	250	500	250	250
**68′a**	COPh	ClC_6_H_4_S	73	200	200	200	250	250	200
**69a**	H	Allyl-S	86	125	250	500	250	500	500
**69′a**	COPh	Allyl-S	64	250	250	500	100	500	500
**70a**	H	Bn-S	64	200	250	500	200	500	500
**70′a**	COPh	Bn-S	69	500	200	500	250	500	500
**71b**	H	PhO	79	200	200	500	200	250	250
**71′b**	COPh	PhO	69	62.5	125	500	250	250	500
**72b**	H	MeC_6_H_4_O	77	200	200	250	50	250	200
**72′b**	COPh	MeC_6_H_4_O	72	125	125	200	200	100	125
**73b**	H	ClC_6_H_4_O	87	125	200	125	125	125	200
**73′b**	COPh	ClC_6_H_4_O	75	62.5	62.5	100	62.5	100	62.5
**74b**	H	PhS	89	200	250	500	250	250	200
**74′b**	COPh	PhS	86	200	200	250	200	200	200
**75b**	H	ClC_6_H_4_S	79	125	200	250	200	250	200
**75′b**	COPh	ClC_6_H_4_S	68	62.5	125	62.5	125	50	100
**76b**	H	Allyl-S	58	250	250	500	250	250	500
**76′b**	COPh	Allyl-S	76	200	200	500	250	200	250
**77b**	H	Bn-S	70	250	250	500	500	250	500
**77′b**	COPh	Bn-S	72	250	500	500	500	500	500
Gentamycin ^a^	--	--	--	0.5	5	1	5	5	0.05
Ampicillin ^a^	--	--	--	100	250	250	100	100	100
Chloramphenicol ^a^	--	--	--	50	50	50	50	50	50
Ciprofloxacin ^a^	--	--	--	50	100	50	25	25	25
Norfloxacin ^a^	--	--	--	10	50	100	10	10	10

*SP (Streptococcus pneumoniae)*, *CT (Clostridium tetani)*, *BS (Bacillus subtilis)*, *ST (Salmonella typhi)*, *VC (Vibrio cholera)*, *EC (Escherichia coli)*. ^a^ Standard drugs.

**Table 19:** Three-component synthesis of thiazolo[2,3-*b*]dihydropyrimidinones **80** and their in vitro antibacterial activity against four bacterial strains.

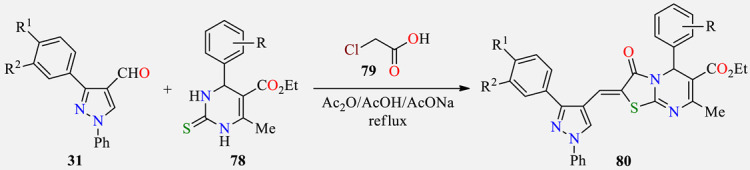								
Compound	R	R^1^	R^2^	Yield of 80 (%)	Antibacterial Activity of Compounds 80 (Zone of Inhibition in mm)			
					*EC*	*SA*	*PA*	*KP*
**80a**	3-F, 4-Me	Cl	Cl	91	17.9	15.9	15.9	15.4
**80b**	2,5-(OMe)_2_	Cl	Cl	89	17.5	15.2	14.4	16.2
**80c**	H	Cl	Cl	67	11.2	11.9	10.6	12.9
**80d**	4-OMe	Cl	Cl	88	16.3	13.7	15.2	14.4
**80e**	3-F, 4-Me	F	F	72	12.2	12.4	11.1	10.4
**80f**	2,5-(OMe)_2_	F	F	74	9.9	9.5	11.7	10.3
**80g**	H	F	F	81	7.1	5.3	10.6	8.1
**80h**	4-OMe	F	F	73	9.3	9.1	7.2	9.4
**80i**	3-F, 4-Me	Cl	H	59	9.5	11.2	12.2	10.6
**80j**	2,5-(OMe)_2_	Cl	H	72	11.6	8.2	11.6	12.4
**80k**	H	Cl	H	70	7.7	5.2	10.4	5.1
**80l**	4-OMe	Cl	H	49	10.9	11.5	10.1	7.0
**80m**	3-F, 4-Me	F	H	66	8.1	9.9	5.4	9.6
**80n**	2,5-(OMe)_2_	F	H	75	10.1	11.4	11.6	12.8
**80o**	H	F	H	86	7.2	9.6	11.4	9.1
**80p**	4-OMe	F	H	76	7.4	10.0	11.2	11.1
Streptomycin ^a^	--	--	--	--	18.5	16.2	19.2	16.6

*EC (Escherichia coli)*, *SA (Staphylococcus aureus)*, *PA (Pseudomonas aeruginosa)*, *KP (Klebsiella pneumoniae)*. ^a^ Standard drug for the study.

**Table 20:** Four-component synthesis of 1*H*-1,2,3-triazole-tethered pyrazolo[3,4-*b*]pyridin-6(7*H*)-ones **84** and their in vitro antibacterial activity against four bacterial strains.

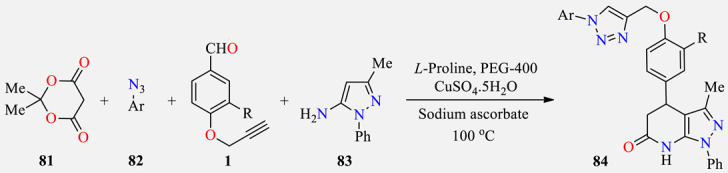							
Compound	Ar	R	Yield of 84 (%)	Antibacterial Activity of Compounds 84 (Diameter of Growth of Inhibition Zone (mm))			
				*SA*	*BS*	*SE*	*BC*
**84a**	4-MeOC_6_H_4_	H	82	16.0	17.3	19.6	18.6
**84b**	3-Cl,4-FC_6_H_3_	H	90	15.3	16.3	17.3	14.3
**84c**	4-FC_6_H_4_	H	86	15.3	15.6	16.3	16.3
**84d**	4-BrC_6_H_4_	H	84	14.3	16.3	15.3	17.0
**84e**	4-MeC_6_H_4_	H	80	18.6	20.3	19.3	21.6
**84f**	3-ClC_6_H_4_	H	84	16.0	17.3	20.6	19.3
**84g**	3-Cl,4-FC_6_H_3_	MeO	92	17.3	19.3	20.6	18.6
**84h**	4-FC_6_H_4_	MeO	84	15.3	15.6	17.3	16.6
**84i**	4-BrC_6_H_4_	MeO	86	14.6	16.3	15.3	16.3
**84j**	4-MeOC_6_H_4_	MeO	80	19.3	21.6	20.3	21.3
**84k**	4-MeC_6_H_4_	MeO	82	21.6	22.3	22.6	23.6
**84l**	3-ClC_6_H_4_	MeO	84	18.6	20.3	18.6	19.3
Ciprofloxacin ^a^	--	--	--	26.6	24.0	19.6	23.0

*SA (Staphylococcus aureus)*, *BS (Bacillus subtilis)*, *SE (Staphylococcus epidermidis)*, *BC (Bacillus cereus)*. ^a^ Standard drug for the study.

**Table 21:** Four-component synthesis of pyrano[2,3-*c*]pyrazoles **86** and their in vitro antibacterial activity against three bacterial strains.

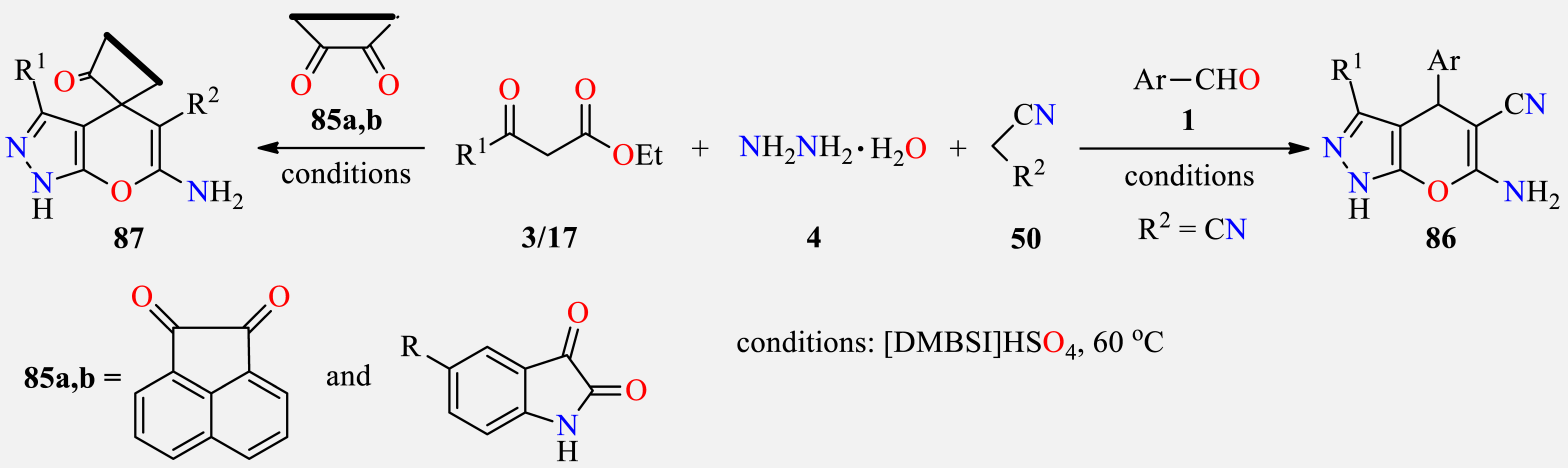						
Compound	Ar	R^1^	Yield of 86 (%)	Antibacterial Activity of Compounds 86 (Diameter of Growth of Inhibition Zone (mm))		
				*ML*	*BS*	*PA*
**86a**	4-ClC_6_H_4_	Me	95	--	11	--
**86b**	4-MeC_6_H_4_	Me	90	--	--	--
**86c**	4-MeOC_6_H_4_	Me	88	--	--	--
**86d**	3-ClC_6_H_4_	Me	95	--	--	--
**86e**	Pyridine-3-yl	Me	95	--	--	--
**86f**	3-MeOC_6_H_4_	Me	90	--	--	--
**86g**	Naphtalen-1-yl	Me	85	--	--	--
**86h**	Thiophen-2-yl	Me	95	--	--	--
**86i**	9*H*-Fluoren-2-yl	Me	86	10	8	--
**86j**	2,4-Cl_2_C_6_H_3_	Me	96	--	--	--
**86k**	3,4-(MeO)_2_C_6_H_3_	Me	87	14	--	--
**86l**	Naphtalen-2-yl	Me	86	16	11	--
**86m**	4-ClC_6_H_4_	Ph	90	--	--	--
**86n**	3-NO_2_C_6_H_4_	Ph	95	8	8	--
**86o**	3-BrC_6_H_4_	Ph	94	--	--	--
Erythromycin ^a^	--	--	--	10	12	10
Tetracycline ^a^	--	--	--	16	14	18

*ML (Micrococcus Luteus)*, *BS (Bacillus subtilis)*, *PA (Pseudomonas aeruginosa)*. ^a^ Standard drug for the study.

**Table 22:** Four-component synthesis of spiro-conjugated pyrano[2,3-*c*]pyrazoles **87** and their in vitro antibacterial activity against three bacterial strains.

Compound	R^2^	R^1^	R	Yield of 87 (%)	Antibacterial Activity of Compounds 87 (Diameter of Growth of Inhibition Zone (mm))		
					*ML*	*BS*	*PA*
**87a**	CN	Me	--	96	--	--	--
**87b**	CO_2_Et	Me	--	93	8	8	--
**87c**	CN	Pr	--	96	8	5	--
**87d**	CN	Me	H	96	--	--	10
**87e**	CN	Pr	H	95	8	--	--
**87f**	CN	Ph	H	88	--	--	--
**87g**	CN	Me	Cl	85	14	--	--
Erythromycin ^a^	--	--	--	--	10	12	10
Tetracycline ^a^	--	--	--	--	16	14	18

*ML (Micrococcus Luteus)*, *BS (Bacillus subtilis)*, *PA (Pseudomonas aeruginosa)*. ^a^ Standard drug for the study.

**Table 23:** Five-component synthesis of dihydropyrano[2,3-*c*]pyrazoles **88** and their in vitro antibacterial activity against four bacterial strains.

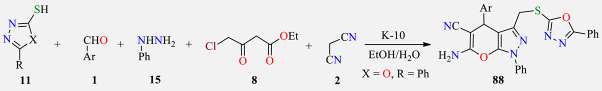						
Compound	Ar	Yield of 88 (%)	Antibacterial Activity of Compounds 88 (Zone of Inhibition (in mm) at conc. 100 mg/mL after 24 h)			
			Gram-Positive Bacteria		Gram-Negative Bacteria	
			*BS*	*SA*	*EC*	*PA*
**88a**	4-MeOC_6_H_4_	81	--	--	--	--
**88b**	4-MeC_6_H_4_	85	3.8	3.0	2.9	2.6
**88c**	4-HOC_6_H_4_	84	9.1	8.8	8.7	8.0
**88d**	Ph	82	4.9	4.6	4.5	4.3
**88e**	4-FC_6_H_4_	80	18.8	17.1	17.5	15.2
**88f**	3-FC_6_H_4_	83	13.6	12.2	10.9	9.6
**88g**	3-HOC_6_H_4_	84	5.6	5.1	5.6	5.6
**88h**	2-HOC_6_H_4_	86	7.5	7.4	6.3	7.0
**88i**	2-MeC_6_H_4_	83	--	2.8	--	--
**88j**	2-MeOC_6_H_4_	89	--	--	--	--
**88k**	2-ClC_6_H_4_	88	11.4	10.4	10.2	9.1
**88l**	4-ClC_6_H_4_	82	15.6	13.1	12.7	11.0
**88m**	4-BrC_6_H_4_	84	9.8	9.3	8.9	8.2
**88n**	4-NO_2_C_6_H_4_	83	16.2	15.2	15.3	13.7
Tetracycline ^a^	--	--	19.4	18.1	16.8	14.2

*BS (Bacillus subtilis)*, *SA (Staphylococcus aureus)*, *EC (Escherichia coli)*, *PA (Pseudomonas aeruginosa)*. ^a^ Standard drug for this study.

**Table 24:** Four-component synthesis of dihydropyrano[2,3-*c*]pyrazole **90** and their in vitro antibacterial activity against four bacterial strains.

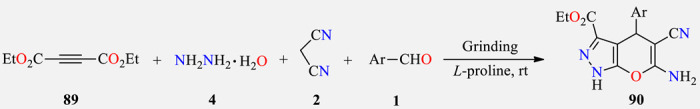						
Compound	Ar	Yield of 90 (%)	Antibacterial Activity of Compounds 90 (Inhibition Zone (mm))			
			*SAl*	*SP*	*KP*	*PA*
**90a**	Ph	79	17	7	9	12
**90b**	4-MeC_6_H_4_	80	12	R	R	10
**90c**	2-ClC_6_H_4_	69	12	R	R	10
**90d**	4-ClC_6_H_4_	88	R	R	R	R
**90e**	4-FC_6_H_4_	93	R	4	10	R
**90f**	2-furyl	65	R	R	13	3
**90g**	2-thienyl	69	7	9	5	4
**90h**	4-EtC_6_H_4_	82	15	15	7	7
**90i**	4-HOC_6_H_4_	88	22	13	15	12
**90j**	2-MeOC_6_H_4_	72	15	11	17	7
**90k**	4-HO, 3-MeOC_6_H_3_	78	10	8	12	9
**90l**	4-MeOC_6_H_4_	81	8	10	9	7
**90m**	4-NO_2_C_6_H_4_	92	11	9	13	8
Control ^a^	--	--	R	R	R	R
Amikacin ^b^	--	--	26.6	24.0	19.6	23.0

*SAl (Staphylococcus albus)*, *SP (Streptococcus pyogenes)*, *KP (Klebsiella pneumonia)*, *PA (Pseudomonas aeruginosa)*. ^a^ DMSO. ^b^ Standard drug for the study. Not active (R, inhibition zone < 2 mm); weak activity (2−8 mm); moderate activity (9−15 mm); strong activity (> 15 mm).

**Table 25:** SBA-Pr-SO_3_H-Catalyzed three-component synthesis of pyrazolopyranopyrimidines **91** and their in vitro antibacterial activity against four bacterial strains.

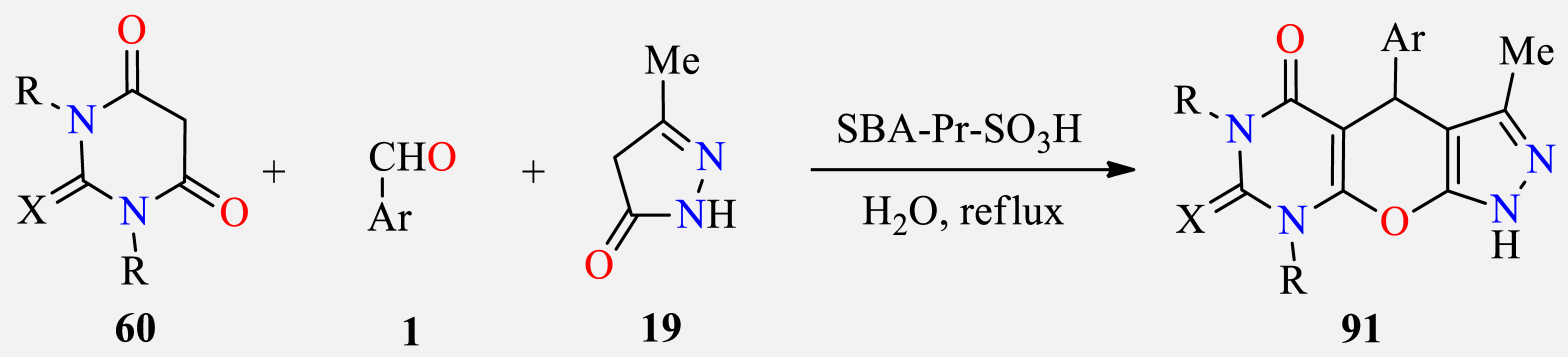								
Compound	Ar	R	X	Yield of 91 (%)	Antibacterial Activity of Compounds 91 (Inhibition Zone (mm))			
					*BS*	*EA*	*EC*	*PA*
**91a**	4-ClC_6_H_4_	H	O	92	20	24	12	0
**91b**	4-MeC_6_H_4_	H	O	93	18	22	12	0
**91c**	Ph	H	O	94	15	21	13	0
**91d**	2-MeOC_6_H_4_	H	O	95	14	19	12	0
**91e**	3-NO_2_C_6_H_4_	H	O	89	16	24	0	0
**91f**	4-ClC_6_H_4_	Me	O	90	21	24	12	0
**91g**	4-MeC_6_H_4_	Me	O	91	19	23	14	0
**91h**	Ph	Me	O	96	21	25	26	0
**91i**	4-MeC_6_H_4_	H	S	91	16	20	12	0
Chloramphenicol ^a^	--	--	--	--	26	22	24	8
Gentamicin ^a^	--	--	--	--	28	20	20	18

*BS (Bacillus subtilis)*, *SA (Staphylococcus aureus)*, *EC (Escherichia coli)*, *PA (Pseudomonas aeruginosa)*. ^a^ Standard drugs for the study.

**Table 26:** Four-component synthesis of coumarin-substituted pyrazoles **93**, **94**, and **95** and their in vitro antibacterial activity (MIC) against four bacterial strains.

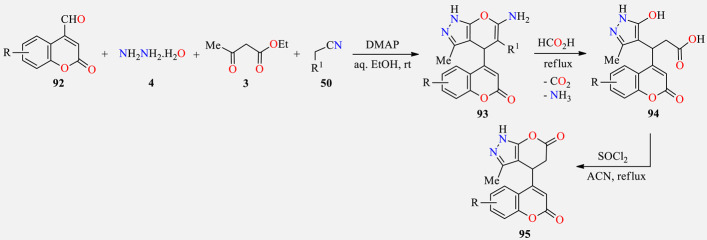							
Compound	R	R^1^	Yield of 93, 94 and 95 (%)	Antibacterial Activity of Compounds 93, 94 and 95 (Minimum Inhibitory Concentration in μg/mL (MIC))			
				*SA*	*SF*	*EC*	*PA*
**93a**	6-Me	CN	94	6.25	12.5	12.5	12.5
**93b**	6-OMe	CN	91	3.125	12.5	6.25	1.56
**93c**	6-Cl	CN	89	3.125	6.25	3.125	12.5
**93d**	7-Me	CN	92	6.25	12.5	0.78	3.125
**93e**	7,8-Benzo	CN	90	6.25	12.5	25.0	25.0
**93f**	6-Me	CO_2_Et	92	3.125	6.25	12.5	6.25
**93g**	6-OMe	CO_2_Et	89	1.56	3.125	6.25	6.25
**93h**	6-Cl	CO_2_Et	86	3.125	12.5	6.25	50.0
**93i**	7-Me	CO_2_Et	91	3.125	25.0	3.125	12.5
**93j**	7,8-Benzo	CO_2_Et	88	3.125	12.5	6.25	6.25
**94a**	6-Me	--	89	3.125	12.5	12.5	25.0
**94b**	6-OMe	--	80	0.78	1.56	3.125	6.25
**94c**	6-Cl	--	77	1.56	6.25	25.0	50.0
**94d**	7-Me	--	82	3.125	6.25	6.25	12.5
**94e**	7,8-Benzo	--	89	6.25	12.5	6.25	12.5
**95a**	6-Me	--	85	6.25	12.5	3.125	12.5
**95b**	6-OMe	--	82	3.125	6.25	6.25	6.25
**95c**	6-Cl	--	81	6.25	12.5	6.25	12.5
**95d**	7-Me	--	84	6.25	6.25	12.5	6.25
**95e**	7,8-Benzo	--	83	12.5	12.5	12.5	6.25
Ciprofloxacin ^a^	--	--	--	6.25	6.25	3.125	6.25

*SA (Staphylococcus aureus)*, *SF (Staphylococcus faecalis)*, *EC (Escherichia coli)*, *PA (Pseudomonas aeruginosa)*. ^a^ Standard drug for the study.

**Table 27:** Cytotoxic activity of coumarin-substituted thiazolyl-3-aryl-pyrazole-4-carbaldehydes **25**.

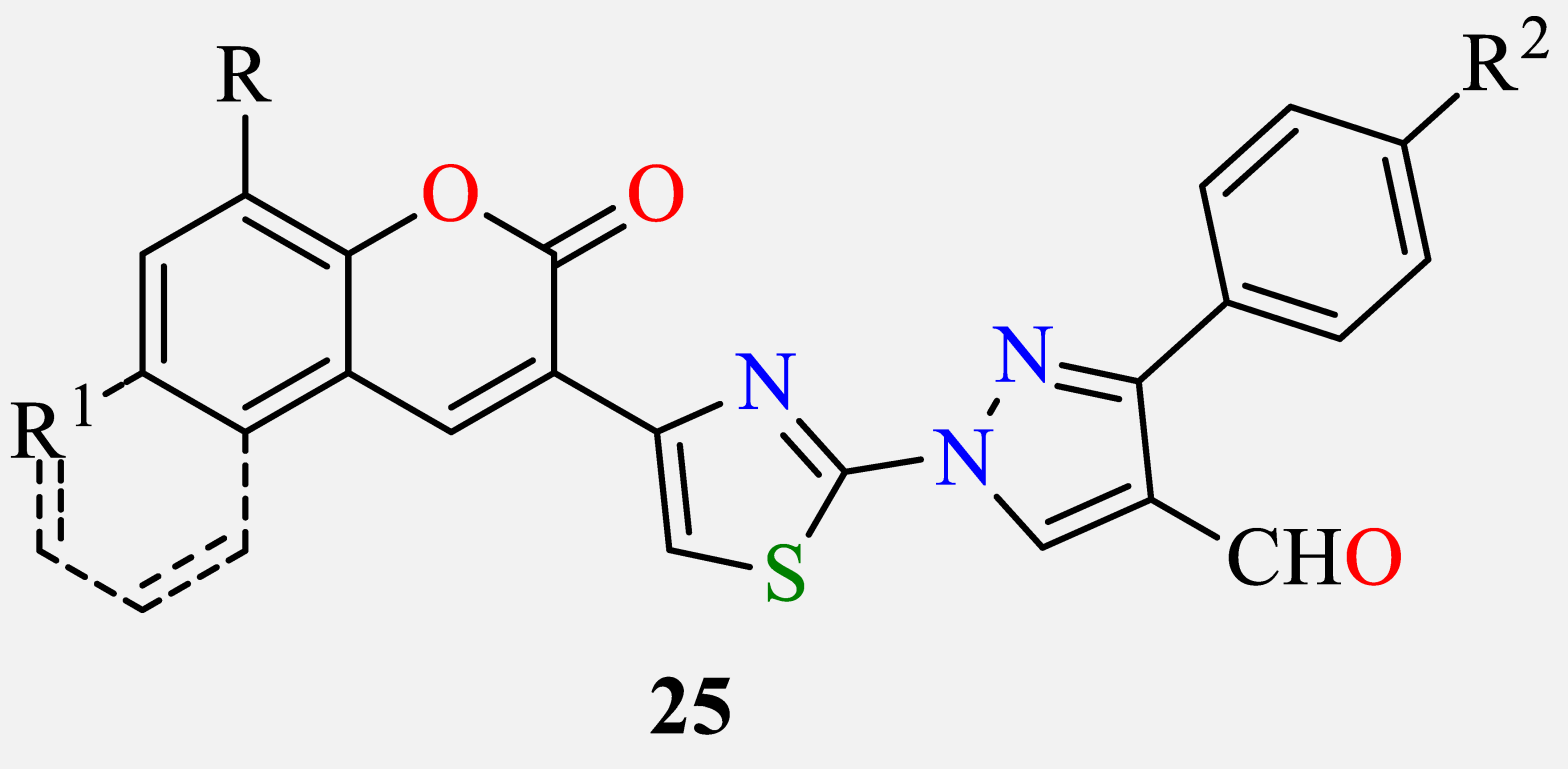						
Compound	R	R^1^	R^2^	IC_50_ (μM)		
				DU-145	MCF-7	HeLa
**25a**	H	H	H	-- ^a^	-- ^a^	-- ^a^
**25b**	H	Cl	H	-- ^a^	-- ^a^	12.82
**25c**	Cl	Cl	H	41.05	76.33	13.75
**25d**	H	Br	H	35.01	18.16	11.02
**25e**	Br	Br	H	27.97	22.23	13.69
**25f**	H	Benzo	H	11.91	39.46	13.11
**25g**	H	Cl	Cl	14.86	18.67	9.51
**25h**	Cl	Cl	Cl	30.90	21.74	10.29
**25i**	H	Br	Cl	22.32	85.03	9.46
**25j**	MeO	H	Cl	38.18	71.68	41.89
**25k**	H	H	Me	50.23	69.45	37.36
**25l**	H	Br	Me	20.86	35.17	14.13
**25m**	Cl	Cl	Me	14.71	14.56	5.75
**25n**	Br	Br	Me	10.81	24.52	6.25
**25o**	MeO	H	Me	31.42	42.57	28.19
Doxorubicin ^b^	--	--	--	2.49	3.18	3.92

^a^ Not determined. ^b^ Standard drug for the study.

**Table 28:** Four-component synthesis and anticancer activity of pyranopyrazole derivatives **96**.

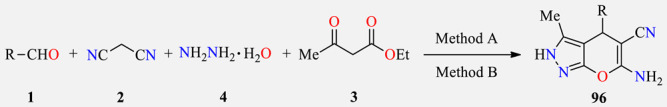				
Compound	R	Yield 96 (%)		IC_50_ (μg/mL)
		Method A	Method B	Hep3B
**96a**	3-HOC_6_H_4_	88	82	32
**96b**	4-BrC_6_H_4_	82	80	16
**96c**	3-BrC_6_H_4_	80	77	10
**96d**	3-NO_2_C_6_H_4_	86	81	32
**96e**	3-Thiophenyl	88	80	24
**96f**	2-Pyrrolyl	85	80	128
**96g**	3-Indolyl	81	79	64
**96h**	4-ClC_6_H_4_	77	70	96
**96i**	2-IC_6_H_4_	80	75	96
**96j**	C_6_H_5_	86	81	128
**96k**	*n*-Butyl	82	83	20
**96l**	4-MeC_6_H_4_	88	87	20
**96m**	4-Pyridinyl	94	85	48
**96n**	2-FC_6_H_4_	90	87	32

Method A: Aldehyde **1** (2.2 mmol), malononitrile **2** (2.2 mmol), hydrazine hydrate **4** (2.0 mmol), ethyl acetoacetate **3** (2.0 mmol), Et_3_N (3.0 mmol), EtOH (4 mL), 1–2 h, r.t. Method B: Aldehyde **1** (2.2 mmol), malononitrile **2** (2.2 mmol), hydrazine hydrate **4** (2.0 mmol), ethyl acetoacetate **3** (2.0 mmol), Et_3_N (3.0 mmol), EtOH (4 mL), 3–5 min, 60 °C, MWI.

**Table 29:** Pseudo five-component synthesis and anticancer activity of *bis*-1,4-dihydropyrano[2,3-*c*]pyrazole-5-carbonitrile derivatives **97**.

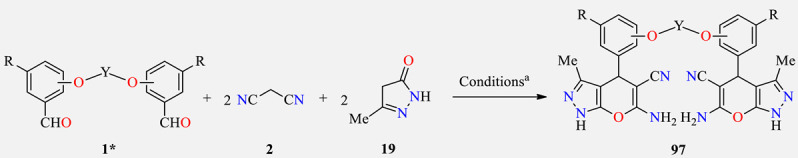						
Compound	R	Y	Yield 97 (%)	IC_50_ (mM)		
				A549	MCF-7	HEPG2
**97a**	H	(CH_2_)_2_	87	0.46	2.30	3.34
**97b**	Br	(CH_2_)_2_	85	0.98	1.99	0.99
**97c**	H	(CH_2_)_3_	82	0.78	0.29	0.42
**97d**	Br	(CH_2_)_3_	79	0.71	1.56	0.46
**97e**	H	(CH_2_)_4_	85	0.37	0.31	0.014
**97f**	Br	(CH_2_)_4_	83	0.64	0.79	0.33

^a^ Reaction conditions: *bis*-aldehyde 1* (1 mmol), malononitrile **2** (2.2 mmol), 3-methyl-1*H*-pyrazol-5(4*H*)-one **19** (2.2 mmol), piperidine (0.2 mL), EtOH (5 mL), reflux, 3 h.

**Table 30:** Pd-Catalyzed one-pot four-component reaction of pyrazolo[1,5-*c*]quinazolines **104** and **105** as potential EGFR inhibitors.

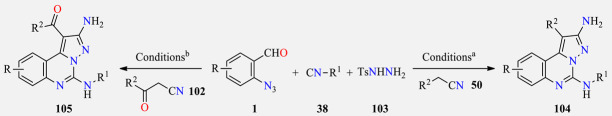							
Compound	R	R^1^	R^2^	Yield 104/105 (%)	IC_50_ (μM)		
					MDA-MB-231	A549	H1299
**104a**	H	*t*Bu	CN	93	6.86	3.98	4.97
**104b**	H	CMe_2_CH_2_CMe_3_	CN	86	1.96	2.87	2.02
**104c**	4-Br	*t*Bu	CN	75	5.89	4.98	6.35
**104d**	4-Br	*t*Bu	Ph	0	--	--	--
**104e**	4-Cl	*t*Bu	CN	82	7.98	5.93	4.86
**104f**	4-Cl	CMe_2_CH_2_CMe_3_	CN	78	5.65	4.56	4.93
**104g**	5-Cl	CMe_2_CH_2_CMe_3_	CN	89	8.54	10.44	9.34
**104h**	5-Cl	CMe_2_CH_2_CMe_3_	CO_2_Me	76	7.83	5.27	5.01
**104i**	H	*t*Bu	CO_2_Me	84	7.90	8.33	6.65
**104j**	H	CMe_2_CH_2_CMe_3_	CO_2_Me	73	4.69	2.78	4.94
**104k**	H	*t*Bu	CO_2_Et	77	8.83	4.89	6.99
**104l**	H	CMe_2_CH_2_CMe_3_	CO_2_Et	79	7.82	5.89	4.75
**104m**	H	*t*Bu	CO_2_*i*Pr	77	4.76	3.29	3.47
**104n**	H	CMe_2_CH_2_CMe_3_	CO_2_*i*Pr	80	9.36	6.35	5.96
**105a**	H	CMe_2_CH_2_CMe_3_	C_6_H_5_	87	5.28	6.84	7.22
**105b**	H	CMe_2_CH_2_CMe_3_	4-FC_6_H_4_	71	1.93	1.06	1.32
**105c**	H	CMe_2_CH_2_CMe_3_	2-ClC_6_H_4_	65	5.97	6.45	7.84
**105d**	H	CMe_2_CH_2_CMe_3_	4-MeOC_6_H_4_	75	2.45	1.74	2.04
**105e**	5-Cl	CMe_2_CH_2_CMe_3_	C_6_H_5_	70	3.49	2.69	3.22
Erlotinib ^c^	--	--	--	--	4.56	2.98	3.33
Gefitinib ^c^	--	--	--	--	6.85	2.65	3.02

^a^ Reaction conditions: 2-azidobenzaldehyde **1** (1 equiv), isocyanide **38** (1.2 equiv), TsNHNH_2_ **103** (1 equiv), Pd(OAc)_2_ (7.5 mol%), 4Ǻ MS (200 mg), PhMe (1.0 mL), 100 °C, 10 min, then active methylene compound **50** (2 equiv), DABCO (3 equiv), 100 °C, 3 h. ^b^ Reaction conditions: 2-azidobenzaldehyde **1** (1 equiv), isocyanide **38** (1.2 equiv), TsNHNH_2_ **103** (1.1 equiv), Pd(OAc)_2_ (7.5 mol%), 4Ǻ MS (200 mg), PhMe (1.0 mL), 100 °C, 10 min, then aroylacetonitrile **102** (2 equiv), DABCO (3 equiv), I_2_ (10 mol%), 100 °C, 3 h. ^c^ Standard drug for the study.

**Table 31:** Four-component synthesis and in vitro anticancer activity of 4,5-dihydropyrano[2,3-*c*]pyrazol-6(2*H*)-one derivatives **108**.

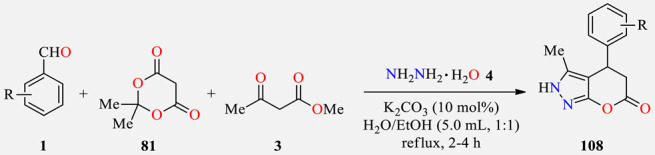					
Compound	R	Yield 108 (%)	IC_50_ (μM)		
			A2780	MCF-7	PC-3
**108a**	3-MeO	66	150	165	90
**108b**	4-MeO	65	150	NA	165
**108c**	2,3-(MeO)_2_	60	150	120	100
**108d**	2-OH-4-MeO	80	NA ^b^	NA	NA
**108e**	2,4-(OH)_2_	85	150	NA	75
**108f**	2,4-(MeO)_2_	68	NA	101	NA
**108g**	2,5-(MeO)_2_	74	104	87	60
**108h**	3,4-(MeO)_2_	75	150	NA	50
**108i**	3,5-(MeO)_2_	70	NA	23	31
**108j**	2,3,4-(OH)_3_	80	NA	NA	NA
**108k**	2,3,4-(MeO)_3_	60	NA	NA	NA
**108l**	3,4,5-(MeO)_3_	59	NA	NA	NA
Doxorubicin ^a^	--	--	3.70	4.76	5.25

^a^ Standard drug for the study. ^b^ NA = not active.

**Table 32:** Evaluation of spiroindenopyridazine-4*H*-pyrans **45** against the cancer cell lines A549, PC-3, MCF-7, A375, LNCaP, and Normal cell HDF ^a^.

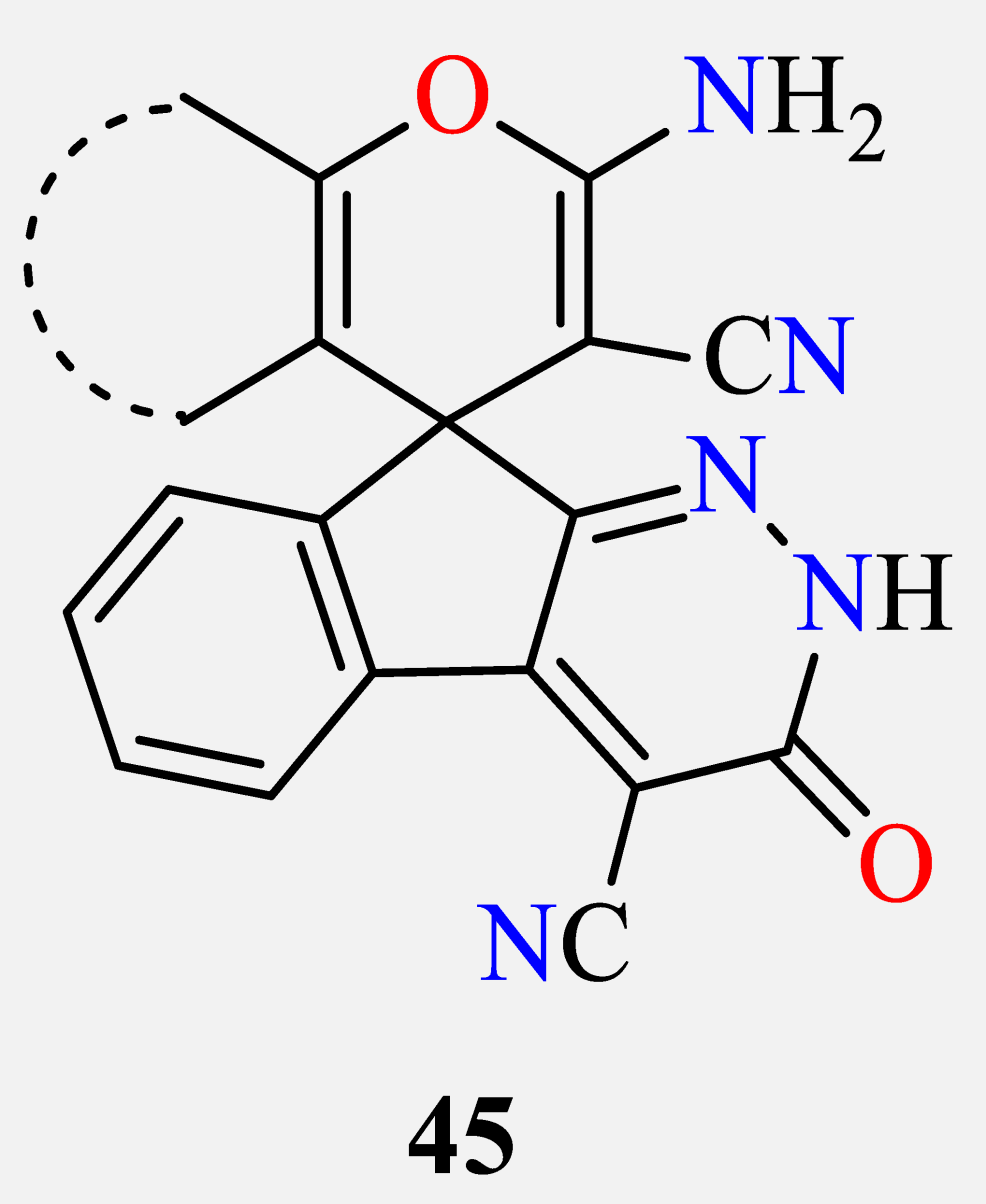							
Compound	Cyclic CH-Acid 44 in Table 10	IC_50_ (μM)					
		A549	PC-3	MCF-7	A375	LNCaP	HDF
**45a**	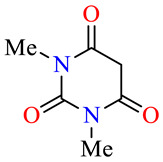	40	>100	>100	70.7	32.1	>100
**45b**	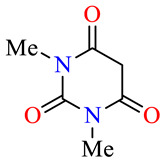	>100	>100	>100	>100	>100	>100
**45c**	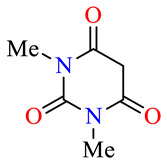	>100	>100	>100	>100	>100	>100
**45d**	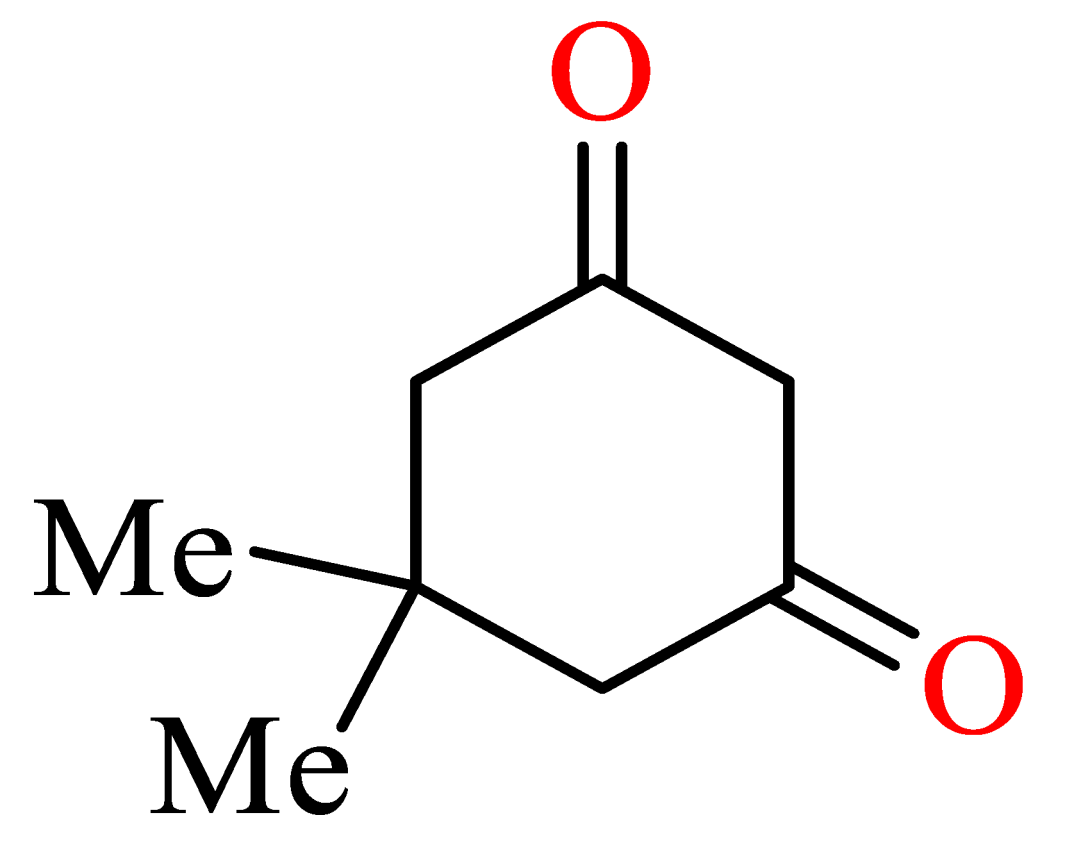	>100	>100	>100	>100	>100	>100
**45e**	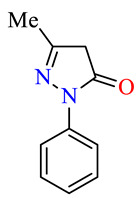	>100	>100	>100	>100	>100	>100
**45f**	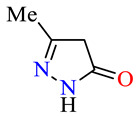	>100	>100	>100	>100	>100	>100
**45g**	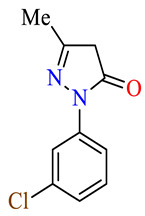	>100	>100	>100	>100	>100	>100
**45h**	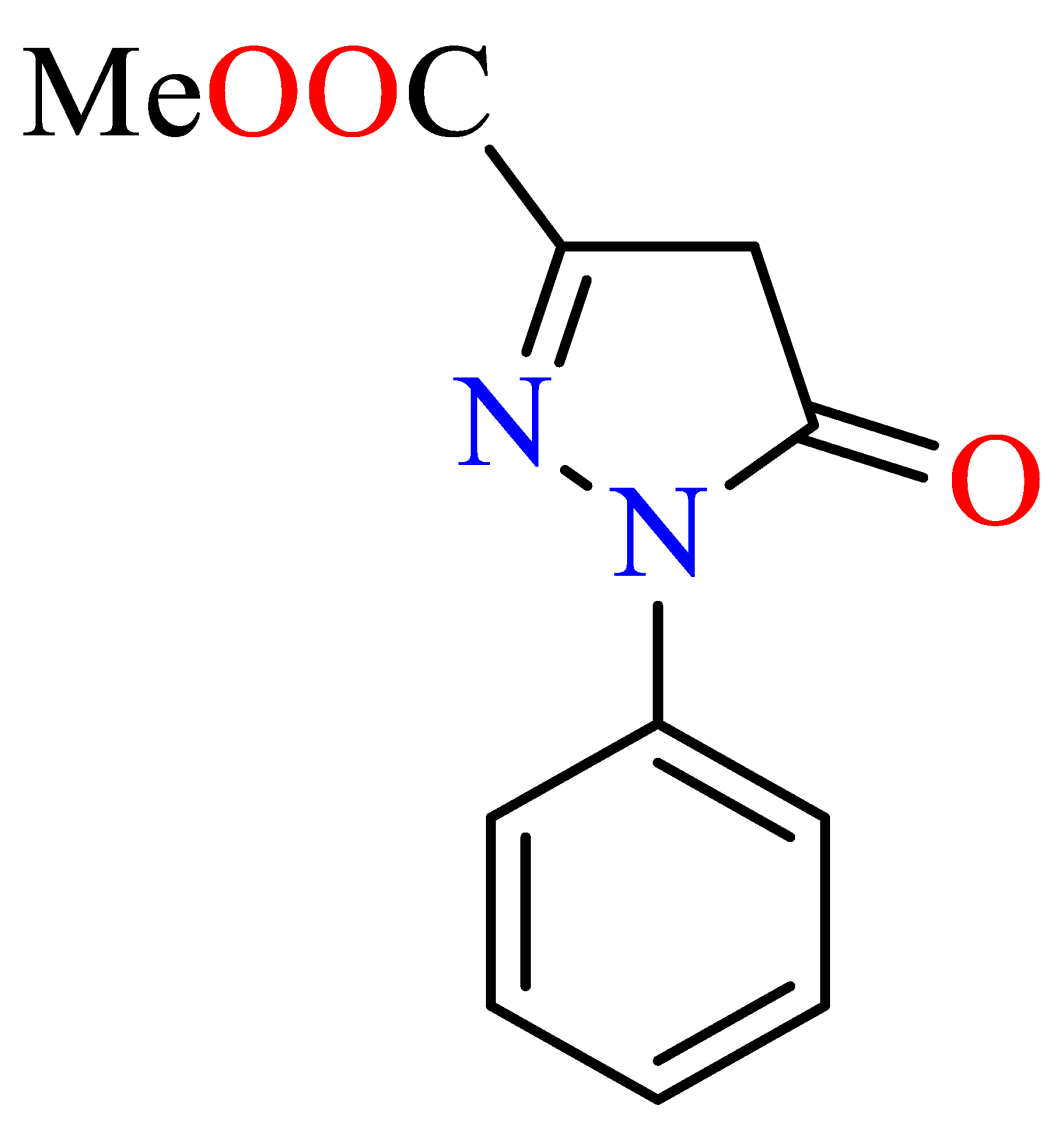	>100	>100	>100	>100	>100	>100
**45i**	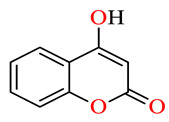	>100	>100	>100	>100	>100	>100
**45j**	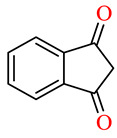	>100	>100	>100	>100	>100	>100
Etoposide ^b^	--	60	40	30	25.3	90	>100

^a^ Reaction conditions: Cyanoacetohydrazide **42** (1 mmol), ninhydrin **43** (1 mmol), malononitrile **2** (1 mmol), cyclic CH-acid **44** (1 mmol), EtOH (10 mL), reflux, 6–12 h. ^b^ Standard drug for the study.

**Table 33:** Four-component synthesis and anticancer evaluation of pyrazole-based 1,4-naphthoquinones **123**.

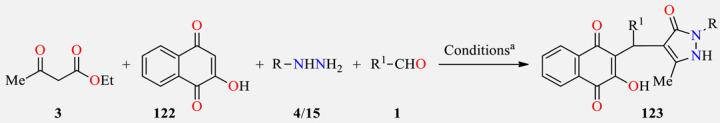				
Compound	R	R^1^	Yield 123 (%)	IC_50_ (μM)
				HeLa
**123a**	C_6_H_5_	4-MeOC_6_H_4_	95	-- ^c^
**123b**	C_6_H_5_	3-OHC_6_H_4_	96	4.81
**123c**	C_6_H_5_	C_6_H_5_	93	22.08
**123d**	C_6_H_5_	3-Me-4-ClC_6_H_3_	92	25.12
**123e**	C_6_H_5_	2-OHC_6_H_4_	91	-- ^c^
**123f**	C_6_H_5_	3-MeO-4-BzOC_6_H_3_	93	4.36
**123g**	C_6_H_5_	2-OH-3-MeOC_6_H_3_	89	-- ^c^
**123h**	C_6_H_5_	2-BrC_6_H_4_	86	9.18
**123i**	H	2-OH-4-MeOC_6_H_3_	88	2.9
**123j**	2-EtC_6_H_4_	2-OH-3-MeOC_6_H_3_	94	-- ^c^
**123k**	4-MeOC_6_H_4_	2-OH-3-MeOC_6_H_3_	92	-- ^c^
**123l**	2-BrC_6_H_4_	2-OH-3-MeOC_6_H_3_	98	-- ^c^
Doxorubicin ^b^	--	--	--	5.1

^a^ Reaction conditions: Ethyl acetoacetate **3** (1 mmol), 2-hydroxy-1,4-naphthaquinone **122** (1 mmol), hydrazine derivative **4**/**15** (1 mmol), aldehyde **1** (1 mmol), V_2_O_5_ (5 mol%), EtOH (5 mL), 80 °C, 1 h. ^b^ Standard drug for the study. ^c^ Not determined.

**Table 34:** Zn(OTf)_2_-catalyzed one-pot four-component synthesis of 6-amino-1,4-dihydropyrano[2,3-*c*]pyrazole-5-carbonitriles **127** as anticancer agents.

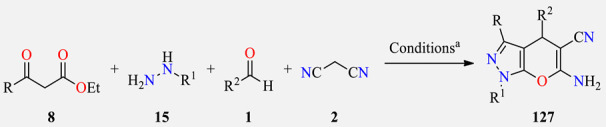								
Compound	R	R^1^	R^2^	Yield 127 (%)	IC_50_ (μg/mL)			
					786-0	A431	MCF-7	U-251
**127a**	CF_3_	C_6_H_5_	C_6_H_5_	93	19.81	55.87	89.76	30.89
**127b**	CF_3_	C_6_H_5_	4-BrC_6_H_4_	98	21.98	22.78	47.88	33.98
**127c**	CF_3_	4-ClC_6_H_4_	C_6_H_5_	95	66.41	55.98	54.87	55.78
**127d**	CF_3_	4-ClC_6_H_4_	4-MeOC_6_H_4_	92	31.86	41.34	44.65	97.98
**127e**	CH_3_	3-NO_2_C_6_H_4_	C_6_H_5_	92	55.78	>100	66.54	55.98
**127f**	CH_3_	3-NO_2_C_6_H_4_	3-Br-4-MeO-C_6_H_3_	99	78.98	>100	55.78	97.98
**127g**	CH_3_	3-NO_2_C_6_H_4_	4-NO_2_C_6_H_4_	97	17.43	>100	>100	>100
**127h**	CH_3_	3-NO_2_C_6_H_4_	4-MeOC_6_H_4_	98	9.9	66.54	31.87	73.67
**127i**	CH_3_	3-NO_2_C_6_H_4_	2-OHC_6_H_4_	94	21.98	21.98	47.89	33.98
**127j**	CH_3_	3-NO_2_C_6_H_4_	5-Br-2-OH-C_6_H_3_	96	45.89	19.98	39.87	25.78
Doxorubicin ^b^	--	--	--	--	0.99	1.6	2.1	1.9

^a^ Reaction conditions: *β*-keto esters **8** (1 mmol), aryl hydrazines **15** (1.1 mmol), and zinc triflate (10 mol%), MWI, 80 °C, 10 min, then aromatic aldehydes **1** (1 mmol) and malononitrile **2** (1.1 mmol), MWI, 120 °C, 15 min. ^b^ Standard drug for the study.

**Table 35:** Anticancer activity of compounds **128** and **129** against PC-3, SKOV3, and HeLa cell lines.

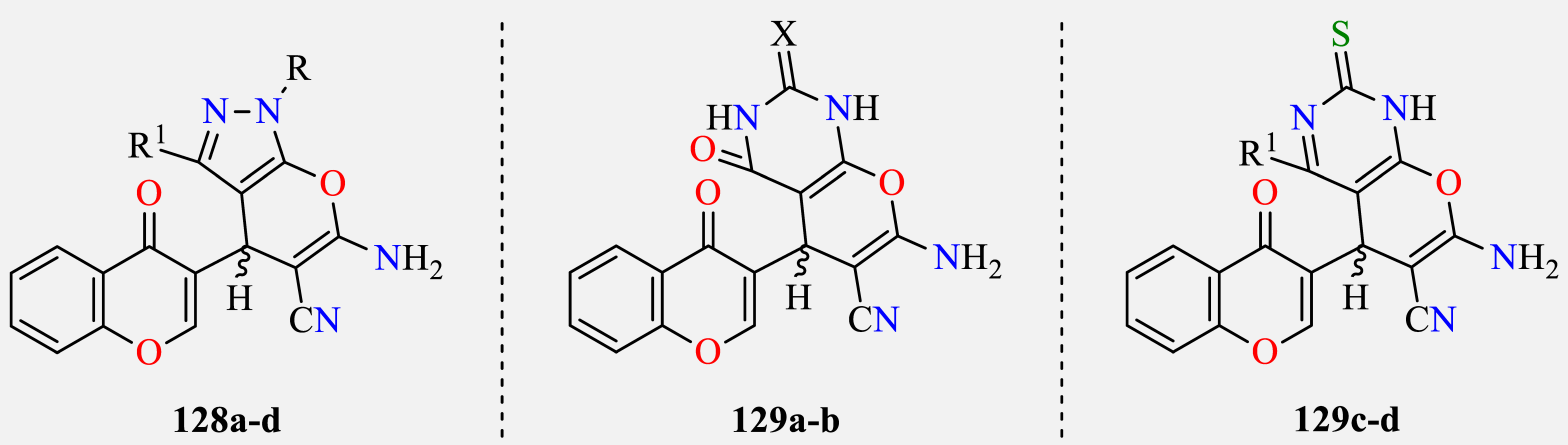							
Compound	R	R^1^	X	Yield 128/129 (%)	IC_50_ (µg/mL)		
					PC-3	SKOV3	HeLa
**128a**	H	Me	--	92	9.7	35.6	25.9
**128b**	H	C_6_H_5_	--	93	34.2	55.4	32.5
**128c**	Me	C_6_H_5_	--	91	13.2	16.5	8.4
**128d**	C_6_H_5_	C_6_H_5_	--	93	190.3	234.3	91.3
**129a**	--	--	O	89	8.9	4.7	11.3
**129b**	--	--	S	90	73.4	9.3	14.3
**129c**	--	Me	--	91	42.1	35.5	32.8
**129d**	--	C_6_H_5_	--	90	18.9	5.4	15.9
Doxorubicin ^a^	--	--	--	--	2.1	2.3	1.9

^a^ Standard drug for the study.

**Table 36:** Antifungal evaluation of pyrazole-thiobarbituric acid derivatives **61**.

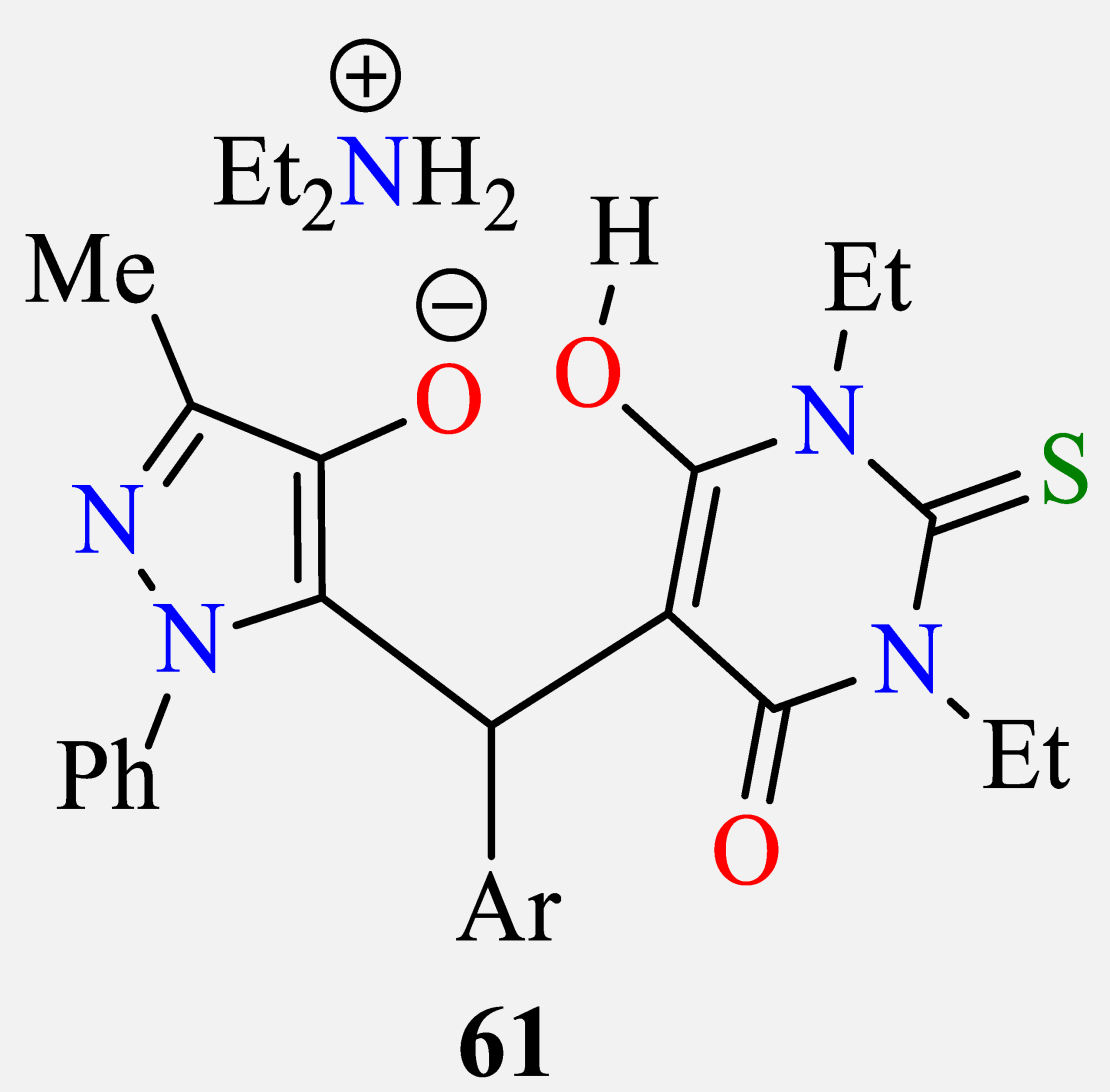			
Compound	Ar	*Candida albicans*	
		CPM (mm)	MIC (μg/L)
**61a**	4-FC_6_H_4_	18	8
**61b**	C_6_H_5_	16	16
**61c**	4-ClC_6_H_4_	16	16
**61d**	4-MeC_6_H_4_	11	64
**61e**	3-MeC_6_H_4_	12	64
**61f**	4-BrC_6_H_4_	15	32
**61g**	3-BrC_6_H_4_	14	32
**61h**	4-NO_2_C_6_H_4_	20	4
**61i**	3-NO_2_C_6_H_4_	14	32
**61j**	4-MeOC_6_H_4_	17	16
**61k**	4-CF_3_C_6_H_4_	16	16
**61l**	2,4-diClC_6_H_3_	21	4
**61m**	2,6-diClC_6_H_3_	15	16
**61n**	2-Naphthyl	16	16
**61o**	2-Thiophenyl	17	8
Fluconazole ^a^	--	28	0.5

^a^ Standard drug for the study.

**Table 37:** Antifungal evaluation of pyrazole-dimedone derivatives **21**.

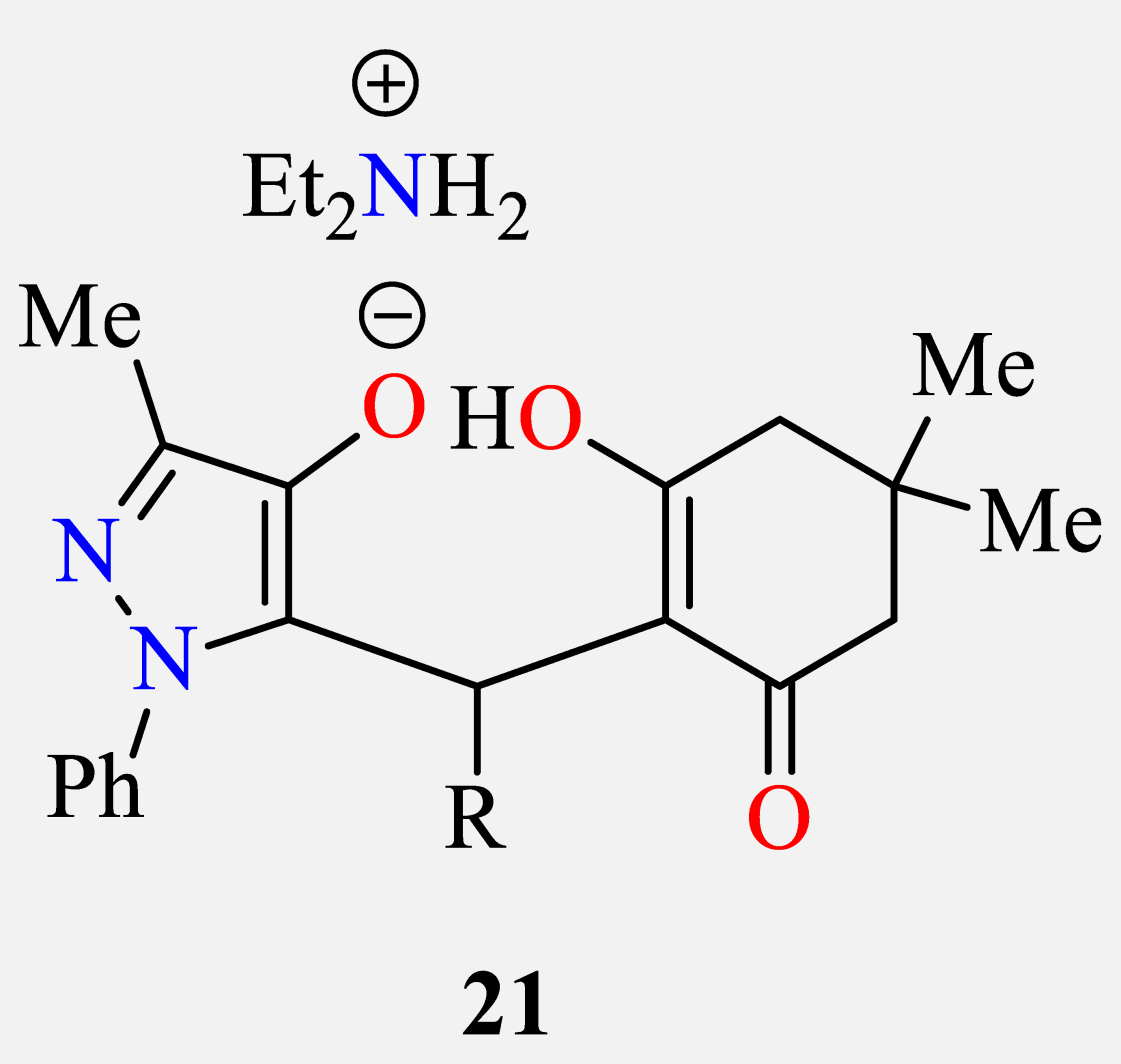			
Compound	R	*Candida albicans*	
		CPM (mm)	MIC (μg/L)
**21a**	2,4-diClC_6_H_3_	14	32
**21b**	C_6_H_5_	15	32
**21c**	4-ClC_6_H_4_	15	16
**21d**	4-MeC_6_H_4_	16	16
**21e**	3-MeC_6_H_4_	14	32
**21f**	4-BrC_6_H_4_	14	32
**21g**	3-BrC_6_H_4_	14	32
**21h**	4-NO_2_C_6_H_4_	17	16
**21i**	3-NO_2_C_6_H_4_	14	32
**21j**	4-MeOC_6_H_4_	13	32
**21k**	4-FC_6_H_4_	15	16
**21l**	4-CF_3_C_6_H_4_	14	32
**21m**	2,6-diClC_6_H_3_	16	16
**21n**	2-Naphthyl	14	32
**21o**	2-Thiophenyl	21	4
Fluconazole ^a^	--	28	0.5

^a^ Standard drug for the study.

**Table 38:** Antifungal activity of polyhydroquinoline derivatives **51**.

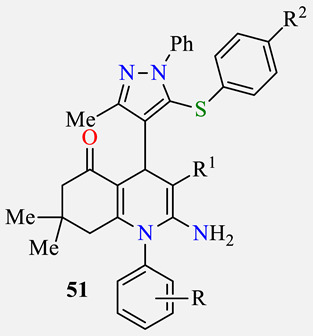					
Compound	R	R^1^	R^2^	*C. albicans*	*A. fumigatus*
				MIC (μg/mL)	MIC (μg/mL)
**51a**	4-F	CN	4-Cl	>1000	>1000
**51b**	4-F	COOEt	4-Cl	1000	>1000
**51c**	4-F	CONH_2_	4-Cl	1000	500
**51d**	4-F	CN	4-Me	500	500
**51e**	4-F	COOEt	4-Me	500	>1000
**51f**	4-CF_3_	CN	4-Me	1000	>1000
**51g**	4-CF_3_	COOEt	4-Me	1000	1000
**51h**	2,4-diF	CN	4-Me	500	>1000
**51i**	2,4-diF	COOEt	4-Me	500	100
**51j**	2,4-diF	CN	4-Cl	250	1000
**51k**	2,4-diF	COOEt	4-Cl	250	1000
**51l**	2,4-diF	CONH_2_	4-Cl	1000	100
**51m**	4-F	CN	4-F	1000	1000
**51n**	4-F	COOEt	4-F	250	1000
**51o**	4-CF_3_	CN	4-F	500	500
**51p**	4-CF_3_	COOEt	4-F	500	250
Griseofulvin ^a^	--	--	--	500	100

^a^ Standard drug for the study.

**Table 39:** Antifungal activity of pyrano[2,3-*c*]pyrazole derivatives **12**.

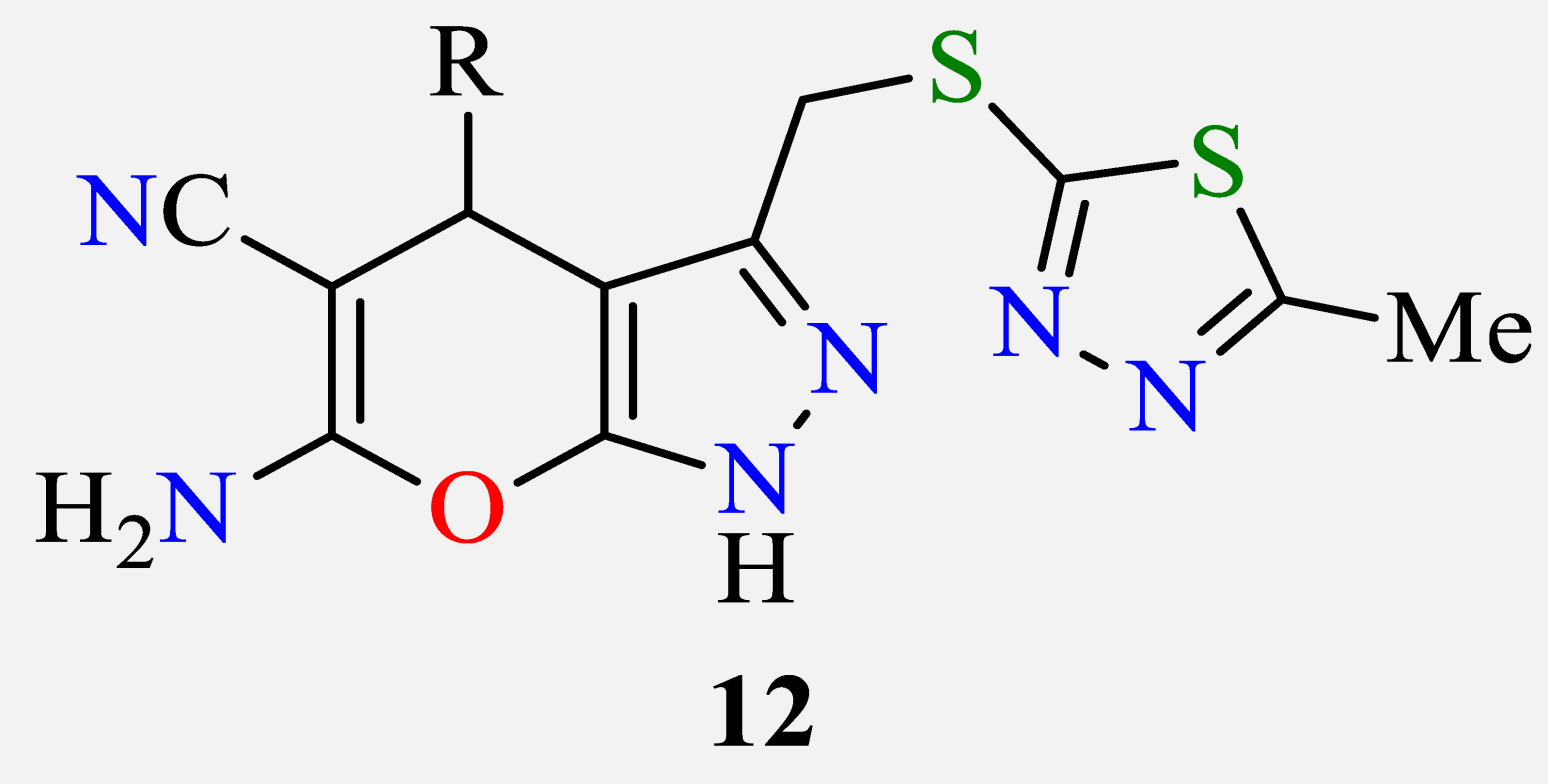					
Compound	R	Zone of Inhibition (mm)			
		*Aspergillus* *flavus*		*Aspergillus* *niger*	
		50 μg/Well	100 μg/Well	50 μg/Well	100 μg/Well
**12a**	C_6_H_5_	15	20	22	25
**12b**	2-MeOC_6_H_4_	5	9	10	12
**12c**	3-OHC_6_H_4_	10	14	16	19
**12d**	4-ClC_6_H_4_	20	24	27	29
**12e**	2-OHC_6_H_4_	11	15	18	20
**12f**	4-NO_2_C_6_H_4_	23	27	30	33
**12g**	4-FC_6_H_4_	22	26	28	31
**12h**	4-OHC_6_H_4_	13	17	19	22
**12i**	4-MeOC_6_H_4_	9	11	13	15
**12j**	2-MeC_6_H_4_	2	6	8	11
**12k**	4-MeC_6_H_4_	7	10	11	13
**12l**	2-ClC_6_H_4_	19	23	25	28
**12m**	3-FC_6_H_4_	18	21	23	26
**12n**	4-BrC_6_H_4_	13	18	21	23
Ketoconazole ^a^	--	28	31	33	35

^a^ Standard drug for the study.

**Table 40:** Antifungal activity of spiropyrrolidine-oxindoles **29**.

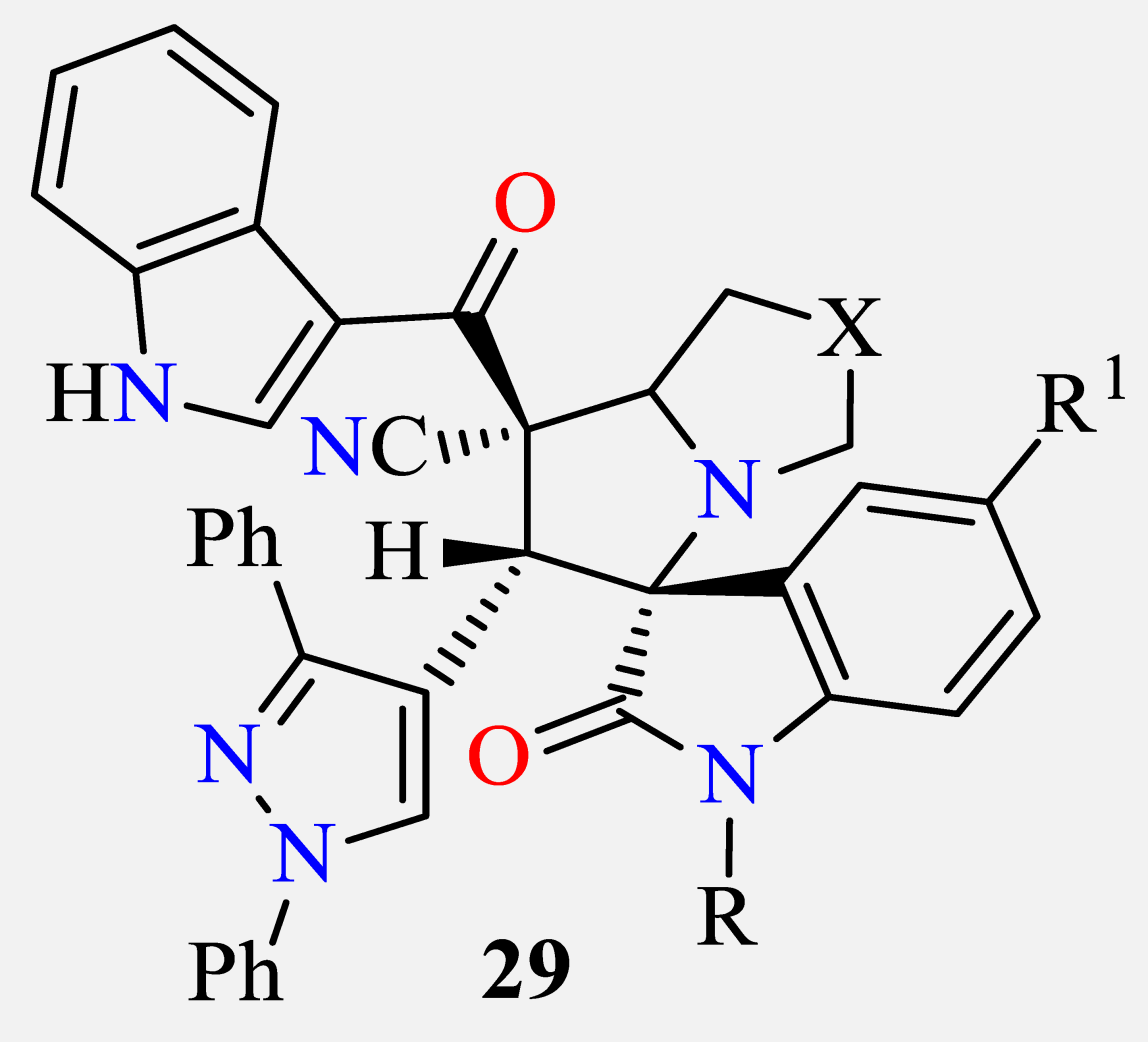							
Compound	R	R^1^	X	*C. albicans*		*M. pachydermatis*	
				CPM (mm)	MIC (μg/mL)	CPM (mm)	MIC (μg/mL)
**29a**	H	H	CH_2_	10	125	8	500
**29b**	Allyl	H	CH_2_	-- ^b^	250	-- ^b^	500
**29c**	*n*-Butyl	H	CH_2_	-- ^b^	500	-- ^b^	-- ^b^
**29d**	Me	H	CH_2_	-- ^b^	-- ^b^	-- ^b^	-- ^b^
**29e**	Propargyl	H	CH_2_	10	-- ^b^	9	-- ^b^
**29f**	H	NO_2_	CH_2_	11	250	-- ^b^	500
**29g**	H	H	S	9	125	8	250
**29h**	Allyl	H	S	12	250	10	-- ^b^
**29i**	Benzyl	H	S	-- ^b^	500	-- ^b^	500
**29j**	*n*-Butyl	H	S	-- ^b^	125	-- ^b^	125
**29k**	Me	H	S	-- ^b^	-- ^b^	-- ^b^	-- ^b^
Ketoconazole ^a^	--	--	--	28	25	26	25

^a^ Standard drug for the study. ^b^ Not determined.

**Table 41:** Antifungal evaluation of pyrazole derivatives **9** and **10**.

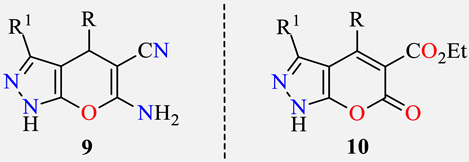					
Compound	R	R^1^	MIC (μg/mL)		
			*Candida krusei*	*Aspergillus fumigatus*	*Aspergillus niger*
**9a**	2-OHC_6_H_4_	Me	5.00	6.25	7.50
**9b**	4-MeC_6_H_4_	Me	7.50	10.0	12.5
**9c**	4-MeOC_6_H_4_	Me	3.75	5.00	7.50
**9d**	C_6_H_5_	Me	5.00	6.25	7.50
**9e**	2-Furanyl	Me	10.0	3.75	7.50
**9f**	2-OHC_6_H_4_	EtO	12.5	8.75	6.25
**9g**	4-MeC_6_H_4_	EtO	8.75	10.0	7.50
**9h**	4-MeOC_6_H_4_	EtO	8.75	7.50	6.25
**9i**	C_6_H_5_	EtO	12.5	10.0	8.75
**9j**	9-Anthracenyl	EtO	12.5	5.00	7.50
**9k**	2-Furanyl	EtO	8.75	7.50	10.0
**10a**	2-OHC_6_H_4_	Me	8.75	7.50	6.25
**10b**	4-MeC_6_H_4_	Me	8.75	12.5	6.25
**10c**	4-MeOC_6_H_4_	Me	6.25	10.0	3.75
**10d**	C_6_H_5_	Me	7.50	5.00	8.75
**10e**	2-Furanyl	Me	3.75	8.75	12.5
Griseofulvin ^a^	--	--	3.12	1.25	1.25
Nystatin ^a^	--	--	1.25	1.00	1.00

^a^ Positive control for the study.

**Table 42:** Antifungal activity of 4-[(3-aryl-1-phenyl-1*H*-pyrazol-4-yl)methylidene]-2,4-dihydro-3*H-*pyrazol-3-ones **34**.

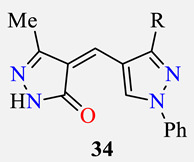			
Compound	R	Zone of Inhibition (mm)	
		*Aspergillus niger*	*Sclerotium rolfsii*
**34a**	C_6_H_5_	9.8	12.8
**34b**	4-FC_6_H_4_	8.2	10.2
**34c**	4-ClC_6_H_4_	6.9	12.0
**34d**	4-BrC_6_H_4_	7.7	9.8
**34e**	4-MeC_6_H_4_	8.0	10.2
**34f**	4-MeOC_6_H_4_	7.8	8.5
**34g**	3-MeOC_6_H_4_	6.8	9.0
**34h**	4-OHC_6_H_4_	6.9	11.3
**34i**	4-NO_2_C_6_H_4_	7.9	10.8
**34j**	2,4-(Cl)_2_C_6_H_3_	8.9	13.2
Ketoconazole ^a^	--	18.3	22.1

^a^ Standard drug for the study.

**Table 43:** Antifungal activity of 3-alkyl-1-(4-(aryl/heteroaryl)thiazol-2-yl)indeno[1,2-*c*]pyrazol-4(1*H*)-ones **37**.

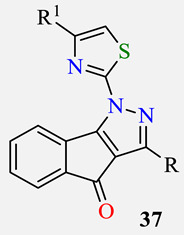				
Compound	R	R^1^	MIC (μmol/mL)	
			*Candida albicans*	*Aspegillus niger*
**37a**	Me	Biphenyl	0.0297	0.0595
**37b**	Et	Biphenyl	0.0144	0.1153
**37c**	*i*Pr	Biphenyl	0.0139	0.1118
**37d**	*i*Bu	Biphenyl	0.0067	0.0270
**37e**	Me	2-Naphthyl	0.0635	0.0635
**37f**	Et	2-Naphthyl	0.0153	0.1227
**37g**	*i*Pr	2-Naphthyl	0.0148	0.0593
**37h**	*i*Bu	2-Naphthyl	0.0071	0.0574
**37i**	Me	2-Benzofuranyl	0.0163	0.0652
**37j**	Et	2-Benzofuranyl	0.0157	0.1258
**37k**	*i*Pr	2-Benzofuranyl	0.0151	0.0607
**37l**	*i*Bu	2-Benzofuranyl	0.0146	0.0293
Fluconazole ^a^	--	--	0.0408	0.0408

^a^ Standard drug for the study.

**Table 44:** Antioxidant activity of dihydropyrano[2,3-*c*]pyrazole derivatives **90**.

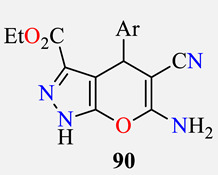					
Compound	Ar	% Inhibition at 25 μg/mL	% Inhibition at 50 μg/mL	% Inhibition at 75 μg/mL	% Inhibition at 100 μg/mL
**90a**	C_6_H_5_	16.16	25.54	37.23	48.45
**90b**	4-MeC_6_H_4_	13.12	23.64	34.86	43.84
**90c**	2-ClC_6_H_4_	15.52	25.13	38.52	47.69
**90d**	4-ClC_6_H_4_	15.86	25.23	36.14	45.70
**90e**	4-FC_6_H_4_	16.26	25.17	35.23	46.81
**90f**	2-Furanyl	14.54	21.22	25.31	32.21
**90g**	2-Thiophenyl	18.14	26.46	39.76	42.11
**90h**	4-EtC_6_H_4_	14.14	24.22	30.52	39.50
**90i**	4-HOC_6_H_4_	22.75	30.46	48.11	60.65
**90j**	2-MeOC_6_H_4_	17.24	26.61	38.54	49.41
**90k**	3-MeO-4-OHC_6_H_3_	21.62	27.42	45.12	57.82
**90l**	4-MeOC_6_H_4_	17.64	24.25	34.36	44.11
**90m**	4-NO_2_C_6_H_4_	17.25	28.41	36.19	45.64
Ascorbic acid ^a^	--	29.34	55.84	90.07	98.85

^a^ Reference compound.

**Table 45:** Multicomponent Debus–Radziszewski synthesis of imidazolylpyrazoles **145** as *α*-glucosidase inhibitors.

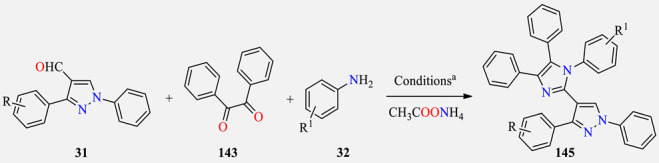					
Compound	R	R^1^	Yield of 145 (%)	*α*-Glucosidase Inhibition	
				Percentage Inhibition (%)	IC_50_ (µM)
**145a**	4-MeO	4-MeO	75	99.56	178.82
**145b**	4-MeO	4-Br	70	91.12	162.93
**145c**	4-MeO	3,5-(Me)_2_	81	96.13	182.17
**145d**	4-Cl	4-MeO	78	98.65	168.92
**145e**	4-Cl	4-Cl	69	93.19	85.71
**145f**	4-Cl	4-Br	73	96.21	25.19
**145g**	4-Cl	3,5-(Me)_2_	84	89.54	132.81
**145h**	4-Br	4-MeO	82	89.76	104.75
**145i**	4-Br	4-Cl	71	87.25	84.61
**145j**	4-Br	3,5-(Me)_2_	81	75.23	412.42
**145k**	3-NO_2_	4-Cl	77	96.76	42.23
**145l**	3-NO_2_	4-MeO	84	95.79	43.14
**145m**	3-NO_2_	4-Br	81	97.52	33.62
**145n**	3-NO_2_	3,5-(Me)_2_	88	91.25	58.73
Acarbose ^b^	--	--	--	92.23	38.25

^a^ Reaction conditions: Pyrazole-4-carbaldehydes **31**, benzil **143**, substituted anilines **32**, and ammonium acetate, AcOH, MWI. ^b^ Reference compound.

**Table 46:** Three-component synthesis and *α*-amylase activity of thiazole–pyrazole hybrids **151**.

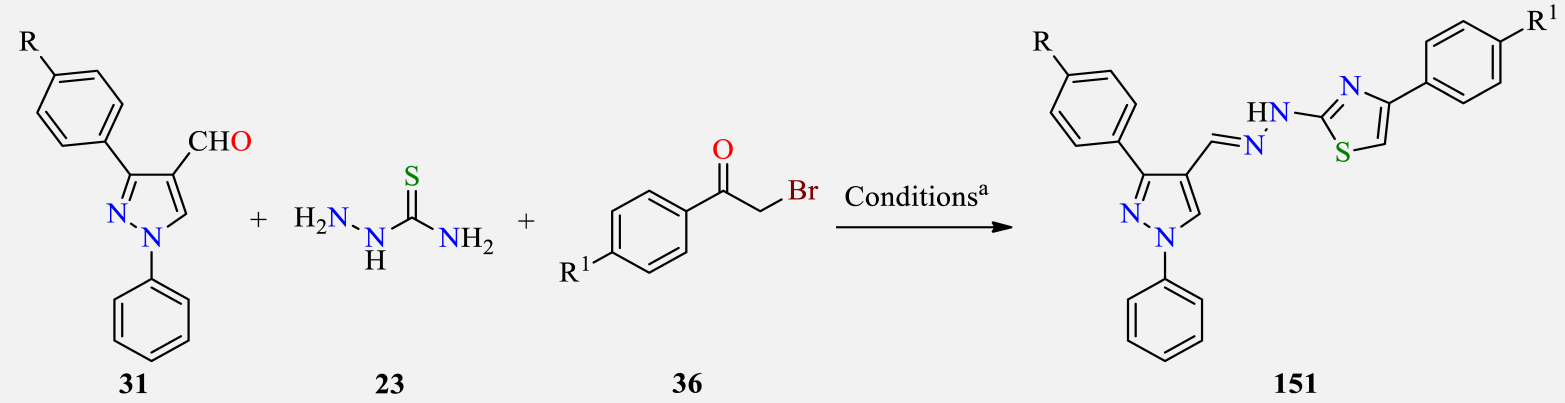						
Compound	R	R^1^	Yield 151 (%)	% Inhibition at 12.5 μg/mL	% Inhibition at 25 μg/mL	% Inhibition at 50 μg/mL
**151a**	Br	Br	85	56.80	61.21	82.72
**151b**	Br	Cl	80	61.03	68.38	80.33
**151c**	Br	NO_2_	88	68.57	71.51	82.90
**151d**	MeO	Br	82	58.82	67.46	79.04
**151e**	MeO	Cl	80	69.49	75.55	82.17
**151f**	MeO	NO_2_	89	55.15	64.71	77.94
**151g**	Me	Br	85	65.81	72.06	89.15
**151h**	Me	Cl	87	62.13	71.69	88.42
**151i**	Me	NO_2_	83	48.35	52.21	79.96
**151j**	F	Br	79	57.35	70.59	81.25
**151k**	F	Cl	86	60.29	64.71	77.21
**151l**	F	NO_2_	78	55.33	67.83	86.03
**151m**	Cl	Br	71	67.28	76.84	83.82
**151n**	Cl	Cl	83	68.87	71.32	84.74
**151o**	Cl	NO_2_	83	56.07	67.10	81.99
**151p**	H	Br	81	52.39	61.40	75.18
**151q**	H	Cl	82	40.99	58.82	70.04
**151r**	H	NO_2_	87	52.94	60.48	78.13
Acarbose ^b^	--	--	--	67.25	71.17	77.96

^a^ Reaction conditions: 1-aryl-3-phenyl-1*H*-pyrazole-4-carbaldehydes **31** (1 mmol), thiosemicarbazide **23** (1.1 mmol), and substituted *α*-bromoacetophenones **36** (1 mmol), EtOH (10 mL), reflux, 5 min. ^b^ Reference compound.

**Table 47:** *α*-Amylase activity of 3-alkyl-1-(4-(aryl/heteroaryl)thiazol-2-yl)indeno[1,2-*c*]pyrazol-4(1*H*)-ones **37**.

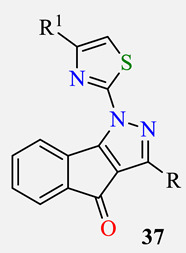			
Compound	R	R^1^	IC_50_ (μM)
**37a**	Me	Biphenyl	5.29
**37b**	Et	Biphenyl	10.05
**37c**	*i*Pr	Biphenyl	20.51
**37d**	*i*Bu	Biphenyl	10.78
**37e**	Me	2-Naphthyl	6.88
**37f**	Et	2-Naphthyl	4.17
**37g**	*i*Pr	2-Naphthyl	18.31
**37h**	*i*Bu	2-Naphthyl	3.21
**37i**	Me	2-Benzofuranyl	0.94
**37j**	Et	2-Benzofuranyl	0.79
**37k**	*i*Pr	2-Benzofuranyl	0.46
**37l**	*i*Bu	2-Benzofuranyl	0.89
Acarbose ^a^	--	--	0.11

^a^ Reference compound.

**Table 48:** Anti-inflammatory activity of 4-coumarinylpyrano[2,3-*c*]pyrazoles **93**.

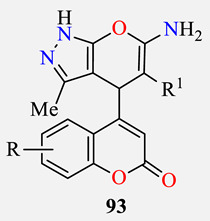				
Compound	R	R^1^	% Inhibition of Egg Albumin in 31.25 μM	% Inhibition of Erythrocyte in 100 μM
**93a**	6-Me	CN	10.33	28.36
**93b**	6-MeO	CN	19.13	32.66
**93c**	6-Cl	CN	28.04	36.06
**93d**	7-Me	CN	7.60	28.16
**93e**	7,8-Benzo	CN	14.44	28.86
**93f**	6-Me	COOEt	24.82	36.06
**93g**	6-MeO	COOEt	51.43	54.06
**93h**	6-Cl	COOEt	39.06	39.86
**93i**	7-Me	COOEt	28.67	36.16
**93j**	7,8-Benzo	COOEt	37.65	38.56
Aceclofenac ^a^	--	--	5.50	--
Acetyl salicylic acid ^a^	--	--	--	36.16

^a^ Reference compound.

**Table 49:** Three-component synthesis of pyrazole-linked triazolo[1,5-*a*]pyrimidines **153** as anti-tubercular agents.

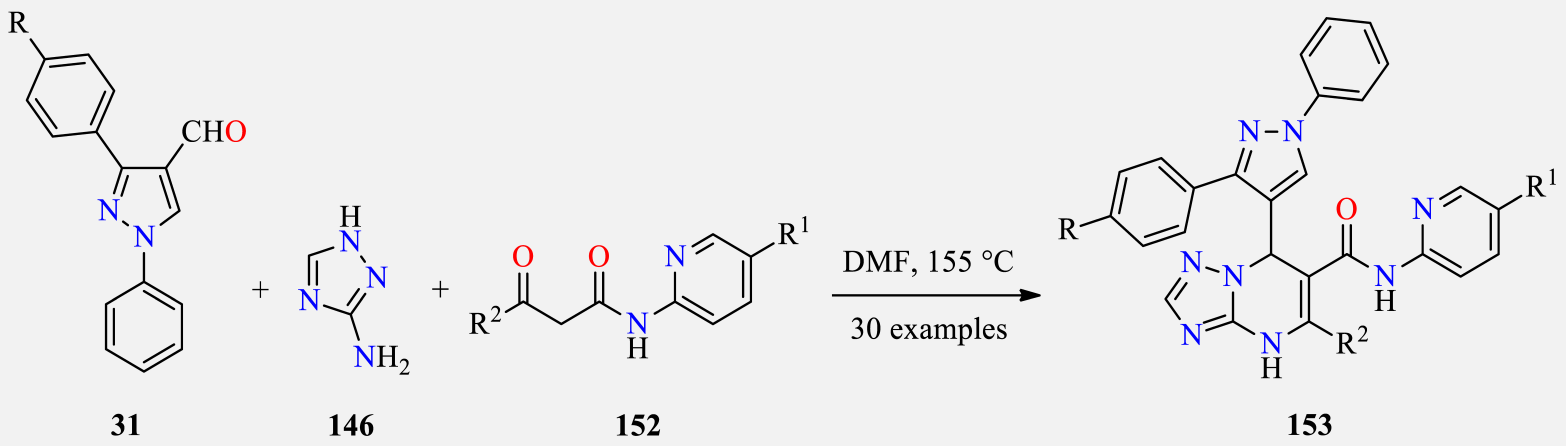							
Compound	R	R^1^	R^2^	% Inhibition at 6.25 μg/mL	MIC (μg/mL)	IC_50_ Enzyme Inhibition (μg/mL)	CC_50_ VERO Cells (μg/mL)
**153a**	H	Br	Me	95	3.13	0.85	25
**153b**	NO_2_	Br	Me	96	1.56	0.28	25
**153c**	Cl	Br	Me	98	0.78	0.11	20
**153d**	F	H	Me	99	0.78	0.16	20
**153e**	F	Br	Me	99	0.39	0.11	20
Isoniazid ^a^	--	--	--	99	0.3	--	--
Rifampicin ^a^	--	--	--	99	0.5	--	--
Ethambutol ^a^	--	--	--	99	3.12	--	--

^a^ Reference compounds.

**Table 50:** Antituberculosis activity of polyhydroquinoline derivatives **51**.

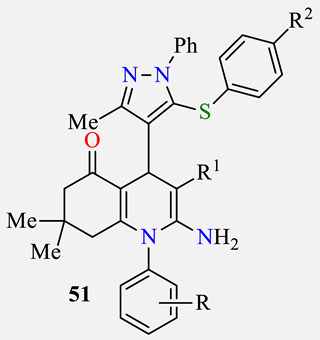				
Compound	R	R^1^	R^2^	% Inhibition at 250 μg/mL
**51a**	4-F	CN	4-Cl	65
**51b**	4-F	COOEt	4-Cl	20
**51c**	4-F	CONH_2_	4-Cl	30
**51d**	4-F	CN	4-Me	46
**51e**	4-F	COOEt	4-Me	94
**51f**	4-CF_3_	CN	4-Me	46
**51g**	4-CF_3_	COOEt	4-Me	50
**51h**	2,4-diF	CN	4-Me	73
**51i**	2,4-diF	COOEt	4-Me	95
**51j**	2,4-diF	CN	4-Cl	79
**51k**	2,4-diF	COOEt	4-Cl	89
**51l**	2,4-diF	CONH_2_	4-Cl	91
**51m**	4-F	CN	4-F	67
**51n**	4-F	COOEt	4-F	80
**51o**	4-CF_3_	CN	4-F	65
**51p**	4-CF_3_	COOEt	4-F	87
Isoniazid ^a^	--	--	--	99
Rifampicin ^a^	--	--	--	98

^a^ Reference compounds.

**Table 51:** Ultrasound-assisted three-component synthesis of 1,2,4-triazol-1-yl-pyrazole-based spirooxindolopyrrolizidines **157** as antitubercular agents.

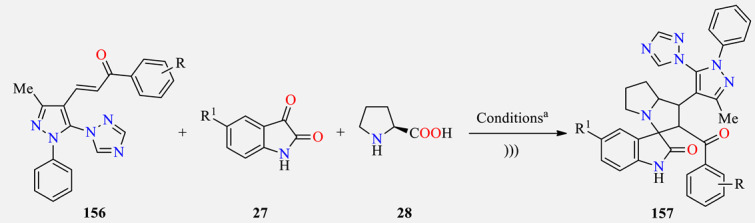					
Compound	R	R^1^	Yield 157 (%)	MIC (μg/mL)	Cytotoxicity % Inhibition at 25 μg/mL ^c^
**157a**	H	H	92	>25	-- ^d^
**157b**	4-Me	H	83	>25	-- ^d^
**157c**	4-MeO	H	78	12.5	-- ^d^
**157d**	4-Cl	H	86	6.25	16.85
**157e**	4-Br	H	84	1.56	27.15
**157f**	3-NO_2_	H	92	>25	-- ^d^
**157g**	4-NO_2_	H	90	6.25	29.61
**157h**	H	Cl	89	0.78	19.76
**157i**	4-Me	Cl	80	25.0	-- ^d^
**157j**	4-MeO	Cl	76	3.12	21.65
**157k**	4-Cl	Cl	82	1.56	17.91
**157l**	4-Br	Cl	81	1.56	26.43
**157m**	3-NO_2_	Cl	90	6.25	18.96
**157n**	4-NO_2_	Cl	88	1.56	24.94
**157o**	H	Br	85	3.12	18.62
**157p**	4-MeO	Br	79	25.0	-- ^d^
**157q**	4-Cl	Br	85	1.56	22.36
**157r**	4-Br	Br	80	1.56	21.97
**157s**	3-NO_2_	Br	86	6.25	20.08
**157t**	4-NO_2_	Br	85	6.25	26.40
Ethambutol ^b^	--	--	--	1.56	-- ^d^

^a^ Reaction conditions: Pyrazole-based chalcones **156** (1.0 mmol), substituted isatins **27** (1.0 mmol), and *L*-proline **28** (1.0 mmol) in [Bmim]BF_4_ (3.0 mL) at 60 °C for 6–16 min under ultrasound irradiation. ^b^ Reference compound. ^c^ The cytotoxicity was determined in the RAW 264.7 cell line. ^d^ Not determined.

**Table 52:** One-pot four-component synthesis and in vitro anticholinesterase activity of new tacrine-pyranopyrazole analogues **161**.

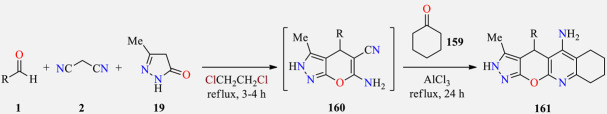			
Compound	R	AChE IC_50_ (μM)	BChE IC_50_ (μM)
**161a**	C_6_H_5_	1.23	36.01
**161b**	4-ClC_6_H_4_	1.66	>68.15
**161c**	4-Br-C_6_H_4_	1.80	11.64
**161d**	4-NO_2_C_6_H_4_	5.80	2.73
**161e**	4-MeSC_6_H_4_	0.058	>66.05
**161f**	2,4-(Me)_2_C_6_H_3_	0.29	39.03
**161g**	2,4-(MeO)_2_C_6_H_3_	0.26	31.11
**161h**	2-Cl-5-NO_2_C_6_H_3_	1.04	2.50
**161i**	2-Cl-6-NO_2_C_6_H_3_	1.77	1.84
**161j**	Biphenyl-4-yl	0.044	>61.20
**161k**	3-Pyridinyl	0.33	4.26
**161l**	2-MeO-1-naphthyl	0.13	11.35
Galantamine ^a^	--	21.82	40.72
Tacrine ^a^	--	0.26	0.05

^a^ Reference compounds.

**Table 53:** Microwave-assisted four-component synthesis of pyrano[2,3-*c*]pyrazoles **162** as non-purine xanthine oxidase inhibitors.

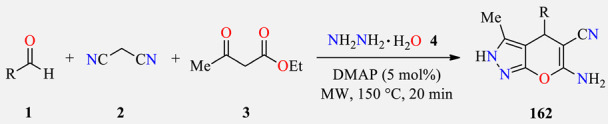			
Compound	R	% Age Inhibition (50 μM) ^b^	XO IC_50_ (μM)
**162a**	C_6_H_5_	83	12.4
**162b**	4-FC_6_H_4_	45	--
**162c**	3-OHC_6_H_4_	24	--
**162d**	3-ClC_6_H_4_	62	--
**162e**	2-OHC_6_H_4_	27	--
**162f**	4-BrC_6_H_4_	85	6.4
**162g**	2-MeOC_6_H_4_	48	--
**162h**	4-MeOC_6_H_4_	38	--
**162i**	4-NO_2_C_6_H_4_	82	8.4
**162j**	4-OHC_6_H_4_	45	--
**162k**	1-Naphthyl	54	--
**162l**	2-Furanyl	84	3.2
**162m**	2-Thiophenyl	89	2.2
**162n**	2-Indolyl	44	--
**162o**	3,4-(MeO)_2_C_6_H_3_	55	--
**162p**	2,3,4-(MeO)_3_C_6_H_2_	52	--
**162q**	4-ClC_6_H_4_	88	4.0
**162r**	4-Pyridinyl	79	--
**162s**	3-Me-2-thiophenyl	74	--
Allopurinol ^a^	--	--	8.29

^a^ Reference compound. ^b^ The compounds exhibiting % age inhibition of more than 80% at 50 μM were further tested for the XO inhibitory activity.

**Table 54:** Three-component synthesis and in vitro antileishmanial activity of pyrazolodihydropyridines **164**.

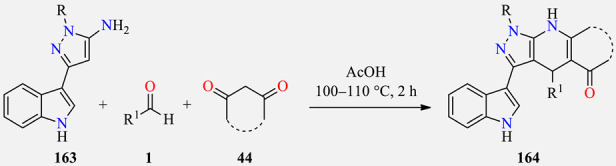							
Compound	R	R^1^	Reagent 44	Promastigotes GI (%)		Amastigotes GI (%)	
				25 μM	50 μM	25 μM	50 μM
**164a**	4-ClC_6_H_4_	4-ClC_6_H_4_	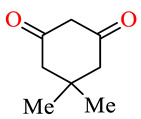	62.5	65.4	NSI ^b^	NSI ^b^
**164b**	4-FC_6_H_4_	4-ClC_6_H_4_	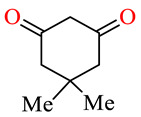	54.3	65.3	NSI	NSI
**164c**	4-ClC_6_H_4_	3,4,5-(MeO)_3_C_6_H_2_	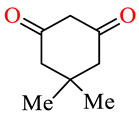	79.3	86.7	23.6	28.2
**164d**	3,4-(Cl)_2_C_6_H_3_	2,5-(MeO)_2_C_6_H_3_	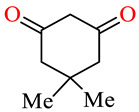	85.9	90.6	91.5	95.8
**164e**	3,4-(Cl)_2_C_6_H_3_	3,4-(MeO)_2_C_6_H_3_	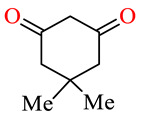	52.3	59.5	NSI	NSI
**164f**	4-ClC_6_H_4_	2-Thiophenyl	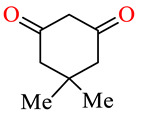	82.5	86.5	43.8	51.7
**164g**	4-ClC_6_H_4_	2,5-(MeO)_2_C_6_H_3_	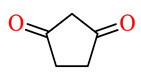	85.5	93.2	37.3	42.6
**164h**	4-ClC_6_H_4_	3,4,5-(MeO)_3_C_6_H_2_	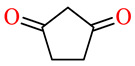	78.4	81.2	45.2	59.6
**164i**	4-ClC_6_H_4_	4-OH-3,5-(MeO)_2_C_6_H_2_	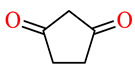	79.1	89.6	25.2	30.7
**164j**	3,4-(Cl)_2_C_6_H_3_	3,4-(MeO)_2_C_6_H_3_	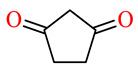	91.7	93.3	96.8	97.3
**164k**	4-FC_6_H_4_	4-OH-3,5-(MeO)_2_C_6_H_2_	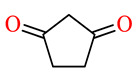	56.5	66.9	NSI	NSI
**164l**	4-ClC_6_H_4_	4-ClC_6_H_4_	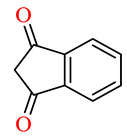	49.1	62.4	NSI	NSI
**164m**	4-FC_6_H_4_	4-ClC_6_H_4_	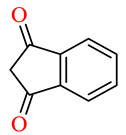	54.2	68.3	NSI	NSI
**164n**	4-ClC_6_H_4_	2,3,4-(MeO)_3_C_6_H_2_	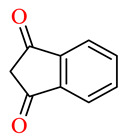	64.2	67.4	NSI	NSI
**164o**	4-ClC_6_H_4_	2-Thiophenyl	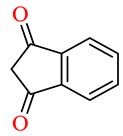	65.3	69.2	NSI	NSI
Miltefosine ^a^	--	--	--	100	100	99.8	100

^a^ Reference compound. ^b^ NSI (no significant inhibition).

## Data Availability

Data sharing is not applicable.
